# Loss of Egg Yolk Genes in Mammals and the Origin of Lactation and Placentation

**DOI:** 10.1371/journal.pbio.0060063

**Published:** 2008-03-18

**Authors:** David Brawand, Walter Wahli, Henrik Kaessmann

**Affiliations:** 1 Center for Integrative Genomics, University of Lausanne, Lausanne, Switzerland; 2 National Research Center Frontiers in Genetics, University of Lausanne, Lausanne, Switzerland; Université de Montréal, Canada

## Abstract

Embryonic development in nonmammalian vertebrates depends entirely on nutritional reserves that are predominantly derived from vitellogenin proteins and stored in egg yolk. Mammals have evolved new resources, such as lactation and placentation, to nourish their developing and early offspring. However, the evolutionary timing and molecular events associated with this major phenotypic transition are not known. By means of sensitive comparative genomics analyses and evolutionary simulations, we here show that the three ancestral vitellogenin-encoding genes were progressively lost during mammalian evolution (until around 30–70 million years ago, Mya) in all but the egg-laying monotremes, which have retained a functional vitellogenin gene. Our analyses also provide evidence that the major milk resource genes, caseins, which have similar functional properties as vitellogenins, appeared in the common mammalian ancestor ∼200–310 Mya. Together, our data are compatible with the hypothesis that the emergence of lactation in the common mammalian ancestor and the development of placentation in eutherian and marsupial mammals allowed for the gradual loss of yolk-dependent nourishment during mammalian evolution.

## Author Summary

Egg yolk contains the nutrients required for the development of the nonmammalian vertebrate embryo. These nutrients derive by and large from a single set of proteins, vitellogenins, which are produced in the liver and provide or transport amino acids, lipids, phosphorous, and calcium to the egg. Mammals have evolved new nutritional resources for their developing and early offspring, such as lactation and placentation. However, the evolutionary timing and molecular events associated with this major phenotypic transition are not well understood. In this study, we have investigated the evolutionary fate of the three ancestral vitellogenin-encoding genes in mammals. Using detailed evolutionary analyses of genomes from the three major mammalian lineages (eutherian “placental” mammals, marsupials, and monotremes), we found that these genes progressively lost their functions and became pseudogenes relatively recently during mammalian evolution (the most recent inactivation event occurred roughly 30–70 million years ago). Monotremes, which lactate yet lay small parchment-shelled eggs, even retained a functional vitellogenin gene, consistent with their intermediate reproductive state. Our analyses also provide evidence that the major milk resource genes, caseins, which have similar functional properties as vitellogenins, appeared in the common mammalian ancestor ∼200–310 million years ago. Based on our data, we suggest that the emergence of the alternative resources for the mammalian young—lactation and then placentation—only gradually reduced the need for egg yolk resources (and hence functional vitellogenin genes) in mammals.

## Abstract 3

The major egg yolk genes, those that express vitellogenins, appear to have progressively lost their functionality during mammalian evolution, probably due to the emergence of the mammalian-specific developmental nourishment resources, lactation, and placentation.

## Introduction

Nutritional reserves that are stored in egg yolk are crucial for the development of the embryo of nonmammalian oviparous vertebrates [1]. In the extant egg-laying (oviparous) species that are closest to mammals—reptiles and birds—the composition of yolk is well known [1,2]. It mainly consists of proteins, lipids, phosphorous, and calcium, most of which are either contained in or transported to the egg by vitellogenin (VTG), which is produced in the liver. Thus, yolk constitutes an essential resource in these species, because these nutrients cannot be provided from the exterior to the developing egg [3].

**Figure 1:**
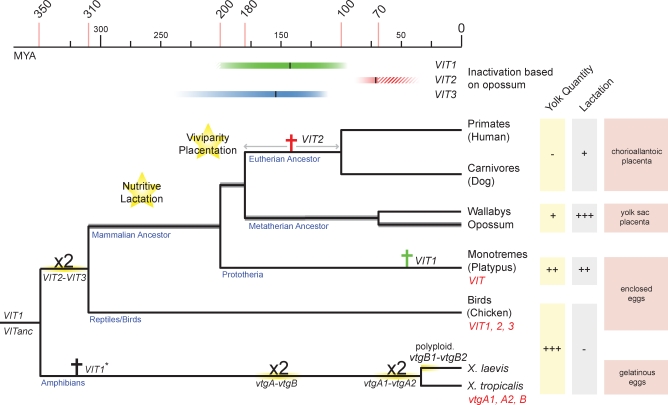
*VIT* Gene Evolution in Tetrapods The topology and divergence times of the tree are based on previous studies [19,24,25,41]. Latin crosses indicate *VIT* inactivation events in eutherians and monotremes. Inactivation estimates (including approximated 95% prediction intervals) based on opossum *VIT* sequences are indicated by colored bars at the top (see also Figure 3). Duplications (“x2”) are indicated. *VITanc* is the likely ancestor of both the amphibian *vtgA1/vtgA2* and *VIT2/VIT3* genes in birds. Functional *VIT* genes in extant species are indicated in red. The inactivation time of VIT1* on the amphibian branch could not be estimated because of its absence in Xenopus tropicalis.

In contrast, “placental” mammals (eutherians) are thought to have replaced the role of VTG through the establishment of a vascularized, chorioallantoic placenta, which builds a controlled interface between the developing embryo/fetus and its mother, together with subsequent milk feeding of the suckling after birth [4–6] (Figure 1).

In marsupials (metatherians), lactation is prolonged and more sophisticated than in eutherians [7,8] (Figure 1). Marsupials also have a placenta, originating from the yolk sac [9], but the marsupial oocyte contains considerably more yolk than that of eutherians [10,11], which is virtually devoid of it. The marsupial yolk reserve is assumed to be essential during the earliest development of the embryo, complementing the uptake of uterine secretions by the yolk sac, prior to shell coat rupture [12]. However, the content of marsupial yolk is not well known [11]. The presence of (transient) yolk-sac placentae [13] and lecithotrophic (yolk-dependent) viviparity in lizards may provide a model for an early form of a still VTG-dependent marsupial. However, the increasing provision of nutrients through more advanced lactation and a placenta during marsupial evolution may have gradually reduced selective pressure to preserve large yolk reserves, which are exclusively designated to the developing embryo/fetus until birth.

Monotremes (prototherians) are the only extant oviparous mammalian species (Figure 1). They possess mammary glands like marsupials and eutherians, but teats are absent and milk is supplied to the offspring by leakage onto the abdominal milk patch [4]. Thus, the combination of a primitive mode of lactation—which is likely similar to that of the common mammalian ancestor [6]—and oviparity in these species may give insights into the relationship between lactation and nutrient reserves in the oocyte, as lactation might have at least partially replaced oocyte resources. Indeed, the eggs (∼2 cm in diameter) of the duck-billed platypus, one of three extant monotreme lineages, are very small in proportion to body size, when compared with, for example, bird and reptile eggs [4]. Nevertheless, monotreme eggs still contain considerable quantities of yolk compared with those of marsupials and eutherians. However, the molecular composition of this yolk is not documented in detail [14].

To understand the transition from yolk-dependent nourishment toward the alternative resources—lactation and placentation—available for the mammalian embryo, fetus, and new-born offspring, we set out to elucidate in detail the evolutionary fate of the genes coding for the fundamental egg yolk resource, VTG, in mammals.

## Results/Discussion

### *VIT* Gene Remnants in Placental Mammals

VTG is encoded by the *VIT* genes, which act in a dosage-dependent manner, so that, for example, a correlation between gene copy number and speed of yolking can be observed (e.g., fish tend to have multiple *VIT* gene copies and produce a larger amount of eggs in shorter time than birds and reptiles [15]). Also, Xenopus laevis, which has a larger body size and lays larger eggs than X. tropicalis, retained a supplemental *VIT* gene copy after polyploidization [16], likely due to increased VTG/yolk requirements.

To be able to assess the fate of *VIT* genes in mammals, we first determined the *VIT* gene complement in available genomes from closely related vertebrate lineages. Our genome analyses confirm that the chicken genome contains three *VIT* genes: *VIT1–VIT3* [17,18]. We previously showed that these stem from two genes—*VIT1* and *VITanc*—which were present at the separation of the amphibian and reptile lineages around 350 million years ago (Mya) [19] (Figure 1). *VITanc* duplicated in tandem early in the common amniote (mammal/reptile/bird) ancestor, yielding *VIT2* and *VIT3*. In addition, it experienced at least two independent duplication events in amphibians [16,20], whereas *VIT1* was lost in this evolutionary lineage (Figure 1).

**Figure 2:**
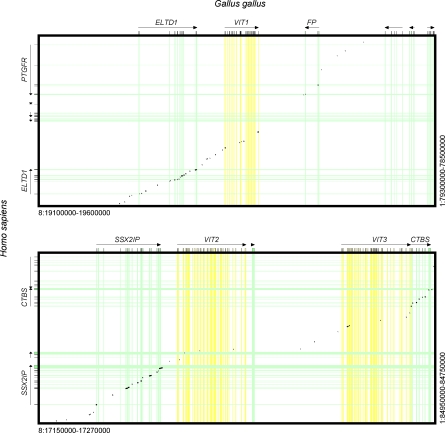
Genome Alignment (Dot Plot Representing SIM Alignments) of Human/Chicken Syntenic Regions *VIT 1-VIT3* Regions The chain with the best cumulative score is shown. Alignment of flanking genes confirms the synteny of the aligned regions. The combined alignments of *VIT1* coding sequences showed significantly higher alignment scores than the genomic background (introns and intergenic regions) in the chain, as assessed by a Mann-Whitney *U* test (*p* < 0.05). Thus, we can statistically exclude that detected *VIT1* remnants from humans represent spurious sequence matches. The coding sequence matches for *VIT2/3* may be too short to provide statistical significance or partially spurious.

Eutherians would be expected to have lost the *VIT* genes during their passage to viviparity. To analyze whether they have indeed completely lost the capacity for VTG production, we screened two representative eutherian genomes of high quality (human and dog) for the presence of *VIT* genes. Interestingly, by using highly sensitive similarity search algorithms [21,22] (see Materials and Methods), we identified a few *VIT* pseudogenic coding sequence remnants (mainly from *VIT1* and *VIT3*) with premature stop codons and frame-shifting insertion/deletions (indels) in regions syntenic to those containing these *VIT* genes in chicken (Figure 2 and Figure S1). Exon 3 of *VIT1* reveals shared indels between human and dog (Figure S2), which indicates that *VIT1* was inactivated before the separation of the human and dog lineages (representing the eutherian superorders Laurasiatheria and Euarchontoglires) ∼90–100 Mya [23] (Figure 1). Generally, the paucity of *VIT* coding sequence remnants in these two eutherian genomes may be suggestive of an early loss/inactivation of these genes prior to the separation of the human–dog split. In an additional screen of low-coverage eutherian genomes, we retrieved *VIT1* exon 3 from armadillo (Dasypus novemcinctus, superorder: Xenartha), which shares two indels with the human/dog sequences (Figure S2). This suggests that *VIT1* was inactivated prior to the divergence of these species.

In summary, it seems that nutritive lactation with complex milk, which evolved since the common mammalian ancestor [6], coupled with a sophisticated placenta, which evolved in parallel with eutherian viviparity, effectively rendered embryonic nourishment through VTG completely dispensable.

### Dating the Inactivation of Marsupial *VIT* Genes

**Figure 3:**
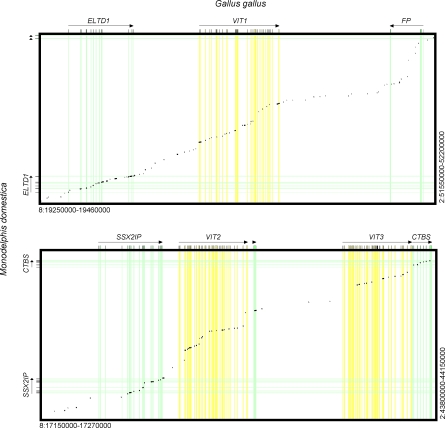
Genome Alignment (Dot Plot Representing SIM Alignments) of Opossum/Chicken Syntenic Regions *VIT1-VIT3* Regions The chain with the best cumulative score is shown. Alignment of flanking genes confirms the synteny of the aligned regions. The subsets of alignments corresponding to *VIT* exons of the best chain for all three regions have significantly higher scores than genomic background hits in the chain (*p* < 0.05, Mann-Whitney *U* test). This shows that *VIT1-VIT3* exon matches in opossum represent nonrandom hits and thus correspond to real coding sequence matches.

**Figure 4:**
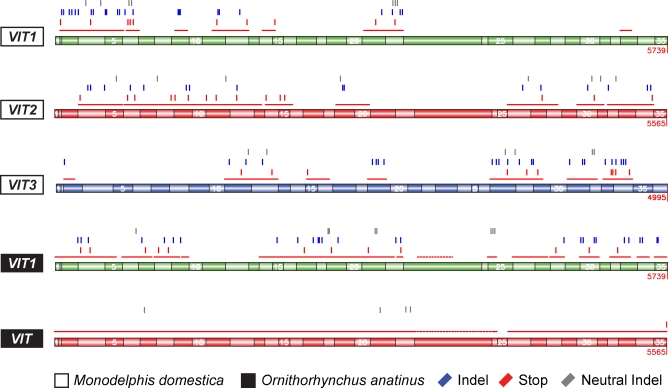
Overview of Intact and Disabled *VIT* Genes from Opossum and Platypus Thin red lines indicate *VIT* sequences that could be retrieved. The sequences are aligned to the known 35 (*VIT1*/*VIT2*) and predicted 36 (*VIT3*) coding exons from the chicken *VIT* genes. Indels (blue), stop codons (red), and neutral indels (gray) are shown (the cumulative indel count is provided in Table 1). We note that the platypus *VIT* gene was aligned to the chicken *VIT2* gene to illustrate the absence of sequence disablements (the platypus *VIT* sequence could not be unambiguously identified as *VIT2* or *VIT3*, see text for details).

To elucidate the fate of *VIT* genes on the metatherian lineage—the mammalian lineage most closely related to that of eutherians (Figure 1)—we analyzed the genome of an American marsupial, the gray short-tailed opossum (Monodelphis domestica). Strikingly, we identified numerous pseudogenic coding sequence remnants with premature stop codons and frame-shifting insertion/deletions (indels) in syntenic regions from all three *VIT* genes known from chicken (Figures 3 and 4, and Figure S3). This result suggests that these genes were all present in the common avian–mammalian ancestor (Figure 1), and that there were probably no independent duplications of *VIT* genes in the mammalian ancestor after the separation of the mammalian–bird/reptile lineages, as no *VIT* sequences could be detected elsewhere in the genome. The detection of coding sequence remnants of many exons from these three genes may indicate a rather recent loss of *VIT* genes in mammals and/or on the metatherian lineage.

**Table 1:**
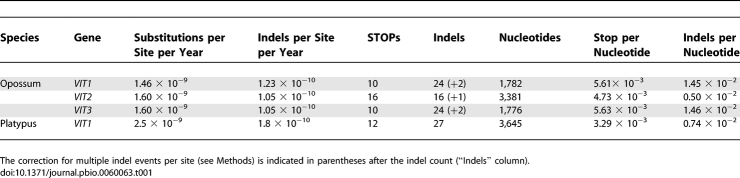
Disablement Counts of *VIT* Genes

**Figure 5:**
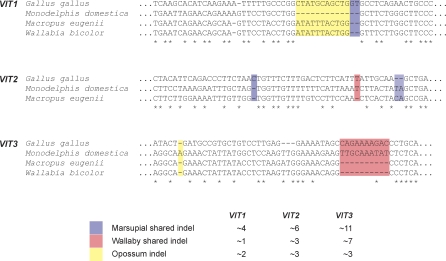
Illustration of Shared Indels in American and Australian Marsupials The total number of non-neutral indels (i.e., those that are not a multiple of 3) shared among marsupials, wallabies (tammar and swamp wallaby), and specific to opossum are shown in the table. They were obtained based on the complete alignment shown in Figure S3. The alignment shown is based on a merge of individual pairwise alignments of the marsupial *VIT* sequences to that of chicken (to preserve the original genomic *VIT* alignments obtained using SIM).

To date the inactivation of these extant *VIT* pseudogenes, we first assessed the status of the *VIT* genes in two Australian marsupial species, the tammar wallaby (Macropus eugenii) and swamp wallaby (Wallabia bicolor). To this end, we retrieved high-quality sequence reads from the tammar wallaby genome project that cover *VIT* exons identified in opossum. In addition, we amplified and sequenced *VIT* exons from the swamp wallaby (based on tammar wallaby sequences) that cover opossum *VIT* exons and their flanks (see Materials and Methods for details). Alignments of *VIT* exons from these two marsupial species, opossum, and chicken, reveal frame-disrupting mutations in the three *VIT* gene sequences from all marsupials, including frame-shifting indels that are shared between the Australian and American marsupials (Figure 5 and Figure S3). The latter strongly suggests that the three *VIT* genes were inactivated before the separation of the Australian and American marsupial lineages—representing the deepest split in the metatherian clade—∼70 Mya [24,25].

**Figure 6:**
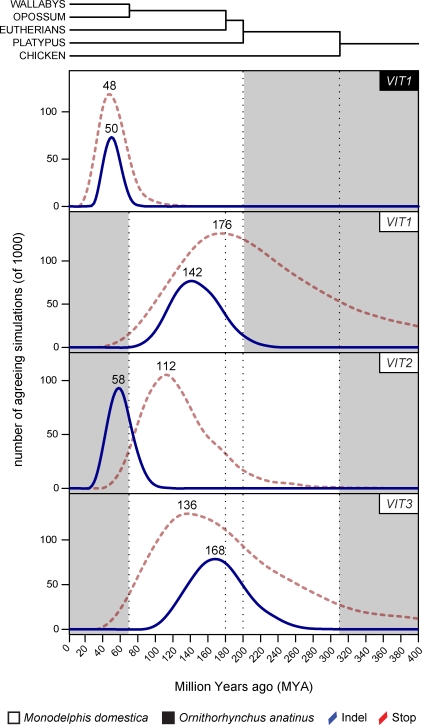
Inactivation Simulations for *VIT* Genes in Monodelphis domestica (opossum) and Ornithorhynchus anatinus (platypus) The fraction of simulations (0–400 Mya) that correspond to the observed stop codon and indel counts for the respective pseudogene are shown (see Materials and Methods and Figure S4 for details on the simulation procedure). The most probable inactivation time is indicated at the mode of the distribution. Shaded areas indicate inactivation times that can be excluded because of the following: (i) shared sequence disablements (*VIT1-VIT3*, providing lower bounds), (ii) non-overlapping distributions (the clear inactivation of *VIT1* in platypus rules out an inactivation of this gene in the common mammalian ancestor), and/or (iii) functionality of all *VIT* genes (*VIT1-VIT3*) in chicken. Dotted lines correspond to major lineage splits as indicated in the phylogenetic tree.

To further date the inactivation of the *VIT* genes beyond the ∼70 million years defined by this phylogenetic dating that was based on the marsupial pseudogene remnants, we used a simulation approach (illustrated in Figure S4, see also Materials and Methods). We first counted the coding sequence disablements of the opossum *VIT* pseudogenes (Figures 4, Figure S3, and Table 1). We then compared these numbers to disablement counts obtained when repeatedly simulating neutral evolution of *VIT* genes for different time periods [26] (Figures 1 and 6). We obtained independent estimates for each gene based on indels and stop codons. Except for *VIT2*, the most probable inactivation estimates are similar for the indel and stop codon analyses (Figure 6). However, given that the lower number of stop codons results in a low time resolution and renders the stop codon–based analysis less precise (Figure 6), we here focus the discussion on the indel-based simulation analysis.

Our simulation-based inactivation analysis suggests that *VIT3* was the first VTG gene in the opossum genome to have lost its function during mammalian evolution. The highest probability of inactivation of *VIT3* is ∼170 Mya, with a 95% confidence interval of ∼110–240 Mya (Figures 1 and 6). Thus, *VIT3* may have been inactivated as early as in the common mammalian ancestor, although it more likely lost its function on the therian (eutherian/metatherian) lineage; either in the common therian ancestor or on the marsupial lineage. Why could functionality of this gene be lost? The loss of *VIT3*-encoded VTG may have been permitted by the advanced lactation of therian mammals and/or placentation, which emerged around the most probable time of inactivation of *VIT3* and was associated with the origin of viviparity and the dramatic reduction in egg (yolk) size in therians [27].

*VIT1* shows the highest probability of inactivation approximately ∼140 Mya (Figures 1 and 6), with a 95% prediction interval of ∼90–200 Mya. Thus, similarly to *VIT3*, *VIT1* was very likely inactivated on the therian lineage; either in the common therian ancestor, or, more likely (given the distribution of predicted inactivation dates), on the marsupial lineage. The latter would imply two independent inactivations of *VIT1* on the eutherian and metatherian lineages. Conceivably, as for *VIT3*, lactation and the early yolk sac placenta—which are still present in all marsupials [9] and transiently in some eutherians [28]—are the new nutritional resources that likely rendered yolk-dependent embryonic/fetal nourishment through *VIT1*-encoded VTG dispensable, allowing for the pseudogenization of this gene.

Among the three *VIT* genes in the opossum genome, *VIT2* appears to be the most recently inactivated gene (Figures 1 and 6). Our indel-based simulation analysis yields ∼60 Mya as the most probable inactivation date, with a prediction interval of ∼30–90 Mya (Figures 1 and 6). When combining this simulation-based estimate with the lower inactivation bound of ∼70 Mya—obtained from our American/Australian marsupial sequence analyses (see above)—we obtain a narrow time window, ∼70–90 Mya, for the inactivation of *VIT2*. Thus, *VIT2* appears to have been inactivated surprisingly recently on the marsupial lineage. This also implies that *VIT2* was inactivated independently in the marsupial lineage and in its eutherian sister lineage (Figure 1). We hypothesize that there may have been a need for some vitellogenization of the oocyte of early marsupials that retarded inactivation of *VIT2*. Probably, some VTG was still necessary to complement a primitive yolk-sac placenta, which may not have permitted sufficient nutrient exchange (analogous to some lecithotrophic but viviparous squamates [13]), and potentially simple milk during early metatherian evolution.

### Functional and Disabled Monotreme *VIT* Genes

To investigate the functional fate of *VIT* genes in the oviparous and lactating monotremes, which represent the most basal mammalian lineage (Figure 1), we analyzed the recently available draft genome from the duck-billed platypus (Ornithorhynchus anatinus). We identified sequences representing two *VIT* genes in this genome (Figure 4 and Figure S5).

One of these is *VIT1*, which, similarly to in opossum, is a pseudogene in platypus. However, interestingly, our analysis suggests that it was inactivated very recently on the monotreme lineage, ∼50 Mya (interval: 30–70 Mya, Figures 1 and 6). The recent inactivation of *VIT1* on the monotreme lineage also corroborates the prediction of an independent inactivation of *VIT1* in therians (Figures 1 and 6), which was based on the simulation analysis of the opossum *VIT1* pseudogene sequence (see above, Figure 6).

We identified a second *VIT* gene in the platypus genome that covers nearly all of the *VIT* coding exons known from chicken. We assembled this sequence from *VIT* exons that are distributed on three complementary supercontigs (Figure S6). We could not unambiguously determine whether this gene corresponds to *VIT2* or *VIT3* (see Materials and Methods), due the incomplete assembly of the *VIT2*/*3* genomic region in platypus. It also remains formally possible that the assembled gene represents a chimeric *VIT2*/*3* gene that originated by fusion of complementary exons from the two genes on the monotreme lineage. We note that a search of platypus trace sequences did not uncover any additional *VIT* gene remnants that are not already contained in the assembled supercontigs, which suggests that the absence of a third *VIT* gene is not due to the incomplete assembly of this genome, but that the remaining *VIT* gene was either completely deleted from the genome during prototherian evolution or degenerated beyond detectability.

**Figure 7:**
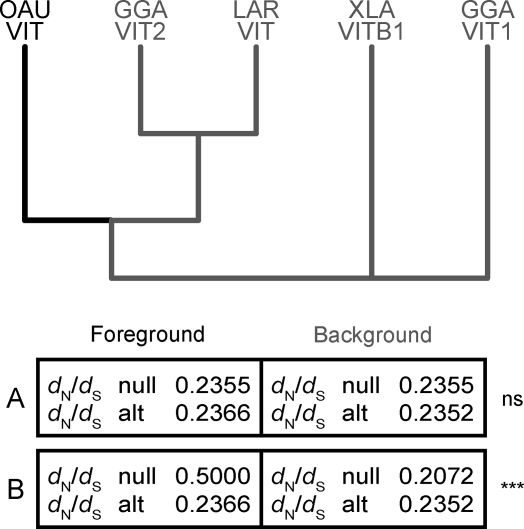
Selection of the Intact Platypus *VIT* Gene Two tests were conducted to test for purifying selection of the intact *VIT* sequence from platypus*.* (A) To test whether the *VIT* platypus (foreground) branch shows a significantly different *d*_N_/*d*_S_ compared to the functional *VIT* lineages from birds and amphibians (background), we used codeml as implemented in PAML and compared a one-ratio model (“null” model, which assumes an equal *d*_N_/*d*_S_ ratio for all the branches in the phylogeny) to a two-ratio model (“alt”, alternative model), where an additional *d*_N_/*d*_S_ value is allowed on the platypus *VIT2* lineage. The two models were compared using a likelihood ratio test [40], and they were found to not provide significantly different fits to the data (“ns”, *p* = 0.96). (B) To test whether *d*_N_/*d*_S_ on the lineage leading to the extant platypus *VIT2* sequence is significantly different from 1, we compared the likelihood of the two-ratio model (alternative model), where *d*_N_/*d*_S_ on this lineage is estimated from the data, to that of a model where *d*_N_/*d*_S_ was fixed to 0.5 (null model).

Strikingly, the assembled *VIT* gene is intact over its entire length (Figure 4 and Figure S5), which suggests that it has been functionally preserved. This notion is supported by our selection analysis. A phylogenetic maximum likelihood analysis of functional *VIT1* and *VIT2* genes from birds, amphibians, and the intact *VIT* sequence from platypus lends statistical support to the notion that the intact platypus *VIT* sequence has evolved under purifying selection (see Materials and Methods for details). It shows that the nonsynonymous (*d*_N_) to synonymous (*d*_S_) substitution rate on the terminal branch leading to the platypus *VIT* sequences is significantly lower than 0.5 (*p* < 10^−6^; Figure 7). It also shows that the rate of amino acid change is not significantly different from that of the *VIT* genes of birds and amphibians (*p* = 0.96). Together, these analyses strongly suggest that the intact platypus *VIT* gene is functional.

Thus, the egg-laying yet lactating monotremes reveal an intermediate state at the genomic level in terms of *VIT* function/inactivation (preservation of one, loss of the remaining functional *VIT* genes) that fits strikingly well with their intermediate reproductive phenotype, which is likely similar to that of the common mammalian ancestor. The diminished quantity of yolk relative to birds/reptiles, and hence the relative lack of nutrients (VTGs) in the platypus/monotreme egg, was probably replaced by nutritive lactation (which emerged in the common mammalian ancestor) of the altricial offspring, as previously hypothesized [6].

Since the composition of monotreme milk has not been well documented [29], one could previously only speculate about its capacity to functionally replace VTG [30]. As caseins are major carriers of calcium (bound to phosphorylated serine residues) in milk, analogous to the phosvitin domain of VTG, and also the major milk protein/amino acid constituents (analogous to VTG in the egg) [31], the presence of casein in monotreme milk would probably be particularly vital to allow for *VIT* gene loss.

### Casein Milk Genes in the Common Mammalian Ancestor

**Figure 8:**
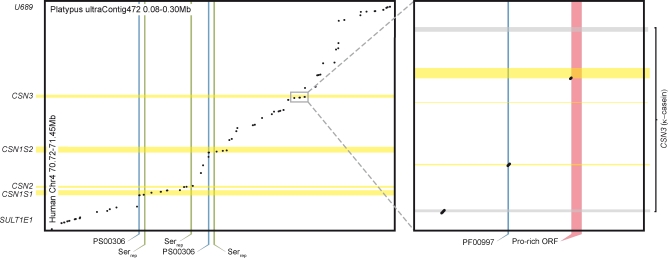
Alignment of Syntenic *SCPP* Regions between Human and Platypus Using SIM Horizontal bars show localizations of known casein genes or exons in humans. Vertical bars indicate various features (specified in the figure) of the putative platypus casein sequences. Putative casein locus sequences were predicted using GenScan, and putative transcripts overlapping significant alignments with SIM were analyzed for serine abundance (for putative α/β-caseins) and casein signatures (PS00306). SIM alignments of the human κ-casein locus were compared to Genewise HMM predictions (with Pfam HMM PF00997, κ-casein).

Previous analyses of duplications in the secretory calcium-binding phosphoprotein (*SCPP*) gene family, encompassing caseins, suggested that the duplications leading to the appearance of the *CSN* casein genes known from therians (*CSN1S1* and *CSN1S2* encoding α caseins; and *CSN2* encoding β casein) occurred around the time of the split of this lineage from that of monotremes [32]. We screened the platypus genome to see whether monotremes do in fact have orthologous casein genes, which would imply that these genes emerged in the common mammalian ancestor. Interestingly, we identified three putative casein genes in a genomic region that is syntenic to that carrying the casein genes in therians (Figure 8). Caseins, and the *SCPP* gene family more generally, evolve rapidly [33], rendering the sequences highly divergent. However, the presence of serine repeats in the putative monotreme casein genes may suggest a potentially high phosphorylation and calcium-binding capacity similar to eutherian caseins.

In addition, our analyses indicate the presence of a proline-rich gene orthologous to the κ casein gene (*CSN3*), which is not homologous to the other caseins. Notably, this gene was shown to be crucial for calcium supersaturation (i.e., the formation of a stable casein micelle) of eutherian milk and for lactation (including maternal lactation behaviour) in general [31,33] (Figure 8). Thus, our analyses indicate that casein genes emerged in the common mammalian ancestor. They might thus have contributed to the increasing role of lactation for the nourishment of the young. This may have reduced the need for egg yolk nourishment, which, in turn, may have reduced the selective pressure to maintain functional *VIT* genes.

### Conclusion

In this study, we investigated the fate of the major egg yolk genes, encoding VTGs, in mammals. Surprisingly, we identified pseudogenic fragments of *VIT* genes in therian mammals (in particular in marsupials) as well as a *VIT1* pseudogene and intact *VIT* gene in platypus.

Our inactivation dating analyses based on comparative sequencing of *VIT* pseudogenes as well as a simulation approach revealed multiple independent inactivation events (e.g., the independent inactivation of *VIT1* in therians and monotremes) and a progressive loss of function of *VIT* genes during mammalian evolution (Figures 1 and 6). We note that the individual estimated inactivation times should be considered approximate, due to the uncertainties in the simulation-based dating approach (including the lineage divergence times that are assumed in this study).

Given the key nourishment role of VTG for the development of the young of egg-laying animals, the progressive loss of function of VTG-encoding genes, as suggested by our analysis, is consistent with the gain of alternative nourishment resources for the mammalian offspring. One of the resources that likely reduced the selective pressure on yolk-dependent nourishment is lactation, which originally had a simple egg-wetting function in ancestral mammals [34] but evolved toward a new nourishment resource for the offspring in the common mammalian ancestor [35]. Similarly to VTG in the egg, lactation likely provided a source of lipids, calcium, phosphorous, and amino acids to the young (hatchlings) already in the late common mammalian ancestor [6]. Calcium, phosphorous, and amino acids could probably already be derived from caseins around that time, as our analyses suggest that casein genes were present in the common mammalian ancestor.

Thus, lactation may represent the initial alternative resource in mammals that rendered some embryonic egg yolk nourishment through VTG dispensable and thus allowed for the loss of *VIT* genes. This hypothesis is supported by our finding that platypus retained a functional *VIT* gene but lost functionality of the remaining *VIT* genes. This *VIT* gene pattern is strikingly consistent with the reduced egg size/yolk quantity and the presence of lactation in this mammal [6]. Given that lactation likely represents the major known alternative/new nourishment resource for the young of monotremes relative to those from birds/reptiles, these observations argue for lactation as the initial resource permitting *VIT* gene loss.

The second major mammalian-specific resource for the young is the placenta, which originated on the therian lineage on the passage to viviparity. Placentation (particularly in eutherians), probably together with more advanced lactation (particularly in marsupials), ultimately rendered VTG egg yolk resources superfluous in therians, allowing for the loss of the remaining *VIT2* gene late on the therian lineage.

In summary, *VIT* genes appear to represent unique markers that reflect the shift of developmental nourishment resources in mammals. Our analyses suggest that while placentation, which arose in the wake of the evolution of mammalian viviparity, appears to have allowed for the complete loss of egg yolk resources (VTGs) in eutherians and marsupials, the emergence of nutritive lactation may have reduced the selective pressure on yolk-dependent nourishment ever since early in the last common mammalian ancestor. Thus, the initial driving force for the reduction in nutrient content of the mammalian egg was probably lactation, the key feature of mammals.

## Materials and Methods

### Genome versions.

In this paper, the versions of the genomes we used are as follows: Ornithorhynchus anatinus Draft Contig Assembly V5.0; Monodelphis domestica MonDom5 assembly; Macropus eugenii (NHGRI and AGRF, Ensembl trace server); Homo sapiens NCBI36; Canis familiaris CanFam 1.0; Gallus gallus WASHUC2 (*VIT* gene builds were retrieved from Ensembl v44); Xenopus tropicalis JGI4 / JGI4.1; and Dasypus novemcinctus (ARMA, May 2005).

### *VIT* region sequences from human, dog, armadillo, and opossum.

Syntenic VTG loci from *Monodelphis* were identified by similarity searches of known adjacent genes (defining syntenic regions) from chicken using WU-BLAST (with standard parameter settings). *VIT* syntenic regions in human and dog were retrieved from the Ensembl database (http://www.ensembl.org). *VIT* sequence remnants from opossum and human/dog/armadillo were detected in alignments of orthologous *VIT* regions from opossum (and human/dog/armadillo) and chicken generated by the SIM alignment tool [21,22]. We used the following SIM alignment parameters, which were obtained from a simulation procedure (see below), for all analyses of our study: I = −0.5, V = −1, O = 4.0, and E = 0.4. The best scoring alignment chain was determined from the 2,000 best local alignments for each *VIT* region using dynamic programming. Importantly, the combined alignments of *VIT* coding sequences of the best chain for each of the opossum *VIT1–VIT3* regions (aligned to chicken) showed significantly higher alignment scores than the genomic background (introns and intergenic regions) in the chain, as assessed by a Mann-Whitney *U* test (*p* < 0.05). This suggests that the obtained (pseudogenic) *VIT* remnants from opossum are nonrandom sequence matches and represent true *VIT* sequences. In the eutherian comparison, *VIT1* coding sequences showed significantly higher scores than the genomic background (the alignable regions for the other two *VIT* genes may be too short to provide statistical significance—*VIT3*,—or represent spurious matches—*VIT2*). All dot plots (also for the other species comparisons below) were generated using the gff2aplot tool [36]. Absence of *VIT* gene sequences outside of *VIT* syntenic regions was confirmed using tBLASTn against the entire genomic sequence.

### *VIT* region sequences from Australian marsupials.

To identify tammar wallaby *VIT* region sequences, all reads from the tammar wallaby genome project were used as queries in BLASTn searches against the opossum *VIT* regions. Tammar wallaby reads (trimmed to exclude low quality bases—Phred scores < 20—and vector sequences) with significant matches were aligned to the opossum genomic *VIT* region sequences using SIM. Based on these alignments (a total of 308 reads for *VIT1* and 80 reads for the *VIT2*/*3* region) and the American–Australian marsupial split time [24,25] (∼70 My), we estimated marsupial-specific indel and substitution rates (using the F84 nucleotide substitution model; other models provided similar estimates) for each *VIT* region for the inactivation dating analysis described below. The mutation rates obtained from this analysis are provided in Table 1. To amplify *VIT* exons from the swamp wallaby (Wallabia bicolor), we designed PCR primers that flank *VIT* exons using Primer3 (http://primer3.sourceforge.net/), based on the tammar sequences (aligned to the opossum *VIT* genome sequence). *VIT* sequences were then amplified from genomic swamp wallaby DNA (extracted from blood, kindly provided by Robert Zingg, Zurich Zoo) using PCR and sequenced using standard procedures (yielding ∼4-fold sequence coverage).

### *VIT* sequences from platypus.

Platypus *VIT*-containing contigs were identified using BLASTn (with the chicken *VIT* cDNA sequences as queries) with relaxed alignment parameters (reduced word size and adjusted scoring: W = 9, M = 1, N = −1, Q = 2, R = 1). We screened for the presence of *VIT* sequences among unassembled trace sequences by searching the platypus trace archive using BLASTn. Final *VIT* sequence alignments were generated between *VIT* genomic sequences from chicken and the ordered and concatenated candidate contigs using SIM, similarly to the procedure used for opossum. The contigs used for the assembly of the *VIT1* pseudogene and intact *VIT* gene are displayed in Figure S6. The reconstructed exon structure of the intact *VIT* gene were complemented with GeneWise [37] exon predictions (using chicken *VIT2* as a template) for confirmation. Coding sequences were extracted and aligned to a set of homologous *VIT* sequences from other species. Based on these alignments, we reconstructed trees using MrBayes (http://mrbayes.scs.fsu.edu/), in order to determine orthologous relationships. This analysis confirmed that the platypus *VIT* pseudogene is orthologous to the chicken *VIT1* gene (Figure S6). The intact *VIT* sequence could not be unambiguously assigned to either *VIT2* or *VIT3* (unpublished data), probably because the duplication event of *VITanc* that yielded *VIT2*/*3* occurred just prior to bird/reptile-mammal split.

### Dating of *VIT* gene inactivations using evolutionary simulations.

To date the inactivation of ancestral marsupial/monotreme *VIT* genes, we used the following simulation procedure (illustrated in detail in Figure S4):

(1) To simulate sequence evolution after *VIT* gene inactivation, we used the functional *VIT1–VIT3* gene sequences from chicken (i.e., the closest functional orthologs of the mammal *VIT* genes) as templates, since the ancestral *VIT* gene sequences at the time of inactivation cannot be inferred. However, we note that the use of different intact starting sequences for the simulations yields only slightly divergent estimates for stop codons (Figure S7), whereas indels are unaffected by sequence composition in our simulation model.

(2) We repeatedly (1,000 times) simulated neutral evolution of these *VIT1*, *VIT2*, or *VIT3* sequences for a given time of inactivation using Reevolver [26] with a Poisson model for the mutational process. The following lineage-specific mutation rates were used for the simulations. For opossum, we used the mutation rates established for the *VIT* regions based on the opossum-tammar wallaby alignments (see above, Table 1). For platypus, we could not obtain region-specific mutation rates, due to the lack of sequences from other monotreme species. Thus, we used 2.5 × 10^−9^ substitutions per site and year (as estimated for the monotreme lineage, [38]) and an indel rate of 1.8 × 10^−10^, which corresponds to ∼1/14 of the substitution rate [39].

(3) After each simulation, the number of potentially disabling indels (that are not a multiple of three) and premature stop codons was recorded.

(4) Steps 2 and 3 were then performed for all inactivation times ranging from 2–500 Mya, respectively (in steps of 2 My).

(5) We calculated the proportion of simulations for each inactivation time for which the same number of indels (stop codons) as that observed in the mammal *VIT* pseudogene was obtained. We corrected the observed indel count for multiple indels at the same site (see below).

(6) The inactivation time with the highest proportion of simulations corresponding to the observed number of disablements was taken to represent the most probable inactivation estimate.

(7) A 95% prediction interval for this estimate was approximated by calculating the time interval (combining the set of simulations for all inactivation times) that contains 95% of all simulations that yielded the observed number of disablements. The combined set of simulations for all inactivation times was used for the plots in Figure 6. Curves were smoothed using the function SPLINES as implemented in the R software package (http://www.r-project.org/).

To obtain optimal SIM alignment parameters, to test the ability of SIM to correctly detect and align ancestral *VIT* sequences (so that the real number of indels is reflected in the alignment), and to be able to correct for potential multiple indel events at a site, we first performed degeneration simulations (250 simulations for each parameter setting) similar to those described above but evolving the entire genomic *VIT1* sequence (exons and introns) from chicken (∼50 kb). We then compared the number of indels added to the *VIT1* locus sequence to the number that can be detected in the degenerated sequence after each round of simulation using SIM alignments (Figure S8). We used nonlinear regression to establish the correction function (see Figure S8). The observed number of indels in *VIT* pseudogene sequences from opossum was corrected using this function (Table 1).

### Casein prediction in platypus.

The syntenic *SCPP* region in platypus was identified with tBLASTn using genes flanking this cluster in the human genome as queries. The syntenic *SCPP* regions of human and platypus were aligned using SIM [21,22]. Putative casein locus sequences were predicted using GenScan (as *CSN* family members are rapidly diverging and GenScan does not require homology information) and putative transcripts overlapping significant alignments with SIM were analyzed for serine abundance (for putative α/β-caseins) and casein signatures (PS00306). SIM alignments of the human κ-casein locus were compared to Genewise HMM predictions (with Pfam HMM PF00997, κ-casein).

### Selection analysis.

To test whether the *VIT* platypus branch shows a significantly different *d*_N_/*d*_S_ compared to the functional *VIT* lineages from birds and amphibians, we used codeml as implemented in PAML and compared a one-ratio model (that assumes an equal *d*_N_/*d*_S_ ratio for all the branches in the phylogeny) to a two-ratio model, where an additional *d*_N_/*d*_S_ value is allowed on the platypus *VIT* lineage. The two models were compared using a likelihood ratio test [40]. To test whether the extant platypus *VIT* sequence has evolved under purifying selection, we compared the likelihood of the two-ratio model—where *d*_N_/*d*_S_ on this lineage is estimated from the data (see above) —to that of a model where *d*_N_/*d*_S_ was fixed to 0.5. We chose 0.5 as a conservative threshold value (a bit higher than the *d*_N_/*d*_S_ value—∼0.24—estimated in the alternative model, Figure 7), indicating relative strong purifying selection on the intact platypus *VIT* gene.

## Supporting Information

Figure S1 Overview of *VIT* Sequence Remnants from Human and Dog Thin red lines indicate *VIT* sequences that could be retrieved. The sequences are aligned to coding exons from the chicken *VIT* genes. Indels (blue), stop codons (red), and neutral indels (gray) are shown. *VIT1* sequence alignments show significantly higher scores relative to the genomic background (*p* < 0.05, Mann-Whitney *U* test).
(393 KB PDF)

Figure S2 Sequence Alignment of the *VIT1* Exon 3 from Human (Homo sapiens), Dog (Canis familiaris), Armadillo (Dasypus novemcinctus), and Chicken (Gallus gallus) The alignment shows two indels that are shared between human, dog, and armadillo, indicating inactivation of these genes in the common ancestor of these species (see main text for discussion).
(29 KB PDF)

Figure S3 Sequence Alignment of *VIT1-VIT3* (Pseudo)Genes from American and Australian Marsupials and Chicken The alignment shown is based on a merge of pairwise alignments of the marsupial *VIT* sequences to that of chicken (to preserve the original genomic *VIT* alignments obtained using SIM). Individual alignable fragments from the different VIT exons are shown. Nucleotides that could not be retrieved from one or both Australian marsupials are marked (“N”).
(74 KB PDF)

Figure S4 Illustration of *VIT* Gene Inactivation Procedure (see also Materials and Methods) (1) To simulate sequence evolution after *VIT* gene inactivation, the functional *VIT1-VIT3* gene sequences are used as template.
(2) Neutral evolution (using parameters provided in Materials and Methods) is simulated for a given time of inactivation using Reevolver [26].
(3) After each simulation, the number of potentially disabling indels (that are not a multiple of three) and premature stop codons is recorded (as exemplified in the table).
(4) Steps 2 and 3 are repeated 100,000 times.
(5) This set of simulations (steps 2–4) is performed for inactivation times ranging from 10–500 Mya (in steps of 10 My).
(6) We then calculated the proportion of simulations for each inactivation time for which we obtained the same number of indels (stop codons) as that observed in the mammal *VIT* pseudogene.
(7) The time with the highest proportion is taken to represent the most probable inactivation estimate.
(231 KB PDF)

Figure S5 Sequence Alignment of the *VIT1* Pseudogene from Platypus with the Intact *VIT1* Gene from Chicken, and the Functional *VIT* Gene from Platypus with *VIT2* from Chicken (54 KB PDF)

Figure S6 Genomic Alignments of Platypus/Chicken *VIT* Regions (Best Chain) The contigs used to assemble these genes is indicated (gray bars with identifiers). A phylogenetic tree based on various chicken (GGA), gull (LAR), African clawed frog (XLA), and platypus (OAN) sequences is shown. It shows that platypus *VIT1* clusters with *VIT1* from chicken, confirming its orthology.
(179 KB PDF)

Figure S7 Simulations Using Different *VIT* Starting Sequences Cumulative stop codon counts are shown for chicken (GGA), gull (LAR), and Xenopus laevis (XLA).
(104 KB PDF)

Figure S8Nonlinear Regression of Observed and Actual Indels per Base Based on the Simulation of Neutral Evolution of the Chicken *VIT1* Region (See Materials and Methods for Details)Nonlinear regression function used to approximate the relationship of observed and real indels: 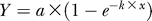 . For the SIM parameter set chosen for the alignments of our study (see Materials and Methods): *a* = 0.0278 ± 0.002, k = – 41.358 ± 3.352.
(165 KB PDF)

## Abbreviations

Mya: million years ago
VTG: vitellogenin
