# Supplementary material for: Loss of Egg Yolk Genes in Mammals and the Origin of Lactation and Placentation
Source: PLoS Biol. 2008 Mar 18;6(3):e63. doi: 10.1371/journal.pbio.0060063 (PMC2267819; doi:10.1371/journal.pbio.0060063)
Supplement: Supplementary file 1 [file pbio.0060063.sg001.pdf]

Figure S1

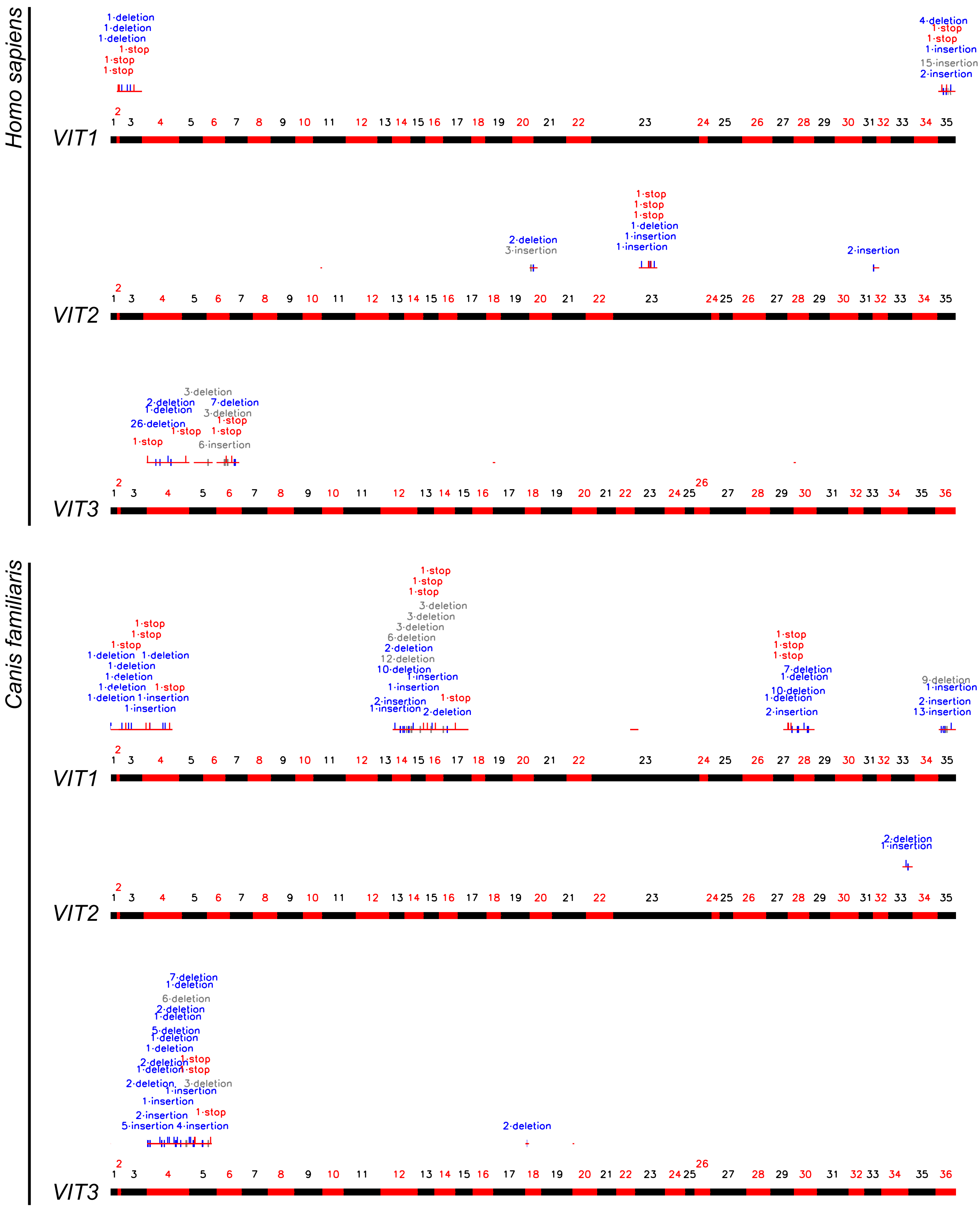

Homo sapiens
VIT1
VIT2
VIT3
Canis familiaris
VIT1
VIT2
VIT3
