# Supplementary material for: Loss of Egg Yolk Genes in Mammals and the Origin of Lactation and Placentation
Source: PLoS Biol. 2008 Mar 18;6(3):e63. doi: 10.1371/journal.pbio.0060063 (PMC2267819; doi:10.1371/journal.pbio.0060063)
Supplement: Supplementary file 2 [file pbio.0060063.sg002.pdf]

Figure S2

```
Gallus gallus          AACCTGATTTTGGTGAAAATAAGGTTTATACATACAATTATGAAAGCATACTTTTCAGTG
Homo sapiens           AACCTAACTTCCA-GAAAGGGTGACTTATGATTCTAAATATGAAAGTACA-TTTCTAGTG
Canis familiaris       AACTTAACTTCAG-AAAGGGACGATTTATAGTTCTAAATAAGAAAGGATA-TTTCTGATG
Dasypus novemcinctus   AACCTAACTTCCAGAAAGGGAAGATTTATAGTTCTAAATATGGAAGTATA-TTTCCAGTG
                       *** * * **     **     *  ****   *  ** ** * *** * * ***    **

Gallus gallus          GTATTCCAGAGAAAGGACTTGCAAGAACTGGAATAAGGATAAGAAGTGAAGTGGAAATTA
Homo sapiens           TATTTCCACA-ACAGTACTTAGAAGACCTGGGATGTAAACAAAAAGTAAAGTAGAAGTCA
Canis familiaris       GACGGCCACA-ATCATGCTTGGAAAAACTGGGATGTGGGTGAAAAGTAAAGTAGAAATCA
Dasypus novemcinctus   GACTGCTACA-ACAGTACTTGGAAGAGCTGGGATATAAATGAGAAGTACAGTAGAAATCT
                            * * * *     ***  ** * **** **        * ****  *** *** *

Gallus gallus          GTGGTATTGGACCAAAACTTTGTCTTATTAGG
Homo sapiens           CTGGCACAGATCTGAAACCAAGTTTTATTAAG
Canis familiaris       CTGGCCCTGATCTAAAACCCAGTCTCATTAGG
Dasypus novemcinctus   CTGGCACTGTTCTAAAATCGAGTCTTATTAGT
                        ***    *  *  ***    ** * ****
```
