# Supplementary material for: Loss of Egg Yolk Genes in Mammals and the Origin of Lactation and Placentation
Source: PLoS Biol. 2008 Mar 18;6(3):e63. doi: 10.1371/journal.pbio.0060063 (PMC2267819; doi:10.1371/journal.pbio.0060063)
Supplement: Supplementary file 3 [file pbio.0060063.sg003.pdf]

Figure S3

a. *VIT1*

Pairwise SIM alignments to chicken sequence

Gallus gallus ATGAGGGGTTTAATA-TCAGCCCTGGTTTTAACCCTGGTG---G
Monodelphis domestica TTGTAGAAAAAAATAATCATCTCTAAATCCAACC-TAATT---T
Macropus eugenii TTGT-GATAAACGTAGTAATCTCTAAATCCAACC-TAACTAATT
Wallabia bicolor NNNNNNNNNNNNNNNNNNNNNNNNNNNNNNNNNNNNNNNNNNNN

Pairwise SIM alignments to chicken sequence

Gallus gallus GGAGTCAGCATCTTAATTACC
Monodelphis domestica GGAGTCAACAACTTAATTATG
Macropus eugenii NNNNNNNNNNNNNNNNNNNNN
Wallabia bicolor NNNNNNNNNNNNNNNNNNNNN

Pairwise SIM alignments to chicken sequence

Gallus gallus AACCTGATTTTGGTGAAAATAAGGTTTATACATACAATTATGAAAGCATACTTTTCAGTG
Monodelphis domestica ATATAGATACACACACACACACACACACACACACAA-CTATAAAAGCAC-CCTATAAATG
Macropus eugenii NNNNNNNNNNNNNNNNNNNNNNNNNNNNNNNNNNNNNNNNNNNNNNNNNNNNNNNNNNNN
Wallabia bicolor NNNNNNNNNNNNNNNNNNNNNNNNNNNNNNNNNNNNNNNNNNNNNNNNNNNNNNNNNNNN

Gallus gallus GTATTCCAGAGAAAGGACTTGCA-AGAACTGGAATAAGGATAAGAAGTGAAGT-GGAAAT
Monodelphis domestica GACTGCCTAAAAAAGAAATTGGGGAGAGTTGGGATAAAACTGAAAAGTAAAGTCAAAAAT
Macropus eugenii NNNNNNNNNNNNNNNNNNNNNNNNNNNNNNNNNNNNNNNNNNNNNNNNNNNNNNNNNNNN
Wallabia bicolor NNNNNNNNNNNNNNNNNNNNNNNNNNNNNNNNNNNNNNNNNNNNNNNNNNNNNNNNNNNN

Gallus gallus TAGTGGTATTGGACCAAAACTTTGTCTTATTAGG
Monodelphis domestica CTATGGCAAATGTCCAAAATTCAGTCTTATTAGG
Macropus eugenii NNNNNNNNNNNNNNNNNNNNNNNNNNNNNNNNNN
Wallabia bicolor NNNNNNNNNNNNNNNNNNNNNNNNNNNNNNNNNN

Pairwise SIM alignments to chicken sequence

Gallus gallus AATTGAAGCAGCAGAGTACAAT---GGCATCTGGCCTACAAGCTCATTCTCTCGGTCACT
Monodelphis domestica AGTTGAGGTGACTGAGTATAAATAAGGTAAATGGCCAAGAGATACATTTGTTCATTCACC
Macropus eugenii NNNNNNNNNNNNNNNNNNNNNNNNNNNNNNNNNNNNNNNNNNNNNNNNNNNNNNNNNNNN
Wallabia bicolor NNNNNNNNNNNNNNNNNNNNNNNNNNNNNNNNNNNNNNNNNNNNNNNNNNNNNNNNNNNN

Gallus gallus CAAACTGACTCAGGCACTAACTGGACAGCTTAGTATTCCCATCAAGTTTGAGTATAGCAA
Monodelphis domestica AAAGCTCACCTAGTCTCT-----GGCATCCTAGGAA-----------------ACAACAT
Macropus eugenii NNNNNNNNNNNNNNNNNNNNNNNNNNNNNNNNNNNNNNNNNNNNNNNNNNNNNNNNNNNN
Wallabia bicolor NNNNNNNNNNNNNNNNNNNNNNNNNNNNNNNNNNNNNNNNNNNNNNNNNNNNNNNNNNNN

```
Gallus gallus          TGGCCACGTTGGAAACCTTATGGCTCCTGATTCTGTCTCAGATGATGGTCTGAACATCTA
Monodelphis domestica  AGATCAGGATGGAACCATTTATGCCCTAGATTCAGTTTCAGATGT---TCTGAATTTTTA
Macropus eugenii       NNNNNNNNNNNNNNNNNNNNNNNNNNNNNNNNNNNNNNNNNNNNNNNNNNNNNNNNNNNN
Wallabia bicolor       NNNNNNNNNNNNNNNNNNNNNNNNNNNNNNNNNNNNNNNNNNNNNNNNNNNNNNNNNNNN


Gallus gallus          CAGAGGGATTCTCAATATTCTGGAGCTCAGTCTGAAGAAGATGCAGCATTCATACAGTAT
Monodelphis domestica  TAAACATAGCCTTAACATATTGGAAATTAGTATCAA-AAGA--CAAAAACCCTACAGTTT
Macropus eugenii       NNNNNNNNNNNNNNNNNNNNNNNNNNNNNNNNNNNNNNNNNNNNNNNNNNNNNNNNNNNN
Wallabia bicolor       NNNNNNNNNNNNNNNNNNNNNNNNNNNNNNNNNNNNNNNNNNNNNNNNNNNNNNNNNNNN


Gallus gallus          ACAGGAG
Monodelphis domestica  GCCAGAG
Macropus eugenii       NNNNNNN
Wallabia bicolor       NNNNNNN
```

Pairwise SIM alignments to chicken sequence

```
Gallus gallus          GCTGGGATTGGAGGTATTTGTAACACAACGTATGCAATTCAAGAAAACAAGAAAGCAAAC
Monodelphis domestica  TCTAGAATTGGTGATCTATTTACCACAACATATTTTATTGAAGAAGATATGAAAGCTGAT
Macropus eugenii       NNNNNNNNNNNNNNNNNNNNNNNNNNNNNNNNNNNNNNNNNNNNNNNNNNNNNNNNNNNN
Wallabia bicolor       NNNNNNNNNNNNNNNNNNNNNNNNNNNNNNNNNNNNNNNNNNNNNNNNNNNNNNNNNNNN


Gallus gallus          CTAGTTGATGTGACCAAATCCAAGGACCTGAACAGTTGTGAAGAGAAAGTCCAAGTGGTC
Monodelphis domestica  TTTGTTTCTATAACTAGA----AGAACTTAAATAACT--GAGGAGAAGGTCAAAATGAAT
Macropus eugenii       NNNNNNNNNNNNNNNNNNNNNNNNNNNNNNNNNNNNNNNNNNNNNNNNNNNNNNNNNNNN
Wallabia bicolor       NNNNNNNNNNNNNNNNNNNNNNNNNNNNNNNNNNNNNNNNNNNNNNNNNNNNNNNNNNNN


Gallus gallus          ACAGGTTCAGCATACACT
Monodelphis domestica  TCTGGTTCTACTTATTCT
Macropus eugenii       NNNNNNNNNNNNNNNNNN
Wallabia bicolor       NNNNNNNNNNNNNNNNNN
```

Pairwise SIM alignments to chicken sequence

```
Gallus gallus          AGAAACAAGAATTCCCGGGCAACTGCTACCTATAATTACAAAATTAAATACACTCATAAT
Monodelphis domestica  CCCAACAGAAATTCCTAGGCCACT------TCAAGTTAAAAAATAAAA---ACTTATATC
Macropus eugenii       NNNNNNNNNNNNNNNNNNNNNNNNNNNNNNNNNNNNNNNNNNNNNNNNNNNNNNNNNNNN
Wallabia bicolor       NNNNNNNNNNNNNNNNNNNNNNNNNNNNNNNNNNNNNNNNNNNNNNNNNNNNNNNNNNNN


Gallus gallus          GAAG----CTGTAATCACACAAGCTGAAGTCGAGGAAGTCCACCAGTTTACACCTTTCCA
Monodelphis domestica  AAAGAGTCCTTGAGTTCTTCAAGCTGAAGTTCATGAAATAGATCAATTTGTACCCTTTAA
Macropus eugenii       NNNNNNNNNNNNNNNNNNNNNNNNNNNNNNNNNNNNNNNNNNNNNNNNNNNNNNNNNNNN
Wallabia bicolor       NNNNNNNNNNNNNNNNNNNNNNNNNNNNNNNNNNNNNNNNNNNNNNNNNNNNNNNNNNNN


Gallus gallus          TGAGATAAC
Monodelphis domestica  TAAGCTGAC
Macropus eugenii       NNNNNNNNN
Wallabia bicolor       NNNNNNNNN
```

Pairwise SIM alignments to chicken sequence

```
Gallus gallus          GCGCTACCTTTT-GGATTTACTTCCTGCA-GCAGCCAGTCACCGTTCACTGA--GATTCT
Monodelphis domestica  GTGCTTATTTTT-AGATATACTCTCTGCCTGTGGCCTGT-ATAGAAGACTTGTTGGTTCT
Macropus eugenii       GTGCATATTTTTTAGATATTCTCCCTGCC-ATGGCCAGT-ATAGAAGACTAGGTGGTTCT
Wallabia bicolor       NNNNNNNNNNNNNNNNNNNNNNNNNNNNNNNNNNNNNNNNNNNNNNNNNNNNNNNNNNNN


Gallus gallus          TGAGGCATAAAATGGAAAGGCAAGAGCTCACCAACTGGGAAATAGCTCAGACAGTGCTTG
Monodelphis domestica  CAGGGTATAAAATTGAAAAGGAAGAAATTAGAGAATGGGAGGCAGCTCAGGCTATACTCC
Macropus eugenii       CAAGGTATAAAATTGAAAAGGCAGAAATTAGAGAATGGGAGGCAGTTCAGGCTCAACTCT
Wallabia bicolor       NNNNNNNNNNNNNNNNNNNNNNNNNNNNNNNNNNNNNNNNNNNNNNNNNNNNNNNNNNNN


Gallus gallus          TG
Monodelphis domestica  TG
Macropus eugenii       TG
Wallabia bicolor       NN
```

Pairwise SIM alignments to chicken sequence

```
Gallus gallus          CTTTGAAAGCCCTTGGAAATGTAGGACATCCAGCCAGCATCAAGCACATCAAGAAA-TTT
Monodelphis domestica  CTTTGAAATCCATAGGGAATAAAGGACATGGGGCCAGTCTGAATTAGAACAGAAAAGTTC
Macropus eugenii       CTTTGAAAGCCATGGGGGATGAAGAACATGTAGCCAGTCTGAATCAGAACAGCAA-GTTC
Wallabia bicolor       CTTTGAAAGCCATGGGGGATGAAGAACATGTAGCCAGTCTGAATCAGAACAGCAA-GTTC
                       ******** ** * **  **  ** ****   *****  * **  * * **  **  **

Gallus gallus          TTGCCCGGCTATGCAGCTGGTGCCTCAGAACTGCCCCTCAAGGTCCATGAAACTGCTGTG
Monodelphis domestica  CTGCCTGG-------------GCTTCTGGGCTTCCCATCAGAATTCTGATTGTTGCTGTC
Macropus eugenii       CTACCTGGATATTTACTGG--GCTTCTTGGCTTCCCAACAGAATTCTAATTGTCATTGTT
Wallabia bicolor       CTACCTGGATATTTACTGG--GTTTCTTGGCTTCCCAACAGAATTCTAATTGTCGCTGTT
                        * ** **             *  **    ** ***  **   * *          ***

Gallus gallus          ATGGCTCTAAAAAGCATAGGCATGAGAGATCCCAAAATG
Monodelphis domestica  ACATCCATGAGAAACAGAGCCAAGAGAAATTCCAAACAG
Macropus eugenii       ACATCCATAAGAAACATAGTCAAGAGAAACTTCAAACAG
Wallabia bicolor       ACATCCATGAGAAACATAGTCAAGAGAAACTTCAAACAG
                       *   *  * * ** ** ** ** **** *   ****  *
```

Pairwise SIM alignments to chicken sequence

```
Gallus gallus          GTCCAGGCTATTACCCTTGAGATTTTCCTGAATCACAAAATTCATCCTCGAATCCGAATG
Monodelphis domestica  GTTCAAGAAATAGTTCTTCATATATTCTTTAATACCAACATCCCTCCCCAAGACAGAATT
Macropus eugenii       GTTCAAGAAATAGCTCTTCATGTGTTCCTCAATACTGATGTCCCTCCCCAAGATGAAATA
Wallabia bicolor       GTTCAAGAAATAGCTCGTCATGTGTTCCTCANTACTGATGTCCCTCCCCAGGATGAAATA
                       ** ** *  **    * * *  * *** * * *    *  * * *** *       ***

Gallus gallus          TTAGCTGCAGTGGTTTTGCTTGAAACTAAGCCAG--------GTCT--------TCCAAT
Monodelphis domestica  GTTCTTTGTCTGGTTCTATTGGAAACTATACCTG--------AGCT--------AATAAT
Macropus eugenii       GTTCTTTCTCCAGCTTTATTGGAAACTGTTGGAGGGAGGACAAGAA--------AACAAA
Wallabia bicolor       GTTCTTTCTCCAGCTTTATTGGAAACTGTTGGAG--------GGAGGACAAAAAAACAAA
                        *   *      * * *  * ******      *                       **
```

```
Gallus gallus          CCTGATGATATTAGTTGATGCTGTGCTGAAGGAACCTAGCATGCAAGTGGCAAGTTTCAT
Monodelphis domestica  --TGACAACCTTGGTCAAGGCCATGTCGAAAGACCACAGCCTGCAAGCGTCCAATTTCTC
Macropus eugenii       --TGGCAGCTTTGGACAAGGCTTTGGTGAAAGATCACAGACTGCAAGTAGCCAGCTTGAC
Wallabia bicolor       --TGGCAGCTTTGGACAAGGCTTTGGTGAAAGATCACAGACTGCAAGTAGCCAGCTTGAC
                         **      ** *   * **  **  *** ** *  **  ******   * *  **


Gallus gallus          TTACTCTCACCTGAGGGCCCT---GGGCAG
Monodelphis domestica  CTACTCCTAAATGAGAGCTCT---GAGGAG
Macropus eugenii       CTGCTCCCAAATGAAGGCTCTAGGGAGAAG
Wallabia bicolor       CTGCTCCCAAATGAAGGCTCTAGGGAGAAG
                        * ***  *  ***  ** **   * * **
```

Pairwise SIM alignments to chicken sequence

```
Gallus gallus          AGTTCCTTATGAGTGGACTGGCAGCTAAATATTTTGTACTGAATAATGCAGGAAGCTTAA
Monodelphis domestica  ATTCTCCGATGGCAGGATTTACCCTGAAATATGACTCAATAAAGGAGACAGGAAGTACAT
Macropus eugenii       AATTGCTAATGGCAGGATTCGCCTTAAAATACTATTCATTAAATAAAGCAGGCAGCACAT
Wallabia bicolor       NNNNNNNNNNNNNNNNNNNNNNNNNNNNNNNNNNNNNNNNNNNNNNNNNNNNNNNNNNNN


Gallus gallus          TTCCAACAATGGCAGTGTCCCAGCTGCGGACACATTTCCTTGGAAGAGTTGCTGAT
Monodelphis domestica  TTCCATCAGGGGTCCTTTCTACATTAAAGGCAAACTTTCTTGGAAACAGAGCTGAT
Macropus eugenii       TACCACCCGAGGTTATTATTGCATTAAAGGCAAGCTTCCATGGNNNNNNNNNNNNN
Wallabia bicolor       NNNNNNNNNNNNNNNNNNNNNNNNNNNNNNNNNNNNNNNNNNNNNNNNNNNNNNNN
```

Pairwise SIM alignments to chicken sequence

```
Gallus gallus          GACAGACAGTCTGTAGAAGATGTTTCATCTGGAAATTCCTTCTCCTTTGGACATCCTTCT
Monodelphis domestica  GAGACACCATCTCCAGAAATCATTTCTAAGGGAAAACTCTATTCCAGTAAAAAGTCATGC
Macropus eugenii       NNNNNNNNNNNNNNNNNNNNNNNNNNNNNNNNNNNNNNNNNNNNNNNNNNNNNNNNNNNN
Wallabia bicolor       NNNNNNNNNNNNNNNNNNNNNNNNNNNNNNNNNNNNNNNNNNNNNNNNNNNNNNNNNNNN


Gallus gallus          TCCGGGAAGGAGCCATTTATTCAGTCCATGTGCTCCAACGCAAGTACATTTGGGGTTCAA
Monodelphis domestica  AGTACAATCATCCCATTGTTTAAAACAATCTGTTTTGATATGAGCATCTTCTGATTCCAG
Macropus eugenii       NNNNNNNNNNNNNNNNNNNNNNNNNNNNNNNNNNNNNNNNNNNNNNNNNNNNNNNNNNNN
Wallabia bicolor       NNNNNNNNNNNNNNNNNNNNNNNNNNNNNNNNNNNNNNNNNNNNNNNNNNNNNNNNNNNN


Gallus gallus          GTGTGCATTGAGAAGAAAAGTGTACATGCAGCATTCATCAGAAATGTGCCTCTTTATAAC
Monodelphis domestica  GTTTGCTTTGAAAGCATAAGTATCGTAGCTTCGTGCTTCAAAGATAT-CTCCCTCACAAT
Macropus eugenii       NNNNNNNNNNNNNNNNNNNNNNNNNNNNNNNNNNNNNNNNNNNNNNNNNNNNNNNNNNNN
Wallabia bicolor       NNNNNNNNNNNNNNNNNNNNNNNNNNNNNNNNNNNNNNNNNNNNNNNNNNNNNNNNNNNN


Gallus gallus          GCTATTGGAGAACATGCCCTTAGAATGAGCTTCAAGCCAG
Monodelphis domestica  GACATCGGGGAACATTATATTAAAGTGACTGCTCAAGGAG
Macropus eugenii       NNNNNNNNNNNNNNNNNNNNNNNNNNNNNNNNNNNNNNNN
Wallabia bicolor       NNNNNNNNNNNNNNNNNNNNNNNNNNNNNNNNNNNNNNNN
```

Pairwise SIM alignments to chicken sequence

```
Gallus gallus          TCT--ACTCAGATGTACCTA-----TTGAAAAAATACAAGTCACAATTCAAGCAGGAGAT
Monodelphis domestica  TCCTCATTCCTTTGTTCCTAACAAATCCAAGAAATTCTATTCACATTCCATACATCA---
Macropus eugenii       NNNNNNNNNNNNNNNNNNNNNNNNNNNNNNNNNNNNNNNNNNNNNNNNNNNNNNNNN---
Wallabia bicolor       NNNNNNNNNNNNNNNNNNNNNNNNNNNNNNNNNNNNNNNNNNNNNNNNNNNNNNNNN---
```

```
Gallus gallus          CAAGCTCCTACAAAAATGGTACGTTTAGTAACATTTGAAGATCCAGAG
Monodelphis domestica  -AAGCATCTCCTAAATTGAGGTCATTAAAAA----TGAAGATCTTGAG
Macropus eugenii       -NNNNNNNNNNNNNNNNNNNNNNNNNNNNNNNNNNNNNNNNNNNNNNN
Wallabia bicolor       -NNNNNNNNNNNNNNNNNNNNNNNNNNNNNNNNNNNNNNNNNNNNNNN
```

Pairwise SIM alignments to chicken sequence

```
Gallus gallus          GTTACTGTTGCTTCATGGATGAGAGGAAAGACCTGTGGAGTCTGTGGAAATAATGACAGA
Monodelphis domestica  ATTACCACTTCTTCATGGATGAAAAGGAAGATCTGTGAAATTTATAGGAAAGCCATTTGG
Macropus eugenii       NNNNNNNNNNNNNNNNNNNNNNNNNNNNNNNNNNNNNNNNNNNNNNNNNNNNNNNNNNNN
Wallabia bicolor       NNNNNNNNNNNNNNNNNNNNNNNNNNNNNNNNNNNNNNNNNNNNNNNNNNNNNNNNNNNN
```

```
Gallus gallus          GAAAAGCACAATGAACTCCTAATGCCAAATCATAAGCTGGCACACAGCTGTTCTGCTTTT
Monodelphis domestica  AAGGCTCAAGATGAACTGGAAAGACCCAAACAGAACCAGGCTCACGGCTGCATTAATCTT
Macropus eugenii       NNNNNNNNNNNNNNNNNNNNNNNNNNNNNNNNNNNNNNNNNNNNNNNNNNNNNNNNNNNN
Wallabia bicolor       NNNNNNNNNNNNNNNNNNNNNNNNNNNNNNNNNNNNNNNNNNNNNNNNNNNNNNNNNNNN
```

```
Gallus gallus          GTTCATTCATGGGTATTGCTAGAAGAAACCTGTTCTGGTG
Monodelphis domestica  -TTAGGAAACAGAGACAGACAGACAGAGGAAAAGAAAGAA
Macropus eugenii       NNNNNNNNNNNNNNNNNNNNNNNNNNNNNNNNNNNNNNNN
Wallabia bicolor       NNNNNNNNNNNNNNNNNNNNNNNNNNNNNNNNNNNNNNNN
```

Pairwise SIM alignments to chicken sequence

```
Gallus gallus          ATG---TG-----CTT---GAAGAAA---GTAAGCTTCCTCTATTGCATCCAAAGTCAAA
Monodelphis domestica  ATG---TA-----CTT---GAAAAGACACATAAGCTAATGCATTTCCAT--AAAACCTAA
Macropus eugenii       TTAGCCTT-----CTT---GAAAAGACACATAAGCTAATGCATTTCCAT--AACATCTAA
Wallabia bicolor       TTT---TC-ccttCTTcttGARAARACATATAARCTAATGCRTTTCYMT--AAAATCTAA
                        *    *      ***   **  * *    *** **    *  **   *  **   * **
```

```
Gallus gallus          TGCATTATGTACAGTCTGCACTGCTTAATAAATATGTTGTTCATTAAATAAGTA
Monodelphis domestica  T-CATTATGTACAGTTTG---TTTTTAATAAATGTGTTGCTCATTAAATACGCA
Macropus eugenii       TGCATTATGTACAGTTTG---TTTCTAATAAATGTGTTGCTCATTAAATACCCA
Wallabia bicolor       TGCATTATGTACAGTTTG---TTTCTAATAAATGTGTTGCTCATTAAATACGCA
                       * ************* **   *   ******** ***** **********   *
```

b. *VIT2*

Pairwise SIM alignments to chicken sequence

```
Gallus gallus          GTCCGCTCTCCACAAGTGGAGGAGTACAATGGGGTCTGGCCCAGGGATCCCTTCACTCGA
Monodelphis domestica  CTTCAGGCTCCATAACTAGAAGAATGCACCAGTTTCTGGAGTAAAGATTCCTTCATTTAC
Macropus eugenii       ATTCAGTCTCCACAAGTAGAAGAATACACTGGTATCTGGCCTAAAGATTCATTCATCTAT
Wallabia bicolor       CTGTAGTCTCCAGTAGAAGAAGACTACAGTGGTAT---GCGTCAAGATTCATTCATCTAT
                        *     *****  *   ** ** * **   *  *   *      *** * ****


Gallus gallus          TCTTCCAAAATCACCCAAGTGATCT-CATCGTGTTTCACCCGGCTCTTC---AAATTTGA
Monodelphis domestica  TCACCTAAGCCCACTAAGGCAAT-T-CATCTGGCCTTATCCAAATAT-----AAGCTTGA
Macropus eugenii       TCACCTAAGCTCACTCAGACAAT-TGCATCTGGCTTTATGCAAATATTC---AAGTTTGA
Wallabia bicolor       TCATCTCAGATCATTAATCCAAC-AGCATCATGCGGTTTTCAAATAATCAAGAAGTTGGA
                       **  *  *   **   *    *    ****  *       *   *       **  * **


Gallus gallus          ATACAGCAGTGGACGGATCGGAAACATTTATGCCCCAGAAGACTGCCCAGATCTGTGTGT
Monodelphis domestica  GAATAATAATGGGCACATTGGCAACTTCTATGCCCCAAAAGTCACCTCAACTATCATTGT
Macropus eugenii       GTATAATAACGGACTAATCGGCAACATCTATGCCCCAAAGGACACCCCAGCTATCTCTGT
Wallabia bicolor       CTTCAATAGTGTA---ATAGTCAACAACATTGCTGCAACAAACGCCCCACCAATTTTTTT
                           *  *  *     ** *  ***     ***  **     *  * **    *   * *


Gallus gallus          TAACATAGTGAGAGGAATATTGAACATGTTCCAGATGACCATTAAAAAATCACAGAACG-
Monodelphis domestica  TAACATAATAAGTGGAATACTAAATATGTTCCATTTTACCATCAAAAAGTCTCAAGATAG
Macropus eugenii       TAACATAGTAAGAGGGATACTAAATATGTTCCATATCACCATCAAGAAGTCTCAAAATAT
Wallabia bicolor       TGTCATCGTAGGAAGGATGATAAATATGTTGCTTATCATCACCAAGAAGTAGTATAA--A
                       *  ***  *  *  * **  * ** ***** *   * * **  ** ** *   *  *


Gallus gallus          -TCTACGAATTACAAGAG
Monodelphis domestica  ATCTATTACTTGCAAGAG
Macropus eugenii       AT--ATTACTTACAAGAG
Wallabia bicolor       ATTTATTATTAACAACAG
                        *  *  * *  *** **
```

Pairwise SIM alignments to chicken sequence

```
Gallus gallus          GCTGGAATTGGAGGTATTTGTCATGCAAGGTATGTCATTCAGGAAGACAGGAAGAATAGC
Monodelphis domestica  GCCAGGATTGGTAGTGTATGTTATACCAATTATATCATCCATGAAGATAAGATAAATAAT
Macropus eugenii       NNNNNNNNNNNNNNNNNNNNNNNNNNNNNNNNNNNNNNNNNNNNNNNNNNNNNNNNNNNN
Wallabia bicolor       NNNNNNNNNNNNNNNNNNNNNNNNNNNNNNNNNNNNNNNNNNNNNNNNNNNNNNNNNNNN


Gallus gallus          CGAATCTATGTTACCAGAACTGTGGACTTGAATAATTGCCAGGAAAAGGTGCAAAAAAGC
Monodelphis domestica  AAGGTTTCTATCATGAAATCCAGAGATCT---CAACTGCCAGGACAAGGCAGTCAGAAAG
Macropus eugenii       NNNNNNNNNNNNNNNNNNNNNNNNNNNNNNNNNNNNNNNNNNNNNNNNNNNNNNNNNNNN
Wallabia bicolor       NNNNNNNNNNNNNNNNNNNNNNNNNNNNNNNNNNNNNNNNNNNNNNNNNNNNNNNNNNNN


Gallus gallus          ATTGGAATGGCTTACATCTATCCCTGCCC
Monodelphis domestica  ATAGGAATGGCTTATCTACATGTCTTTCC
Macropus eugenii       NNNNNNNNNNNNNNNNNNNNNNNNNNNNN
Wallabia bicolor       NNNNNNNNNNNNNNNNNNNNNNNNNNNNN
```

Pairwise SIM alignments to chicken sequence

```
Gallus gallus         AAAGAAAGGCTCACCAAAGGGACCACCGCTTTCTCCTACAAGCTGAAGCAGTCAGACAGC
Monodelphis domestica AAAAGTGAGATTGTGAAAGGAACAGAGACATAGTTCTACAAAATGAAGTATATAAA-AGG
Macropus eugenii      AAAGCTGGGATTGTTAAAGAAACAGAGACATACTCCTACAAAATGAAGTATACAGG-ACA
Wallabia bicolor      NNNNNNNNNNNNNNNNNNNNNNNNNNNNNNNNNNNNNNNNNNNNNNNNNNNATGAA-ATT
                                                                               *


Gallus gallus         -GGCACGCTGATCACAGATGTCTCGTCGCGGCAGGTGTATCAGATCTCCCCATTCAATGA
Monodelphis domestica -GGGGCTCTCATCACAGAAATTATGGTTCACCAGGTATAACAAGTCCCCCTATTCAATAA
Macropus eugenii      TGGGGCTTTGATCACAGAAGTTACAGTTCACCAGATATACCAAGTCTCCCCATTCAATGA
Wallabia bicolor      -GGTAAATTGATCAC-GAGCTTACAGTACACCATATATACTTTTTGTCCGCATTAAATGT
                       **     * ***** **  *       *  **  * **     *  **  *** ***


Gallus gallus         GCCCACTGGGGTGGCTGTCATGGAAGC---AAG
Monodelphis domestica AGAGAATGGGGTTGATGTGACAGATAC---TAT
Macropus eugenii      AGAGAACGGGGTTGGAGTGACAGAAAC---TAA
Wallabia bicolor      GCCGACTGGAGTTGGATGGACAGAAAAAAGCAA
                          *  ** ** *     *  **       *
```

Pairwise SIM alignments to chicken sequence

```
Gallus gallus         ACAGCAGCTCACTTTGGTCGAGGTGAGAAG-TGAGCGGGGCAGTGCCCCGGATGTCCCCA
Monodelphis domestica ACAAAACATCAGCTTGATGGAAGTGAAAAAATAAATATTTGAGCTCCCTAAAAGTTCAAA
Macropus eugenii      GCAAAACCTCAGCTTGATCGAAGTGAAAAACGAGA-GTTTGAGCTCCCCAGAAGTTCTAT
Wallabia bicolor      GCAAGACCTCAGCTTGATCGAAGT-AAAAAACGAGAGTTTGAGCTCCCCAGAAGTTCTAT
                       **  *  ***  *** * ** ** * **            **  ***   * ** *


Gallus gallus         TGCAGAACTATGGCAGCCTTCGCTACCGCTTCCCAGCCGTACTGCCACAGATGCCACTTC
Monodelphis domestica TGCAGAATCAGATAAGTCTTCAGTACCCTTTTCCAACCACCAGACATCAGATGCCTTTTC
Macropus eugenii      TGCAGAATCAGGGAAACCTTCAGTACCGTTTCCTAACCACCAGACACCAGATGCCTTTCC
Wallabia bicolor      TGCAGAATCAGGGAAACCTTCAGTACCATTTCCTAACCACCAGACACCACATGCCTTTCC
                      *******  *    *  ****  ****  ** * * **      *  ** *****  * *


Gallus gallus         AGCTGATCAAGACAAAA------------------------------AACCCTGAGCAA
Monodelphis domestica AGTTGGTAAAAATAAAT------------------------------TCATTTCATCAG
Macropus eugenii      AGCTAGTGAAAATGAATAACCCCAAAAGGCAGGTATGAAACAGGCTTTTCACTTAATCAG
Wallabia bicolor      AGCTAGTGAAAATGAATGACCCCAAAAGGCAGGTATGAAATGGGCTTTTCACTTAATCAG
                      ** *  * ** *  **                                     * * **


Gallus gallus         -------------CGG
Monodelphis domestica -------------CAG
Macropus eugenii      GTTCTTTGAGGGCTAG
Wallabia bicolor      GTTCTTTGAGGGCTAG
                                     *
```

Pairwise SIM alignments to chicken sequence

```
Gallus gallus         ATAATAGAAACGCTGCAGCACATAGTCCTGAATAACCAACAAGATTTCCATGACGATGTT
Monodelphis domestica ------GAGGTACTCCAGCACCTCCTTCAGAACCACCAAGGAACATTCCATGAAGATGCT
Macropus eugenii      ------GAGGTCCTCCAGCACCTCATCCAGAATAACCAAGAAACATTCCATGAAGATGCT
Wallabia bicolor      ------GAGGTCCTCCAGCACCTCATCCAGAATAACCGAGAAACATTCCATGAAGATGCT
                            **    ** ****** *  * * ***  *** *  *   ******** **** *
```

```
Gallus gallus          TCATACAGATTCTTAGAGGTGGTCCAGCTTTGCCGGATAGCAAATGCTGACAATCTTGAG
Monodelphis domestica  CCAGCCAGATTTCTCAAACTCATCCAGCTCATTCAAATGATGGATGCTAGAAGTCTGGTC
Macropus eugenii       CCCACCAAACTTCTAGAGCTTATCCAGCTGATTCAGTCAATGGATGTCGGAAGCCTGGCC
Wallabia bicolor       CCCACCAAACTTCTAGAGCTTATCCAGCTGATTCGGTCAATGGATGTCGGAAGCCTGGCC
                        *   ** * *  *  *  *  *******    *         ***     *  ** *


Gallus gallus          TCTATCTGGAGACAAGTTTCAGATAAACCTCGTTACAG
Monodelphis domestica  AATGTTTGAAAGCAGTGGAAAGACAAGCCTCAATATTG
Macropus eugenii       AGCGTTTGGAAGCAGTGGGAAGACAGGACTCAGCACAG
Wallabia bicolor       AGCGTTTGGAAGCAGTGGGAAGACAGGACTCAGCACAG
                           * ** *  **      *** *   ***   *  *
```

Pairwise SIM alignments to chicken sequence

```
Gallus gallus          TGGCTCCTGAGCGCAGTTTCTGCGAGTGGCACCACAGAAACACTAAAATTCCTTAAGAAC
Monodelphis domestica  TGATTGCTGAGTGTCATCTCTATGGCTGGAACCAAAGATATCTTGAAGTTCACCAAGCAA
Macropus eugenii       TGGTTGCTGACTGCCATCACCATGGCAGGAACCAAAGACACCCTGAAACTCATCAAGCAA
Wallabia bicolor       NNNNNNNNNNNNNNNNNNNNNNNNNNNNNNNNNNNNNNNNNNNNNNNNNNNNNNNNNNNN


Gallus gallus          AGAATTCGCAATGATGACCTCAACTACATTCAGACCCTTCTAACTGTTTCTTTGACTCTT
Monodelphis domestica  AAAATTGGGAGTGAATTCTTCTCCTTCCTAAAGAATTTGCTAG-TGGTTGTTTTTTTCAT
Macropus eugenii       GAAATTGAGAGTGATGCCTTCTCCTTCTTGGAAAATTTGTTGG-TGGTTGTTTTGTCCTT
Wallabia bicolor       NNNNNNNNNNNNNNNNNNNNNNNNNNNNNNNNNNNNNNNNNNNNNNNNNNNNNNNNNNNN


Gallus gallus          CATTTATTGCAA--GCTGATGAACACACACTTCCAATAGCAGCA
Monodelphis domestica  TAAATCTTACTATAGCTGATGCCAATGTCCTTCCCATAGCTGCT
Macropus eugenii       CCAA-CTCACTACAGCCGACACCAGTGTCCTTCCCATAGCTGCA
Wallabia bicolor       NNNNNNNNNNNNNNNNNNNNNNNNNNNNNNNNNNNNNNNNNNNN
```

Pairwise SIM alignments to chicken sequence

```
Gallus gallus          GATTTAATGACCAGCTCTCGAATTCAGAAAAATCCTGTGCTTCAGCAAGTGGCCTGCTTG
Monodelphis domestica  AAAATTGTCGCCAGTCCTCAAATACAGAAATATTTCTTGCTTCAAAAAGTGGCTCTCCTA
Macropus eugenii       GAAATTGTCACCAGTCCTCAAATACAGAAATATCCCTTGCTTCAAAAGGTTGCTCTCCTG
Wallabia bicolor       GAAATTGTCACCAGTCCTCAAATATAGAAATATCCCTTGCTTCAAAAGGTTGCTCTCCTG
                        *  *  *  ****  *** ***  ***** **    *******  * ** **   * *


Gallus gallus          GGATATAGCTCTGTAGTCAACAGATACTGCTCTCAGACCTCAGCATGTCCTAAGGAAGCT
Monodelphis domestica  GCTTATGGCTCTACGGTTTATAGGATGTATCTGCCCACTTCATCATCTCTTGCTGAAACC
Macropus eugenii       GCCTACGGCTCTATGGTTTACAGGTCATATCTTTTCACTTCAGCATCTCCTGCTGAAGCC
Wallabia bicolor       GCCTACGGCTCTATGGTTTACAGGTCATATCTTTTCACTTCAGCATCTGCTGCTGAAGCC
                       *  **  *****   **  * **    *        ** *** *** *  *   *** *


Gallus gallus          CTTCAG
Monodelphis domestica  CGTTAG
Macropus eugenii       CTTCAG
Wallabia bicolor       CTTCAG
                       * * **
```

Pairwise SIM alignments to chicken sequence

```
Gallus gallus          TCCATGACCTGGCAGATGAAGCAATCAGCAGGGGCCGTGAAGACAAAATGAAATTAGCTC
Monodelphis domestica  TCCATAATTACACAAATGAAGCAATTAAAAATGGCCAGCAAGAAGCAATGGTCATAGCAA
Macropus eugenii       NNNNNNNNNNNNNNNNNNNNNNNNNNNNNNNNNNNNNNNNNNNNNNNNNNNNNNNNNNNN
Wallabia bicolor       NNNNNNNNNNNNNNNNNNNNNNNNNNNNNNNNNNNNNNNNNNNNNNNNNNNNNNNNNNNN


Gallus gallus          TAAAGTGCATTGGTAACATGGGAGAACCAGCCAGCTTAAAGCGCATCCTGAAGTTCCTTC
Monodelphis domestica  TGAAGGTCCTTGGCAACTCGGGCTAAGTGGCCAGCATCAAATGCATCCAGAAGCACCTGC
Macropus eugenii       NNNNNNNNNNNNNNNNNNNNNNNNNNNNNNNNNNNNNNNNNNNNNNNNNNNNNNNNCTGC
Wallabia bicolor       NNNNNNNNNNNNNNNNNNNNNNNNNNNNNNNNNNNNNNNNNNNNNNNNNNNNNNNNNNNN


Gallus gallus          CAATATCTTCATCCAGTGCTGCTGATATCCCAGTCCACATTCAGATAGATGCCATAACGG
Monodelphis domestica  CAAGATTCTCCCCATCTGCTTCTGCTCTTCCAGGCAGGGTTCATGTGGA---------AG
Macropus eugenii       CA-GATTCTCACTATCTGCTTCTGATCTTCCAGTCAGGGTTCAAGTGGA---------AG
Wallabia bicolor       NNNNNNNNNNNNNNNNNNNNNNNNNNNNNNNNNNNNNNNNNNNNNNNNN---------NN


Gallus gallus          CCTTGA---------AAAAGATAGCTTGGAAGGACCCCAAAACA
Monodelphis domestica  CTGTGA---------GAAAGAGAGTCAGGAAAGAGCCTTCAAAA
Macropus eugenii       CAGTGATGGCCCTGAGGAAGATAGTCAGGGAAGATGCCTCAAAG
Wallabia bicolor       NNNNNNNNNNNNNNNNNNNNNNNNNNNNNNNNNNNNNNNNNNNN
```

Pairwise SIM alignments to chicken sequence

```
Gallus gallus          GTGCAGGGCTATCTCATCCAGATCCTTGCAGACCAATCACTTCCCCCTGAGGTGCGAATG
Monodelphis domestica  TCAATAAACTCCAGTGGCTCCTAATTGCCCCTGGGATAAAAGGACAGCATCTCTGTTTGA
Macropus eugenii       ------------------------------------------------------------
Wallabia bicolor       ------------------------------------------------------------


Gallus gallus          ATGGCTTGTGCTGTTATCTTTGAGACAAGGCCTGCCCTTGCTTTGATAACGACTATAGCT
Monodelphis domestica  CATTTTTCTGCTTTCCTGTTTGAAGTACAGCCTCCTCCTTCTGTGATGACCACAGCAGCC
Macropus eugenii       --------TGCTTTGCTGTTTGAAGCTCAGCCTTCTCCTTCTGTGATGACCATGGTGGCC
Wallabia bicolor       ------TCTGCTTTGCTGTTTGAAGCTCAGCCTTCTCCTTCTGTGATGACCATGGTGGCC
                               **** *  * *****      **** * * * ** **** ** *     **


Gallus gallus          AACGTGGCAATGAAGGAGAGCAATATGCAAGTGGCCAGTTTTGTATATTCCCACATGAAG
Monodelphis domestica  GATATCATGCTAAAGGAGAAGAGCTTGCAAGTAGCCAACTTCATTTACTCCAACGTGAAG
Macropus eugenii       AATATCATGCTGAAGGAGAAAGACTTGCAAGTAGCCAGCTTCATTTACTCCTACATGAAG
Wallabia bicolor       AATATCATGCTGAAGGAGAAAGACTTGCAAGTAGCCAGCTTCATTTACTCCTACATGAAG
                        *  *     * *******      ******* ****  **  * ** *** ** *****


Gallus gallus          TCTTTGTCAAAGAGCAGATTGCCATTTATGTACAACAT
Monodelphis domestica  ACCATCTCCACAAATCACTTTCCAGACGGGGCTAATGT
Macropus eugenii       ACCATCTCCACAAATCACTTTCCAGATTGGGCTAACAT
Wallabia bicolor       ACCATCTCCACAAATCACTTTCCAGATTGGGCTAACAC
                        *  * ** *  *     ** ***     *   **
```

Pairwise SIM alignments to chicken sequence

Gallus gallus	ATCTTCAGCTTGTAACATTGCCCTTAAGCTCCTGTCCCCCAAACTGGACAGTATGAGCTA
Monodelphis domestica	GTCTGCAGGAAGCAACATTGCCATCAAATTCTTGAGTCCCAAACTGGAACACCTGACTTA
Macropus eugenii	NNNNNNNNNNNNNNNNNNNNNNNNNNNNNNNNNNNNNNNNNNNNNNNNNNNNNNNNNNNN
Wallabia bicolor	NNNNNNNNNNNNNNNNNNNNNNNNNNNNNNNNNNNNNNNNNNNNNNNNNNNNNNNNNNNN

Gallus gallus	TCGGTA-------------------CAGCAAGGTCATTCGAGCA-GACACTTACTTTG
Monodelphis domestica	CCACTATTTATTTCTCTTGCTACTTCAGCAAGTAAAATGGATCATGTGGGGTGTTTCC
Macropus eugenii	NNNNNNNNNNNNNNNNNNNNNNNNNNNNNNNNNNNNNNNNNNNNNNNNNNNNNNNNNN
Wallabia bicolor	NNNNNNNNNNNNNNNNNNNNNNNNNNNNNNNNNNNNNNNNNNNNNNNNNNNNNNNNNN

Pairwise SIM alignments to chicken sequence

Gallus gallus	ATAACTATAGAGTTGGTGCTACTGGAGAAATCTTTGTTGTGAACAGCCCAAGAACTATGT
Monodelphis domestica	GAGAGTAAAAGCCTGGCACGTTCATGAGATTCTTCATCTTATACAGTGTTGAGACTTTGT
Macropus eugenii	NNNNNNNNNNNNNNNNNNNNNNNNNNNNNNNNNNNNNNNNNNNNNNNNNNNNNNNNNNNN
Wallabia bicolor	NNNNNNNNNNNNNNNNNNNNNNNNNNNNNNNNNNNNNNNNNNNNNNNNNNNNNNNNNNNN

Gallus gallus	TCCCATCAGCAATAATTTCCAAATTGATGGCAAATTCTGCAGGTTCAGTGGCTGATCTGG
Monodelphis domestica	TCCCAACAGCCAAAACTTCGAGCCTGGGGGCACACTTTGCTGGGGCATCCATCAGTCTTG
Macropus eugenii	NNNNNNNNNNNNNNNNNNNNNNNNNNNNNNNNNNNNNNNNNNNNNNNNNNNNNNNNNNNN
Wallabia bicolor	NNNNNNNNNNNNNNNNNNNNNNNNNNNNNNNNNNNNNNNNNNNNNNNNNNNNNNNNNNNN

Gallus gallus	TAGAG
Monodelphis domestica	TGGAG
Macropus eugenii	NNNNN
Wallabia bicolor	NNNNN

Pairwise SIM alignments to chicken sequence

Gallus gallus	GTTGGCATCCGAGTGGAAGGCCTCGCAGATGTCATAATGAAAAGAAACATCCCATTTGCT
Monodelphis domestica	GCTGCTATCTAGGTGGAAGGTTTGACAGAAACTATGAGGAGAAAGAATACATAATTTGTG
Macropus eugenii	NNNNNNNNNNNNNNNNNNNNNNNNNNNNNNNNNNNNNNNNNNNNNNNNNNNNNNNNNNNN
Wallabia bicolor	NNNNNNNNNNNNNNNNNNNNNNNNNNNNNNNNNNNNNNNNNNNNNNNNNNNNNNNNNNNN

Gallus gallus	GAATATCCCACATACAAGCAGATAAAGGAGCTTGGAAAAGCT
Monodelphis domestica	GAATCTCCCTCACATAAGAAGATAAAGGAACTTGTAAAGGCA
Macropus eugenii	NNNNNNNNNNNNNNNNNNNNNNNNNNNNNNNNNNNNNNNNNN
Wallabia bicolor	NNNNNNNNNNNNNNNNNNNNNNNNNNNNNNNNNNNNNNNNNN

Pairwise SIM alignments to chicken sequence

Gallus gallus	CTGCAGGGATGGAAAGAGCTGCC
Monodelphis domestica	CTGCAGGGACAGAGGGAGCTACC
Macropus eugenii	NNNNNNNNNNNNNNNNNNNNNNN
Wallabia bicolor	NNNNNNNNNNNNNNNNNNNNNNN

Pairwise SIM alignments to chicken sequence

```
Gallus gallus          TACAGTTGCAACGATGGGTGTCAACACAGAATACTTTCAACATGCTGTTGAAATT-----
Monodelphis domestica  TACACTGGCAGCAATGGCCATCAACACTGCTGTCTTTCAG------ATTGACATT-----
Macropus eugenii       CTCTGTGGCAGTAATGACCATCAACACCCCTGTCTTCCAG------TCTGGCATTGAGCT
Wallabia bicolor       CTCTGTGGCAGCAATGACCATCAACACCCCTGTCTTCCAG------TCTGGCATYgagct
                         *  * ***   ***    *******      *** **         **  **


Gallus gallus          -------------CAAGGCGAGGTCCAGACAAGAATGCCAATGAAGTTTGATGCCAAGAT
Monodelphis domestica  -------------CAAG--------CAGATCAC----TCAATGGAATTGGTCACCAAGTT
Macropus eugenii       CCAGGCAAAACTCCAGG--------CAACTTAC----CCAATGAAATTGGACATGAAATT
Wallabia bicolor       ccaggcgaaactcCAGG--------CAACTTAC----CCAATGAAATTGGACATGAAATT
                                    ** *        **    *      ***** * ** *     **  *


Gallus gallus          AGATGTGAAATTGAAAAACCTTAAGATTGAAACGAACCCATGCCGTGAGGAAACTGAGAT
Monodelphis domestica  AGACCGAGAAGAGAAAAGCTTTAAGTATGAAATCTTCCCATTAGACCAAAAGACTAAGCT
Macropus eugenii       GGACCTAAAAGAGAAACACTTTAAGTATGAAACCTTTCCATGGCCCCCAGAGTCGGAGCT
Wallabia bicolor       GGACCTAAAAGAGAAACACTTTAAGTATGAAACCTTTCCATGGCCCCCAGAGTCGGAGCT
                        **     **  ****  * *****  *****     ****         *  *  **  *


Gallus gallus          AGTGGT
Monodelphis domestica  CCTGAT
Macropus eugenii       CCTGAT
Wallabia bicolor       CCTGAT
                         ** *
```

Pairwise SIM alignments to chicken sequence

```
Gallus gallus          ACATAAGGCTTTTGCTGTATCAAGGAACATAGGAGAACTAGGTGTTGAAAAGAGGACCTC
Monodelphis domestica  TGCCAAGGCATTTGCTGTATCTAGAAAGAGTCAGGAGCCAGAAACCGAAGAGAAGATTCC
Macropus eugenii       TGCCAAGGCATTTGCTATATCAAGAAAGAGTGGGGAGCCAGAAATGGAAAAGAAGATTCC
Wallabia bicolor       TGCCAAGGCATTTGCTGTACCAAGAAAGAGTGGGGAGCCAGAAATGGAAAAGAAGATTCC
                           ***** ****** ** * ** ** *     ** * **     *** *** **   *


Gallus gallus          AATTCTGCCGGAAGATGCTCCATTAGATGTTACAGAAGAACCTTTCCAAACATCAGAGAG
Monodelphis domestica  TTTAGTTCTTGAGAAAACCAGATCTGAGATCTCCAAGGAGCAATTTGGTTCAACAGATAG
Macropus eugenii       TCTAGTTCCTGAGGAAGCAGGATCTGAGATCTCCAAGGGGCAAGTTGGTTCTGCAGAGAT
Wallabia bicolor       TCTAGTTCCTGAGGAAGCAGGATCTGAGATCTCCAAGGGGCAAGTTGGTTCCGCAGAGAT
                         *  * *  **  *  *   **  **  *  *  * *  *   *     *  **** *


Gallus gallus          AGCTTCCAGGG
Monodelphis domestica  GACTTTGAAGG
Macropus eugenii       GATTTTTAAGG
Wallabia bicolor       GATTTTTAAGG
                          **  * **
```

Pairwise SIM alignments to chicken sequence

```
Gallus gallus          CCTAAGTTTCTGGGTGACATCAAAACCCCAGTGTTAGCTGCTTTTCTCCATGGCATTAGT
Monodelphis domestica  ATGAAGTTCTTGGAGGACAACTATCCATCCATGCTAACTGCAATATTTCAGGTAGAATGC
Macropus eugenii       ATGATGTTCTCGGAAAGCAATCATCCACCCATAATGACTGGAATATTTAAGGTAGAATAC
Wallabia bicolor       NNNNNGTTCTCGGAGAGCAATCATCCACCCATAATGACTGGAATATTTAAGGTAGAATAC
                            ***   **    **   *  *  *  *  *  ***   *  *  * *
```

```
Gallus gallus          AACAATAAGAAGACAGGAGGCCTCCAGCTTGTGGTATATGCTGATACCGACTCGGTCAGG
Monodelphis domestica  AGAGACCATAAGCCTGATGACTTCCAGTTTTCTATATTCACAGATCAACACTCCTCCAAA
Macropus eugenii       AGTGACCATAAGCCTGACAGCTTTCAGTTTGCTATCTTCACAAATAAGCACTCCTCCAAA
Wallabia bicolor       AGTGACCATAAGCCTGACAGCTTTCAGTTTGCTATCTTCACAAATAAGCACTCCTCCAAA
                       *   *  * *** * *    * * *** **    * *   *  **    ****   **

Gallus gallus          CCGCGGGTGCAGGTATTTG-TGACAAACCTCACAGATTCGAGCAAGTGGAAGCTCTGCGC
Monodelphis domestica  GCTGGGATCCAGTTATGGGGTCATGAATTTCACCAGAAGTAGCCAGTGGGAACCTTGTGT
Macropus eugenii       GCTGGCATCCAGTTACGGG-TCACTAATTTCACCAAAAGTAGCCAATGGAAAGCTTGTGT
Wallabia bicolor       GCTGGCATCCAGTTACGGG-TTACTAATTTCACCAAAAGTAGCCAATGGAAAGCTTGTGT
                        *  *  * *** **   * * *  **  ****       *** * *** *    ** *

Gallus gallus          AGAT---GCTTCGGTCCGCAATGCTCACAAGGCAGTG
Monodelphis domestica  TGAA---ATCTTG---CAGAATTTTCATATGGCAAAG
Macropus eugenii       TGATGTCATCTTG---GAGACTCTTTTCTTGGCAAAG
Wallabia bicolor       TGATGTCATCTTG---GAGACTCTTTTCTTGGCAAAG
                        **       * *      * *  *     ****  *
```

Pairwise SIM alignments to chicken sequence

```
Gallus gallus          GCCTACGTGAAATGGGGCTGGGACTGCCGGGACTACAAGGTTTCTACTGAGCTGGTAACT
Monodelphis domestica  ACATCCCTGAAATGGGATCAGAATTGCCAGGACTGTCAAATTTGGGAAGAGATTGTGACT
Macropus eugenii       GCATCCCTGAAGTGGGGTCAGAATTGCCAGGAGTATCAAGTTTCAGGGGAGATTGTCACT
Wallabia bicolor       GCATCCCTGAAGTGGGGTCAGAATTGCCAGGAGTATCAAGTTTCAGGGGAGATTGTCACT
                        * * * **** ****    * * **** *** *   *  ***     *** * ** ***

Gallus gallus          GGGCGGTTTGCTGGGCACCCTGCTGCACAAGTGAAGCTGGAGTGGCCCAAGGTTCCTTCG
Monodelphis domestica  GGGCAGTTTACTAATCACTCCATTGTGCAA-TGAAGGTTGCATGATCAAAAGCCTCAGCA
Macropus eugenii       GGGCAGTTTGTTAATCACTCCATTGTGCAAATGGAGGTTGCTTGGCCAGAAGTCTCAGCA
Wallabia bicolor       GGGCAGTTTGTTAATCACTCCATTGTGCAAATGGAGGTTGCTTGGCCAGAAGTCTCAGCA
                       **** ****  *   *** *   **  *** ** ** * *  **  *  * *   *  *

Gallus gallus          AATGTCAGATCAGTGGTTGAATG
Monodelphis domestica  AAGGTCACAGAAATGGGAAGATG
Macropus eugenii       GAAGTCATAGAAATGGGAAAATG
Wallabia bicolor       GAAGTCATAGAAATGGGAAAATG
                        * **** *  * ***    ***
```

Pairwise SIM alignments to chicken sequence

```
Gallus gallus          GTTTTACGAGTTTGTCCCTGGGGCTGCATTTATGCTGGGTTTCTCTGAGAGAATGGACAA
Monodelphis domestica  GTTTTATACATTCATTCCTGGACTTCTTTCTAGCCTGGGATTTTCTGAGAAAGGCTGCAA
Macropus eugenii       GTTTTATACATTCATTCCTGGGTTTTTCTCTAGTCTGGGATTCTCTGAAAGAGCCTGGAA
Wallabia bicolor       NNNNNNNNNNNNNNNNNNNNNNNNNNNNNNNNNNNNNNNNNNNNNNNNNNNNNNNNNNNN

Gallus gallus          GAATCCTTCTCGACAAGCCAGGATGGTTGTGGCTCTAACTTCTC---------------C
Monodelphis domestica  AAAACCTCTTTGGAAGGCTACCTTGGAAATAGCACCCCCATCTC---------------C
Macropus eugenii       AAATCCCCTTCAGAAAACCAGCTTGGTGATGTCACTTACACCTCGCAGAAGTGTGGACGT
Wallabia bicolor       NNNNNNNNNNNNNNNNNNNNNNNNNNNNNNNNNNNNNNNNNNNNNNNNNNNNNNNNNNNN
```

Pairwise SIM alignments to chicken sequence

```
Gallus gallus          CTCGCTGCTCAGTTTCGTACAACAAGATCAAAACCTTTAATGAAGTCAAGTTCAACTACT
Monodelphis domestica  CTGACAATTCAGTCAATAAAGAGAAGATCAGCACCTTCAGTGGCATTAAGTATAGTTATC
Macropus eugenii       CTGACTGCTCAGTTAATCAAGAGAAGATCACCACCTTCAGTGGAATCCAGTACAGCTATC
Wallabia bicolor       NNNNNNNNNNNNNNNNNNNNNNNNNNNNNNNNNNNNNNNNNNNNNNNNNNNNNNNNNNNN


Gallus gallus          CGATGCCCGCAAACTGCTATCACATCTTGGTTCAGGATTGCAGCT----CTGAACTTAAG
Monodelphis domestica  CCCTGCTTGCCAGCTGTTTCCACATCCTAACTCAGGAT----GCT----CTGAACTGAGG
Macropus eugenii       CCTTGCTTGCCAACTGCTTTCACATCCTAACCCAGGAT----GCTAGCTCTGAACTGAGG
Wallabia bicolor       NNNNNNNNNNNNNNNNNNNNNNNNNNNNNNNNNNNNNNNNNNNNNNNNNNNNNNNNNNNN


Gallus gallus          TTCCTGGTGATGATGAAAAGTGCTGGAGAA------GCTACAAACCTTAAAGCCATCAAC
Monodelphis domestica  TTCCTCATCCTGATAAAGAC------AGAT------TCTATTGATCTATAGGCTATCAGT
Macropus eugenii       TTCCTGGTGTTGATAAAAAC------AGCTGAAGAATCTGCTGACCAAAGGGCCATCAAT
Wallabia bicolor       NNNNNNNNNNNNNNNNNNNNNNNNNNNNNNNNNNNNNNNNNNNNNNNNNNNNNNNNNNNN


Gallus gallus          ATCAAGATTGGCAGTCA
Monodelphis domestica  GTCAAACTGACTGATCA
Macropus eugenii       GTCAAACGGGCCAATCA
Wallabia bicolor       NNNNNNNNNNNNNNNNN
```

Pairwise SIM alignments to chicken sequence

```
Gallus gallus          TGAAATTGATATGCATCCTGTGAATGGACAGGTGAAACTGCTGGTAGATGGGGCTGAGAG
Monodelphis domestica  TGACATTGGCATGTGTCCTGGTCAAGGACA------ACTTCAGGGGGATTGCAAAGAGAC
Macropus eugenii       TGACATTGACATGTATCTTGCTAAAGGAAACCTTTGGCTTCAGGTGGACAGCAAAGAGAT
Wallabia bicolor       NNNNNNNNNNNNNNNNNNNNNNNNNNNNNNNNNNNNNNNNNNNNNNNNNNNNNNNNNNNN


Gallus gallus          CCC
Monodelphis domestica  CCC
Macropus eugenii       TCC
Wallabia bicolor       NNN
```

Pairwise SIM alignments to chicken sequence

```
Gallus gallus          GTGCTTCTCTGTGGATTCACAATGAAAACCAAGGGTTTGCACTTGCTGCCCCAGGCCATG
Monodelphis domestica  TCACTTG--TGTCCATCAAGAATGAAAGTCAAGGATTCTTTCTGTATGTGCCAGAGCATG
Macropus eugenii       TCATTTG--TGTCTATCAAGAATGAAAGTCAAGGATTCTCTCTTTCTGCACCAGAGCATG
Wallabia bicolor       NNNNNNNNNNNNNNNNNNNNNNNNNNNNNNNNNNNNNNNNNNNNNNNNNNNNNNNNNNNN


Gallus gallus          GTATCGATAAATTGTACTTCGATGGAAAAACAATCAC--------G
Monodelphis domestica  GCATTGAAAATCTTTAT---GATGGGAAAACATGCAA--------T
Macropus eugenii       GTATTGAAAAACTTTATTATGATGGGAAAACATGCAAGGTATCTTT
Wallabia bicolor       NNNNNNNNNNNNNNNNNNNNNNNNNNNNNNNNNNNNNNNNNNNNNN
```

Pairwise SIM alignments to chicken sequence

Gallus gallus	ATTCAAGTTCCTTTATGGATGGCAGGGAAAACATGTGGAATCTGTGGAAAATATGATGCA
Monodelphis domestica	GTTCACATTTCTTTCTGGATGATTGGAAAAACACATCATCTGCATAGACAAGCAAATGCA
Macropus eugenii	GTTCATATTTCTTTCTGGATGATTGGAAAAACACTTGGCCTCTGTGGACAAGCAAATGCA
Wallabia bicolor	NNNNNNNNNNNNNNNNNNNNNNNNNNNNNNNNNNNNNNNNNNNNNNNNNNNNNNNNNNNN

Gallus gallus	GAATGCGAACAGGAGTATCGGATGCCCAATGGATATCTAGCTAAAAATGCCGTGAGCTTT
Monodelphis domestica	GAAACCACCCAGGAGTTTCAGATGCCCGGTGGCTACATGGCTAAAGATGCTATGAGTGCT
Macropus eugenii	GAAACCATTCAAGAATTCCAGATGCCCAGTGGCTACATAGCTAAAGATGCTGTGAGCTTT
Wallabia bicolor	NNNNNNNNNNNNNNNNNNNNNNNNNNNNNNNNNNNNNNNNNNNNNNNNNNNNNNNNNNNN

Gallus gallus	GGTCATTCTTGGATCTTGGAAGAAGCGCCCTGTAGAGGAG
Monodelphis domestica	GCTCAATCTTGGGTTCTGACAGATGGCTCTTGCTCCAGGG
Macropus eugenii	GCTTGATCTTGGGTTCTGACAGATGACTCCTGCTCCAGAG
Wallabia bicolor	NNNNNNNNNNNNNNNNNNNNNNNNNNNNNNNNNNNNNNNN

Pairwise SIM alignments to chicken sequence

Gallus gallus	CTTGTAAACTGCATCGTTCATTCGTGAAGCTTGAGAAGACGGTTCAGCTGGCGGGTGTTG
Monodelphis domestica	CATGTTCTTTTCAACACCAGTTCATGAAACTTGAGGAAATAATTTTCCTTCAAGGAGAAC
Macropus eugenii	CTTGTTCCTTTCAACACCAGTTCATGAGACTTGAGAAAACAATTTACCTTCAAGGAGAAA
Wallabia bicolor	NNNNNNNNNNNNNNNNNNNNNNNNNNNNNNNNNNNNNNNNNNNNNNNNNNNNNNNNNNNN

Gallus gallus	ATTCCAAGTGCTACTCTACAGAGCCTGTGCTGCGCTGTGCAAAGG----GATGCTCTGCT
Monodelphis domestica	ATTCTTCTTGCTTTTCAGCACAGCCAGTGTTGGGGTGTCTGAAGGACTAGAAAATGGGGT
Macropus eugenii	ATTCCTCATGCTCATCCGTGCAGCCAGTGTTGAGGTGTTGGAAGGGCTGCAGAGTGGGCC
Wallabia bicolor	NNNNNNNNNNNNNNNNNNNNNNNNNNNNNNNNNNNNNNNNNNNNNNNNNNNNNNNNNNNN

Gallus gallus	ACCAAGACAACTCCAGT-AACTGTTGGCTTCCACTGCCTCCCAGCTG
Monodelphis domestica	AAACCATCAGCACCCTT-A----TTGGCTTTTATTAACTTCCAATTG
Macropus eugenii	AAACCATCACCACCCTTTG----TTGACTTTCATTGCCTTCCAGCTG
Wallabia bicolor	NNNNNNNNNNNNNNNNNNNNNNNNNNNNNNNNNNNNNNNNNNNNNNN

Pairwise SIM alignments to chicken sequence

Gallus gallus	ATTCAGCTAACAGCCTGACTGACAAACAGATGAAGTACGACCAGAAGTCAGAAGACATGC
Monodelphis domestica	ATTCTGATGCCCACTCTGCCACTGCCCTGACAAGGTTAGACCTGAAGTCCCAAGATATGG
Macropus eugenii	ATTCTGATGCCCATTTTGCCATGGCACCATCAAGATTGGAGCTAAAGTCTGAGGATGTGG
Wallabia bicolor	NNNNNNNNNNNNNNNNNNNNNNNNNNNNNNNNNNNNNNNNNNNNNNNNNNNNNNNNNNNN

Gallus gallus	AGGA
Monodelphis domestica	AGGA
Macropus eugenii	AGGA
Wallabia bicolor	NNNN

c. *VIT3*

Pairwise SIM alignments to chicken sequence

```
Gallus gallus          TTATTTTTCAATAGAGCCTGTTTTTAGTACCGGCAAGACCTACCTGTATGACTACAAGGG
Monodelphis domestica  CCCTATCCCAGTAAAGG-----TTCAACTGCAACAAGATCTATATGTTTGTCTATAAAAA
Macropus eugenii       NNNNNNNNNNNNNNNNNNNNNNNNNNNNNNNNNNNNNNNNNNNNNNNNNNNNNNNNNNNN
Wallabia bicolor       NNNNNNNNNNNNNNNNNNNNNNNNNNNNNNNNNNNNNNNNNNNNNNNNNNNNNNNNNNNN


Gallus gallus          CCTCATCCTGCATGGGCTGCCAGGCAAGGGGTTGGCAGCAGCGGG
Monodelphis domestica  GATGATTTTCAGTGAGCGGCCAGAAAAAAGGTTTGGCTCATTGGG
Macropus eugenii       NNNNNNNNNNNNNNNNNNNNNNNNNNNNNNNNNNNNNNNNNNNNN
Wallabia bicolor       NNNNNNNNNNNNNNNNNNNNNNNNNNNNNNNNNNNNNNNNNNNNN
```

Pairwise SIM alignments to chicken sequence

```
Gallus gallus          CCACTGCATGACCTTGCTGCTGAAGCTTCAAGCAGA----AGCCATGTTGAAGACATGGC
Monodelphis domestica  TTATTTCTTGACTTTGTTACCATGGCTTTTAGCAGATAGAGGTCATAAGGAAGAAATGAC
Macropus eugenii       CTGT-------CTTTCTTACTATGGCTTTTAGCAGATATAGGTCat---------atGAC
Wallabia bicolor       CTGT-------CTTTCTTACTATGGCTTTTAGCAGATATAGGTCat---------atGAC
                                  * **  * *    ****  ******     * ***         *** *


Gallus gallus          CTTGGCCCTGAAAGCTATAGGCAATGCAGGAGAGCCAGCCAGCATCAAACGCATCCTGAA
Monodelphis domestica  TACAGCCCTGAAGGATATTGGGAATGTGGGCCAGTTAGAAAGTCATAAACACATTCATAA
Macropus eugenii       CACAACCCTGAAGGTTATtGCTAATGTAGKCCAgYYSGAAAGCYTTAAAACAATCCAGAA
Wallabia bicolor       CACAACCCTGAAGGTTATTGCTAACGTAGGCCAGTCAGAAAGCTTTAAACCAATTCAGAA
                            ******* * *** *  ** *  *   **   *  **    ***   ** *  **


Gallus gallus          GTTTTTGCCAACATTTTCACTTGCTGCCGCGGCATTACCAAGCAGAATTCATACT-GATG
Monodelphis domestica  GTTCCTGACAGTATTTTAATTTGCTGCTTCTGATCTTCCAGTTTTAATTCAGGCAAGAAA
Macropus eugenii       GTTCCTGCCAATATTTTAATTTGCTGCTTCTGATCTTCCAGTTATAATTCAGGCA-GAAA
Wallabia bicolor       GTTCCTGCCAATATTTTAATTTGCTGCTTCTGATCTTCCAGTTATAATTCAGGCA-GAAA
                       ***  ** **  ***** * *******  * *   * ***     ******  *  **


Gallus gallus          CCGTGCTGTCCTTGAG---GAAAATAGCCAGAAAAGACCCTGCAAAA
Monodelphis domestica  CTATTATGGCTCCAAGTTGGAAAAGAAGTTGCAAATATCTCTCAAAA
Macropus eugenii       CTATTATACCTCTAAGATGGGAAACAGG----------CCCTCAAAA
Wallabia bicolor       CTATTATACCTCTAAGTTGGGAAACAGG----------CCCTCAAAA
                       *  *  *  *    **   *  *** *            *   *****
```

Pairwise SIM alignments to chicken sequence

```
Gallus gallus          GTAAGGGGGATCGCTCTCCAAATTTTTATGGACAGCACACTTGCTCCTAATGTGCGCATG
Monodelphis domestica  GTACAGGAAGATTTGCTTCAGATTTACATGGAGCATTCAATTGATCCCAGTGTGAGAATT
Macropus eugenii       NNNNNNNNNNNNNNNNNNNNNNNNNNNNNNNNNNNNNNNNNNNNNNNNNNNNNNNNNNNN
Wallabia bicolor       NNNNNNNNNNNNNNNNNNNNNNNNNNNNNNNNNNNNNNNNNNNNNNNNNNNNNNNNNNNN


Gallus gallus          ACGGCTTGCATTGTCCTCTTTGAAACAA----AGCCTTCTCTTCCTATCGTGGTAGCCAT
Monodelphis domestica  ACAGCTTTTGTGGTCCTTTTGGATGCTGTTTGGATTTCTTTGACCATGGGGATAGGAGA
Macropus eugenii       NNNNNNNNNNNNNNNNNNNNNNNNNNNNNNNNNNNNNNNNNNNNNNNNNNNNNNNNNNNN
Wallabia bicolor       NNNNNNNNNNNNNNNNNNNNNNNNNNNNNNNNNNNNNNNNNNNNNNNNNNNNNNNNNNNN
```

```
Gallus gallus          GGCCAGCTCGCTGCTGACGGAGACCAGCTTACAAGTGACCAGTTTCATGTATTCGCACAT
Monodelphis domestica  TACTA------TGTTAAAGGAGACCAGCTTACAAGGGGTGAGTTTCACTTAAGCTCATTT
Macropus eugenii       NNNNNNNNNNNNNNNNNNNNNNNNNNNNNNNNNNNNNNNNNNNNNNNNNNNNNNNNNNNN
Wallabia bicolor       NNNNNNNNNNNNNNNNNNNNNNNNNNNNNNNNNNNNNNNNNNNNNNNNNNNNNNNNNNNN
```

```
Gallus gallus          GAAGGCTTTGGCAGTAGGCAGGATCCCACAGCTCTACAATCT
Monodelphis domestica  GACATCTTTTTCCAAAAGCAAGATTCTACTCCTGGTCAATAT
Macropus eugenii       NNNNNNNNNNNNNNNNNNNNNNNNNNNNNNNNNNNNNNNNNN
Wallabia bicolor       NNNNNNNNNNNNNNNNNNNNNNNNNNNNNNNNNNNNNNNNNN
```

Pairwise SIM alignments to chicken sequence

```
Gallus gallus          TCCGGTCACAAAGCCTAACCAGTATCATCAGGAAACAGAATATTCCCTTTGCTGAATACG
Monodelphis domestica  TCCAAGCTTAAGGTTTAATAGATATCCTCTGGAAACACATCAAGACATTTGCTGACTCTT
Macropus eugenii       NNNNNNNNNNNNNNNNNNNNNNNNNNNNNNNNNNNNNNNNNNNNNNNNNNNNNNNNNNNN
Wallabia bicolor       NNNNNNNNNNNNNNNNNNNNNNNNNNNNNNNNNNNNNNNNNNNNNNNNNNNNNNNNNNNN
```

```
Gallus gallus          ATACATACAAGACACTGAAGGAACTTGGTAAAAAG
Monodelphis domestica  TTATGAGGAGAAACATAAAAGAGCTTGTGAAAACA
Macropus eugenii       NNNNNNNNNNNNNNNNNNNNNNNNNNNNNNNNNNN
Wallabia bicolor       NNNNNNNNNNNNNNNNNNNNNNNNNNNNNNNNNNN
```

Pairwise SIM alignments to chicken sequence

```
Gallus gallus          CTTCTGGGGTGGAAAGAATTGCCTCCCGAAGATCCTCTGCTGTCTGCTTACATCAAAGTG
Monodelphis domestica  TTAGGTAGGCAGAAAGGATTACCTGCTGGAAAACTTCCGATTTCAATGGATTTCAAAATA
Macropus eugenii       NNNNNNNNNNNNNNNNNNNNNNNNNNNNNNNNNNNNNNNNNNNNNNNNNNNNNNNNNNNN
Wallabia bicolor       NNNNNNNNNNNNNNNNNNNNNNNNNNNNNNNNNNNNNNNNNNNNNNNNNNNNNNNNNNNN
```

```
Gallus gallus          TTGGGCCAGGAGATTGCCTTCGTTGACATTGACA
Monodelphis domestica  TTTGGTCAAGAGTCATTCTTCTTAAATATCAACA
Macropus eugenii       NNNNNNNNNNNNNNNNNNNNNNNNNNNNNNNNNN
Wallabia bicolor       NNNNNNNNNNNNNNNNNNNNNNNNNNNNNNNNNN
```

Pairwise SIM alignments to chicken sequence

```
Gallus gallus          AGTATATATTCATATGATTGCTATGATGGGTACTAATAC-ACAGTACCTTCAGAGTGGTC
Monodelphis domestica  TGTTTGTTGGCACACAATGACAACCATGTTGATCGATACCACCAATCTCTCAGTCTGGTA
Macropus eugenii       TGTCTATTGCAACACAATGGCAGCCATATTGATCG---CACTCCATCTTCCAAACTGGTA
Wallabia bicolor       NNNNNNNNNNNNNNNNNNNNNAGCCATATTGATCG---CACTCCATCTTCCAAACTGGTA
                                                ** ** *      *       *   **   ****
```

```
Gallus gallus          TTGAACTTCATGCAGAGTTCAGTGCCA-ACACTACAATGAAATTTGATGCCAGGATAAAT
Monodelphis domestica  TTCAGTTACA-GTAGAAGGCCTTGCCTCATTCCCTGCTGAAATTTGATGCCAAGATCAAC
Macropus eugenii       TTCAGT-ATG-TTAAGAAGCCTTG--TCAT----------AATTCGATGTCCAAATCAGG
Wallabia bicolor       TTCAGTTAC--GTAGGAAGCCTTG--TCAT----------AATTCGATGTCATAATCAGG
                       ** *         *     *  **    *           **** **** *   ** *
```

```
Gallus gallus          ATGAAAGAGAAGAATG-----TGAAGATTGAAACC--------------------CTTCC
Monodelphis domestica  TGGAAAGAAAAAAAAAACCCTTCAAATTTGAAAGT--------------------CTGCT
Macropus eugenii       TAGAAAGAGAAAAAAAAAATCTTNNNNNNNNNNNNNNNNNNNNNNNNNNNNNNNNNNNNN
Wallabia bicolor       TAGAAAGAAAAAAA--ATCTTTCAGATTTGAAACTAACTGCCTCTCTTCAGGAAGTTGCC
                          ****** ** **       *


Gallus gallus          CTG
Monodelphis domestica  CTG
Macropus eugenii       NNN
Wallabia bicolor       CTG
```

Pairwise SIM alignments to chicken sequence

```
Gallus gallus          AC-TTAAGCCTATGTTCCTGGGAGACA-GCAAGCCACCAGTACTGGCTGCAGTTCTTCGT
Monodelphis domestica  TCCTTCAGCTTAGTTTTCTGGGAGTTAAGTAAGGTTCTGGTGGTGGCTGCCATCATGTGG
Macropus eugenii       TCCTTCAGCTTAATTTCTTGGGAGATG-GTGAAATTCCGGCATTGGCTGCCATCATGGGG
Wallabia bicolor       NNNNNNNNNNNNNNNNNNNNNNNNNNNNNNNNNNNNNNNNNNNNNNNNNNNNNNNNNNNN


Gallus gallus          GCCATCAGAAG----AAATGAGCAGCCGACAGGATACCAGC-TTGTACTTTACACTGACA
Monodelphis domestica  TCTTCTAGAAGTGTCAAAGGAACAA--GGCTGGCTACCAGC-AGGTATTGTATACAGACA
Macropus eugenii       CCTT-TGGAAGTGACAAACAGACAA--GGCTGGCTACCAGCTAAGTTATACATTCAGATG
Wallabia bicolor       NNNNNNNNNNNNNNNNNNNNNNNNNNNNNNNNNNNNNNNNNNNNNNNNNNNNNNNNNNNN


Gallus gallus          GACAGGCCTCCAGACTGAGGGTGCAGGTGTTTGTTTCA-AGCATCACAGAATCAGATAGG
Monodelphis domestica  TGCAGATACTCAAACTCA------AGGCCTTTTTTTAATAGCTTCTTGAACTCCAGCAAG
Macropus eugenii       TTCAGA--CTCAGGGTAC------AAGCTTTTTATCAACAGTCTCTTGAACTTCAGCAAG
Wallabia bicolor       NNNNNNNNNNNNNNNNNNNNNNNNNNNNNNNNNNNNNNNNNNNNNNNNNNNNNNNNNNNN


Gallus gallus          TGGAAACTCTGTGCTGATGCTTCAGTAGTAAATTCCCATAAAGCATCG
Monodelphis domestica  TGGAAGGTCTCCACTGACATCTCAATGGTGAATTCTCAC------TTG
Macropus eugenii       TACAACG----------------------------------------G
Wallabia bicolor       NNNNNNNNNNNNNNNNNNNNNNNNNNNNNNNNNNNNNNNNNNNNNNNN
```

Pairwise SIM alignments to chicken sequence

```
Gallus gallus          GGTACCCTTAAATGGGGTAAAGATTGCCAGGACTATCAGGTTGCTACTCAGATT-GCAAC
Monodelphis domestica  GGTTATTTCAAATGGGGTCAGAAATA--AAGAGTACAAACCTGTTGCTATGTTA-GTGAC
Macropus eugenii       AGTG---------GGGATTGTAAATG--AAAA-CACAAACTTGTTATTATAACAAGCGAC
Wallabia bicolor       NNNNNNNNNNNNNNNNNNNNNNNNNNNNNNNNNNNNNNNNNNNNNNNNNNNNNNNNNNNN


Gallus gallus          AGGGCAATTTGCTGCACACCCGGCTATACAGGTGAAGCTGGAGTGGTCAGAAGTGCCTTC
Monodelphis domestica  TAGTCACTTTTCTAATTATCCAGCAGTGTAAATGAAGTTAGTATGGCCTCAAGTCCTTTC
Macropus eugenii       TATTCACTTTTATAACCATTTAGCAATACACATGAAGTCAGTGTGGCCTCAAGTCCTTTC
Wallabia bicolor       NNNNNNNNNNNNNNNNNNNNNNNNNNNNNNNNNNNNNNNNNNNNNNNNNNNNNNNNNNNN


Gallus gallus          AAGTGTCCGAAAAACTGCCA--GATG
Monodelphis domestica  TAAACTC-AAAGCAGTGGCA--G-TG
Macropus eugenii       CAAAATG-AAAACAGTGGCAAAG-TG
Wallabia bicolor       NNNNNNNNNNNNNNNNNNNNNNNNNN
```

Pairwise SIM alignments to chicken sequence

```
Gallus gallus          GTTCTACACATATCTTCCAGGAGCTGCGTATATGCTTGGCTACTCCCAGAAGCAGCAGCG
Monodelphis domestica  GTTTTATGGATATGCTTCAAGAGTTGCATATATGTGAAGATTCTCTGAAAAGAAATGGAA
Macropus eugenii       GTTTTATGGATATGCTCCAAGAGTTGCATATATGTGAAGCTTCTCTGAAAAGAAATGGAA
Wallabia bicolor       GTTTTATGGATATGCTCCAAGAGTTGCATATATGTGAAGCTTCTCTGAAAAGAAATGGAA
                       *** **   ****  * ** *** *** ******    * * ***  * *** *   *
```

```
Gallus gallus          TGGTCCTTCTCACCAGG
Monodelphis domestica  AAATCCTTCTCAACAGG
Macropus eugenii       TAATCTTTCTCAACAGG
Wallabia bicolor       TAATCTTTCTCAACAGG
                          ** ****** ****
```

Pairwise SIM alignments to chicken sequence

```
Gallus gallus          C--AGCTCGTTGTTCAGTTTCACAAAATATGATAACAACCTTCAATGGAGTTGAGTTTAA
Monodelphis domestica  C--AGCTCACTCTTTGGCTGAACA--------TTAAAATGCTCAACAGAATTAAGTATAA
Macropus eugenii       CTCAGCTTACTCTTTGACCAAACA--------CTAAAATGTTCAAAAGGATTAAATATAA
Wallabia bicolor       NNNNNNNNNNNNNNNNNNNNNNNN--------NNNNNNNNNNNNNNNNNNNNNNNNNNNN
```

```
Gallus gallus          CTATTCAATGCCTGCAAATTGCTACCACATCTTGGCACAGGATTGTAGTCCAGAACTGAA
Monodelphis domestica  CTACGAGATGTTTGCAAATTGCTACCATATCCTAGATTCGGACCAAAGCCAAGATCTGAG
Macropus eugenii       CTATGAGATGTTTGCAAATTGCTACCACATCCTAGCCTAGGACAGCAGCCAAGATCTGAG
Wallabia bicolor       NNNNNNNNNNNNNNNNNNNNNNNNNNNNNNNNNNNNNNNNNNNNNNNNNNNNNNNNNNNN
```

```
Gallus gallus          GTTCCTGGTAATGATGAAAAGACTTGGAGAATCTGCTGATCTCACAGCAATAAGTGTCAG
Monodelphis domestica  ATTCCTTG-GATGATAAAGAAAGCTG-AAAGCCTACCAACCAAAAAATCTTCAATCTCAA
Macropus eugenii       GTTCCTGGTGATGATAAAGAAGGCTG-AAGGTCTGCCAACCAAAAAATTATCAATGTCAG
Wallabia bicolor       NNNNNNNNNNNNNNNNNNNNNNNNNNNNNNNNNNNNNNNNNNNNNNNNNNTCAATGTCAG
                                                                         * * * ***
```

```
Gallus gallus          ACTTGCCAGCCA
Monodelphis domestica  ACTTGGCTCTCA
Macropus eugenii       ACTTAGTTGTCA
Wallabia bicolor       ACTTAGTTGTCA
                       ****      **
```

Pairwise SIM alignments to chicken sequence

```
Gallus gallus          TGAGGTTGACATGTATGTTTCCAATGGACTAATCC---AGCTGAAGATTAATGGTGTTCA
Monodelphis domestica  TGACATTGACATGTATACT---AAAGGAAAATGCCCTAAACTGAAGATTAATGG-GTTAG
Macropus eugenii       TGACATTGTCATGTATCCT---AAAGGAGAATGCCTTAGAGTGAAGATTAATGA-GTTAG
Wallabia bicolor       TGACATTGTCATGTATCCT---AAAGGAGAATGCCTTAGAGTGAAGATTAATGA-GTTAG
                       ***  *** *******  *   ** ***  *  **      ************  ***
```

```
Gallus gallus          AACCCCAAC----AGACGTTCCATACACATCTAAGTCTG
Monodelphis domestica  AAACTTAGCTGTAAAAACTCTCATGTCAGTCTACTTTAG
Macropus eugenii       AAACTCAGCTGGAAAAACT-----ATCAGTGTGGTTTGG
Wallabia bicolor       AAACTCAGCTGGAAAAACTCCTAT--CAGTCTGGTTTGG
                       ** *  * *    * *  *          * *   *  *
```

```
Pairwise SIM alignments to chicken sequence

Gallus gallus          TGCTGATAAGCAGTGAGAAGGAAGGTTTGTCATTAAAAGCCCCTGAATATGGTGTAGAAA
Monodelphis domestica  TGTTGATTGAGAATGAGAAA-ACATTCTCTTGCTAATAGCACCAGACTATGGAATTAATA
Macropus eugenii       NNNNNNNNNNNNNNNNNNNNNNNNNNNNNNNNNNNNNNNNNNNNNNNNNNNNNNNNNNNN
Wallabia bicolor       NNNNNNNNNNNNNNNNNNNNNNNNNNNNNNNNNNNNNNNNNNNNNNNNNNNNNNNNNNNN


Gallus gallus          AACTATATTACGATAGACGTAAACTT---------GAG
Monodelphis domestica  AACTTGTTTTTTAATCATGAAACCACCTACCGTACAAG
Macropus eugenii       NNNNNNNNNNNNNNNNNNNNNNNNNNNNNNNNNNNNNN
Wallabia bicolor       NNNNNNNNNNNNNNNNNNNNNNNNNNNNNNNNNNNNNN


Pairwise SIM alignments to chicken sequence

Gallus gallus          AT--------TCGGGTTGCTTTCTGGATGGTTGGAAAAACATGTGGTATTTGTGGAAAAT
Monodelphis domestica  ATCACACAGCTCTAACTTTCCTCTAGAAAGTGAGAAAAACAAACGGTTTGTGTGGCAATT
Macropus eugenii       NNNNNNNNNNNNNNNNNNNNNNNNNNNNNNNNNNNNNNNNNNNNNNNNNNNNNNNNNNNN
Wallabia bicolor       NNNNNNNNNNNNNNNNNNNNNNNNNNNNNNNNNNNNNNNNNNNNNNNNNNNNNNNNNNNN


Gallus gallus          ATGATGCTGAGAAGAAAAGGGAGTATCAA-ATGCCCAGTGGATATTTAGCTAAAGATGCA
Monodelphis domestica  CGGATTC--ACAAAGTAAACGAGAATCAAGATTCCCAATGGGGATC-AGCTAAAGATGCA
Macropus eugenii       NNNNNNNNNNNNNNNNNNNNNNNNNNNNNNNNNNNNNNNNNNNNNNNNNNNNNNNNNNNN
Wallabia bicolor       NNNNNNNNNNNNNNNNNNNNNNNNNNNNNNNNNNNNNNNNNNNNNNNNNNNNNNNNNNNN


Gallus gallus          GTGAGTTTTGCTCAGTCCTGGGTCATCTCAGAAGACACGTGT
Monodelphis domestica  TTAAGCATTGCTTGCACTTAGATCTTGGCAGATGATACCTGT
Macropus eugenii       NNNNNNNNNNNNNNNNNNNNNNNNNNNNNNNNNNNNNNNNNN
Wallabia bicolor       NNNNNNNNNNNNNNNNNNNNNNNNNNNNNNNNNNNNNNNNNN


Pairwise SIM alignments to chicken sequence

Gallus gallus          CTTGCAAGCTGCAGAGGAAATTTGTCAAAATTGAGAAGCCGGTTGCATTT---AACAAAA
Monodelphis domestica  TATGTGGACTGTTCTGAAAAGTCATAAAGATTGAGAAAATGAT----------GAAAAGA
Macropus eugenii       AATGTCGACt-tTCTGAAAACTCATAAAAATTGAAAAAATTATTATTGGTTATGAAAAGA
Wallabia bicolor       AATGTCGACt-tTCTGAAAACTCATAAAAATTGAGAAAATTATTATTGGTTATGAAAAGA
                         **    **     * *** *  * ** ***** **     *           * ** *


Gallus gallus          AGGCCTCAAAATGCTTTTCCATTGAGCCAGTTTTAC----GCTGTGCAGAAGGCTGTTCA
Monodelphis domestica  AAGCTGTGACGTGCTTGTCAATTCAGCTGGTTTGAGCTCAGCAGCGGAGGTGGTTGTCCA
Macropus eugenii       AAGCTGCAACTTGCTTGTTGAYTCAGCTGATTTTGGCACAGTGGTGGGGGGAGTTGGCTA
Wallabia bicolor       AAGCTGCAACTTGCTTGTTGATTCAGCTGATTTTGGCACAGTGGTGGGGGGAGTTGGCTA
                       * **    *  ***** *  * * ***   ***       *  * *  *   * **   *


Gallus gallus          GCAACCAGGACTGTTCCTGTCTCTGTGGGTTTCCACTGTGTCCCATCTG
Monodelphis domestica  TGAA----AACTGCCCTGGTCTCTGTGGGCTTTCATTGTCTAGTAGCTT
Macropus eugenii       TGAA----AACTGCYTGGGTCTCTGTGGGCTTCTGCTGTTTAGCAGCTG
Wallabia bicolor       TGAA----AACTGCCTGGGTCTCTGTGGGCTTCTTCTGTTTAGCAGCTG
                         **     ****     *********** **    *** *   * **
```
