# Supplementary material for: Loss of Egg Yolk Genes in Mammals and the Origin of Lactation and Placentation
Source: PLoS Biol. 2008 Mar 18;6(3):e63. doi: 10.1371/journal.pbio.0060063 (PMC2267819; doi:10.1371/journal.pbio.0060063)
Supplement: Supplementary file 4 [file pbio.0060063.sg004.pdf]

Figure S4

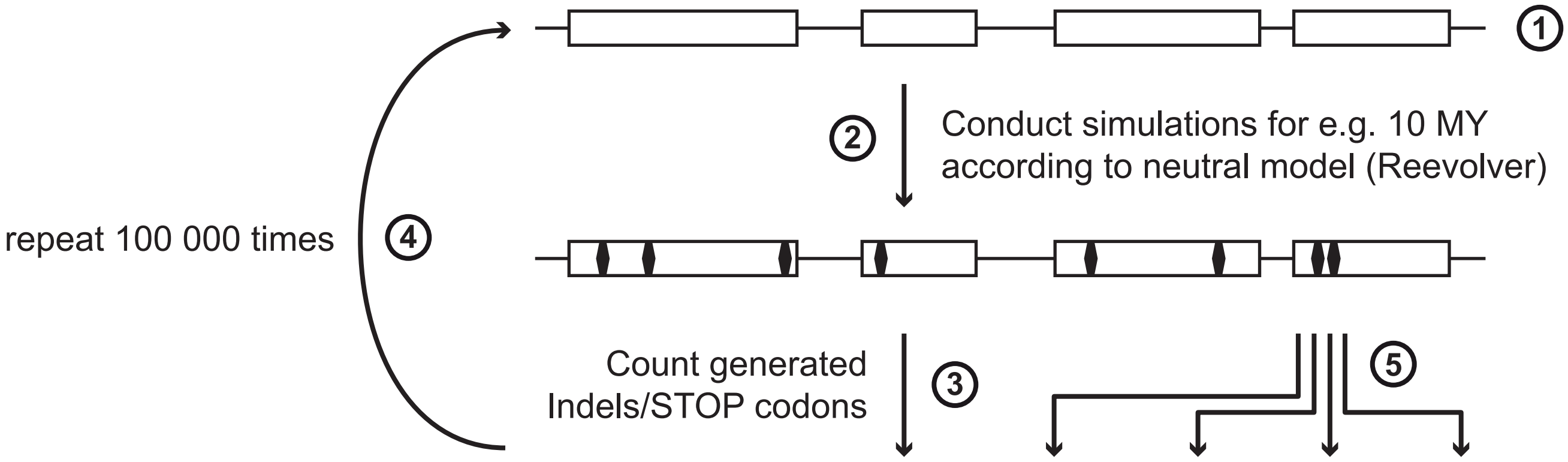

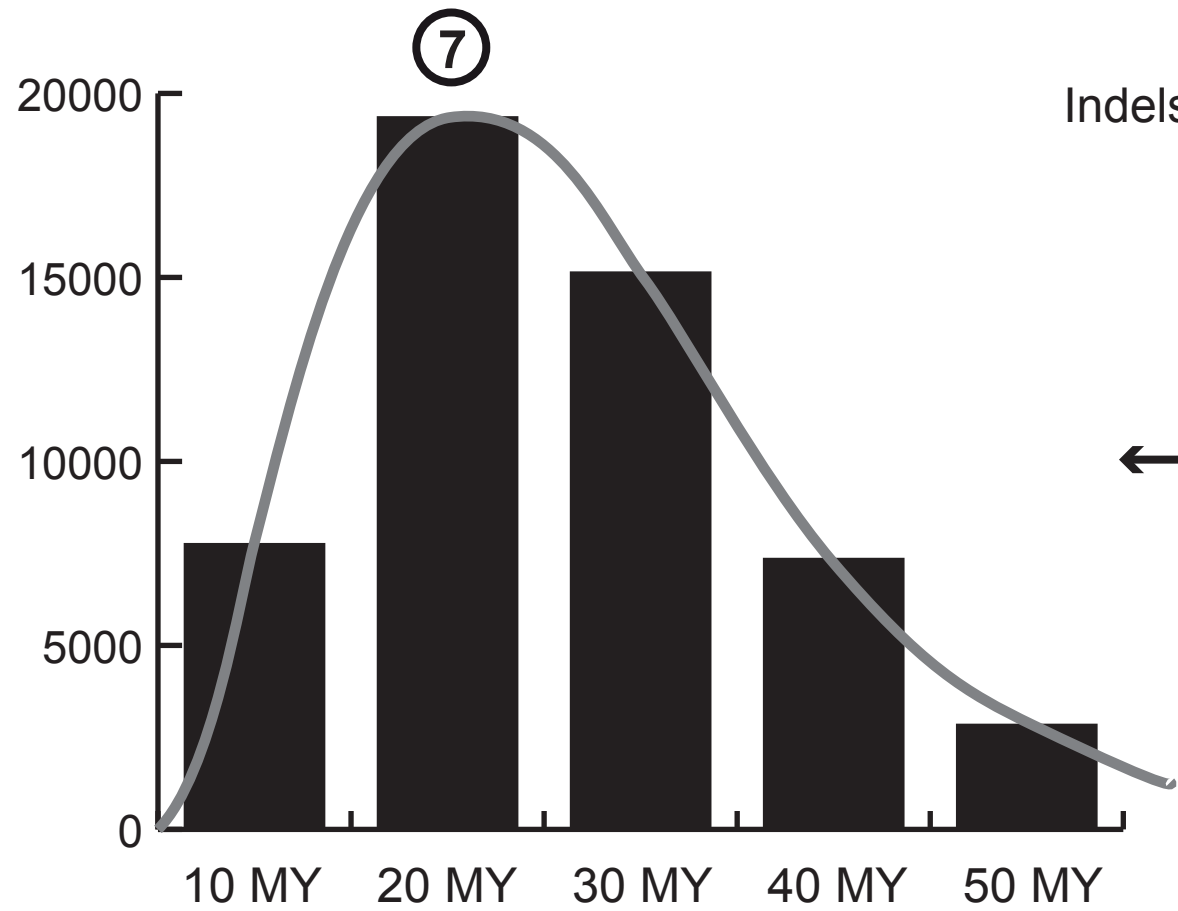

6

Number of Indels/STOP codons	10 MY	20 MY	30 MY	40 MY	50 MY	n
0	15305	2447	405	20	3	
1	26967	16610	5789	1614	210	
2	7748	19346	15128	7345	2837	
3	913	9034	16093	13721	8819	
4	19	2253	8977	13841	12571	
m						
