# Supplementary material for: Loss of Egg Yolk Genes in Mammals and the Origin of Lactation and Placentation
Source: PLoS Biol. 2008 Mar 18;6(3):e63. doi: 10.1371/journal.pbio.0060063 (PMC2267819; doi:10.1371/journal.pbio.0060063)
Supplement: Supplementary file 5 [file pbio.0060063.sg005.pdf]

Figure S5

a. *VIT1*

```
Gallus gallus VIT1    ATGAGGGGTTTAATATCAGCCCTGGTTTTAACCCTGGTGGGGAGTCAGCATCTTAATTAC
O. anatinus VIT1      ATGAAAGGGTTCATTTTCTCCCTACTTTCTACTCTTTCAGGGAGTCAGCGTCTTAATTAT
                      ****  ** ** ** *   ****  ***  ** **    ********** *********

Gallus gallus VIT1    CAACCTGATTTTGGTGAAAATAAGGTTTATACATACAATTATGAAAGCATACTTTTCAGT
O. anatinus VIT1      GCACATGACTTTGTGGAAGGCAACATTTACACCTACAGTTGTGAAAATATGCTTCTTAAA
                        ** *** ****  ***   **  **** ** **** ** *****  ** *** * *

Gallus gallus VIT1    GGTATTCCAGAGAAAGGACTTGCAAGAACTGGAATAAGGATAAGAAGTGAAGTGGAAATT
O. anatinus VIT1      GGATTGCCAGAATGGGGACTTGGAAGAGCTGGAATAAAACTGAATTGTAAAGTGGAAATC
                      **  * *****    ******* **** *********   * *   ** **********

Gallus gallus VIT1    AGTGGTATTGGACCAAAACTTTGTCTTATTAGGATTCACTCAATTGAAGCAGCAGAGTAC
O. anatinus VIT1      AGTGGCACTGGGCCAAAATTCAGTCTTATTAGG-----TTCAGTGGAAACCTTCGATTAG
                      ***** * *** ****** *  ***********      *** * *** *    ** **

Gallus gallus VIT1    AATGGCATCTGGCCTACAAGCTCATTCTCTCGGTCACTCAAACTGACTCAGGCACTAACT
O. anatinus VIT1      AA----ATCTGGCCAGGAGACTCATTTATTCCATCACCTGAACTCATCCAGTCACTGGTG
                      **    ********   *  ******   **  ****   **** *  *** ****

Gallus gallus VIT1    GGACAGCTTAGTATTCCCATCAAGTTTGAGTATAGCAATGGCCACGTTGGAAACCTTATG
O. anatinus VIT1      ACACAACTTC-TACAGCCTTTGAAATTTAGGTCAGCAGAGGTTGGATTGGAAGCAGATAT
                        *** ***  **   ** *  *  ** **   ****  **     ****** *

Gallus gallus VIT1    GCTCCTGATTCTGTCTCAGATGATGGTCTGAACATCTACAGAGGGATTCTCAATATTCTG
O. anatinus VIT1      GCTCCAGATTCAGTATCAAATGCTGCCCTTAATATCTATAGAGGGATCCTTAATGTACTT
                      ***** ***** ** *** *** **  ** ** ***** ******** ** *** * **

Gallus gallus VIT1    GAGCTCAGTCTGAAGAAGATGCAGCATTCATACAGTATACAGGAGGCTGGGATTGGAGGT
O. anatinus VIT1      GAAATTACCATCGAGAGGACAGAAACCACGTACAGTTTACAGGAGATGGGGATCGGAGGA
                      **  * *   *  *** **   *     * ****** ********   ***** *****

Gallus gallus VIT1    ATTTGTAACACAACGTATGCAATTCAAGAAAACAAGAAAGCAAACCTAGTTGATGTGACC
O. anatinus VIT1      CTCTGTCACACCAGATACATCATCCAGGAAGACAAGAAGGAAGAGAGCATCACCATCAGC
                       * *** **** *  **    ** ** *** ******* * * *     *     * * *

Gallus gallus VIT1    AAATCCAAGGACCTGAACAGTTGTGAAGAGAAAAGAAACAAGAATTCCCGGGCAACTGCT
O. anatinus VIT1      AAGACCAAGGACCTCAACAGTTGCCAAGACAAGAGAGACAAGACCACGAAAGGGACGGCC
                      **  ********** ********  **** ** *** ******   *    *  ** **

Gallus gallus VIT1    ACCTATAATTACAAAATTAAATACACTCATAATGAAGCTGTAATCACACAAGCTGAAGTC
O. anatinus VIT1      TCGTACACGTACAAGATGAAATACGCGGAAGCGGGCGCCTTGATCACCCAAGTTAAGGCT
                       * ** *  ***** ** ****** *  *    *  **  * ***** **** * * *

Gallus gallus VIT1    GAGGAAGTCCACCAGTTTACACCTTTCCATGAGATAACAGGAGGAAATGCTATCGTAGAA
O. anatinus VIT1      CAGCAGGTGTATCAGACGGCTCCCTTCAACGAACTCAACGGCGT---TGCCGTCGCTGAA
                       ** * **  * ***    *  ** *** * **  * *  ** *    ***  ***  ***
```

```
Gallus gallus VIT1    GCAAGGCAGAAACTTGCATTGATTGAAGTGCAAAAGCAAG--TGGCAGAAGTTCCACCAA
O. anatinus VIT1      TCCAAGCAGAAGCTGGTGCTTAATGATGTTCAAAAGCAATCCTAACACAGCTTTTTCATA
                       * * ****** ** *   * * *** ** *********   *  ** *  **   *  *

Gallus gallus VIT1    AAGAATTTCAGAAACGTGGATCACTTCAGTACCAGTTTGGCAGTGAATTGCTTCAGCTTC
O. anatinus VIT1      CCGACTTCCAGAAATGAGGATCACTCCTTTACCATTTTGGGAGTGAACTGCTTCAAATTC
                        ** ** ****** * ******** *  ***** ***** ****** *******  ***

Gallus gallus VIT1    CTGTCCACTTATTCAAAATAGAGGAAAGACTGCAAGACTTAGTAGAGACGACATATGAAC
O. anatinus VIT1      CTGTGCATCTCTCCAAAATAGCACACACGCTTCAATATTTGGTCATTCACACCTGTTAAA
                      **** **  * * ********   * *  ** *** * ** **       ** * * **

Gallus gallus VIT1    AGCTTCCCAGTGATGCACCAGCAAAAGCCCTCAAGCTTATGCACCTCTTGC-GTGCTGCA
O. anatinus VIT1      GCACTCCTAGGGACAGCCCTGCTACCTTCCCTCAACTCATCCAAATGTTCCCGAAATGCA
                          *** ** **    ** ** *    **   * ** ** **  * ** * *   ****

Gallus gallus VIT1    AATGAGGAGAACTATGAGTCAGTGTGGAAACAGTTCTCAAGTCGGCCAGCATACAGGCGC
O. anatinus VIT1      AGTGATCACAACATAAAATCCATTTGGAAGTAGTATTTCAGCCAGCTGGCTCATAGGTTT
                      * ***  * ***    * **  * *****  ***  *  ** * **  **  * ***

Gallus gallus VIT1    TACCTTTTGGATTTACTT-CCTGCAGCAGCCAGTCACCGTTCACTGAGATTCTTGAGGCA
O. anatinus VIT1      TTGATATTGGATACAGTGATTTCCAGTTTCCAGTGTTCCCATAATCAAGTTCATCAGGCA
                      *   * ******  * *    * ***   *****   *    * * *  *** * *****

Gallus gallus VIT1    TAAAATGGAAAGGCAAGAGGCTCAGACAGTGCTTGTGGCTCTCCACTCAAGCTCACCAAC
O. anatinus VIT1      CTGGAGAGAAAAGGGAGAGGCTCAGACTGTGCTCGCATACCTACATTTCAGCACAGCTAC
                          *  **** *  ************ ***** *     ** ** *  *** ** * **

Gallus gallus VIT1    TCAAGAAGTGATGGAAGAAGCTACATTCCTTATGAGTGGACTGGCAGCTAAATATTTTGT
O. anatinus VIT1      CCATGTCATCATAGCTAAGGGTGCGTCTCTTATGGTGGGAATTGCAGCTAAGTATTCCTC
                       ** *   * ** *   * * * * *  ******   *** * ******** ****

Gallus gallus VIT1    ACTGAATAATGCAGGAAGCTTAATTCCAACAATGGCAGTGTCCCAGCTGCGGACACATTT
O. anatinus VIT1      AATAAAAAGTGCAGGCAGCATCTTTCCATCGGGGATCATTTCTTCAGTCAAGGCAAATTT
                      * * ** * ****** *** *  ***** *   *    * **     *   * ** ****

Gallus gallus VIT1    CCTTGGAAGAGTTGCTGATCCCATAGAGGTGGGAATAGCAGCTGAAGGACTTCAGGAGAT
O. anatinus VIT1      CCTTGGAAACACTGCTGATATGTGGGAGAGGGGAATGCTCACTCAACGAGTTCTAGAAAT
                      ********    *******      ***  ******     ** ** ** ***  ** **

Gallus gallus VIT1    GTTTGTTAGAGGTTATTCTCCTGACAAGGACTGGGAGACTAACTATGACTTCAGGGAAAT
O. anatinus VIT1      GATCATGCAAAGC-ATCCATTGGGAAGCGACTCTTTCAGTGTAGATAGGATCAAGGGTAT
                      * *  *   * *  ** *    *  *  ****     * *    **    *** **  **

Gallus gallus VIT1    CCTAAAAAAGCTGTCGGACTGGAAGGCACTCCCCCGTGACAAACCTTTTGCATCAGGTTA
O. anatinus VIT1      TTTAAAAATGCTTTCAGACTGGAAAGCAATATCCACAGATAAACCTATCGTCTCGGCCTA
                        ****** *** ** ******** *** *  **   ** ****** * *  ** *  **

Gallus gallus VIT1    CTTGAAGATGTTTGGCCAGGAGTTGCTCTTTGGTAGACTCGATAAAGACACCCTCCAAAA
O. anatinus VIT1      TGTGAAGTTATTTGGACAAGGGATTTTCTTTGCCGCCCTTGAGAAACATTTCATCCAAAA
                        ***** * ***** ** * * *  ******     ** ** *** *   * *******
```

```
Gallus gallus VIT1    TGTATTGCAGGTATGGTATGGACCTGATGAAAAAATCCCTTCAATAAGGAGATTAATCAG
O. anatinus VIT1      TGCAATTCAGACT--------GCTTGAGGAAAAGCTTCAAGCAGTCAAGAACATCATTCA
                      ** * * ***            * *** *****  * *   ** * * **   * **

Gallus gallus VIT1    TAGCCTTCAAACTGGCATAGGAAGACAATGGACTAAGGCTTTACTATTGTCTGAGATTCG
O. anatinus VIT1      TATTTTTCAAAATGGCTTCACAGTCCAGTGAGCTAAACCACTGCTCTTTTCTAAAGTCCA
                      **   ****** **** *   *   ** **  ****  *  * ** ** *** *  * *

Gallus gallus VIT1    TTGTATTGTGCCTACCTGTGTTGGGTTCCCGATGGAGACCAGCTTCTATTACTCTTCTGT
O. anatinus VIT1      ACATATCATACCAACATTTACTGGTCTGCCCATAGAAAAAGGTTTCTATTA-TACACTGT
                         ***  * ** ** * *  ***  * ** ** ** *   * ******** *   ****

Gallus gallus VIT1    CACAAAAGTGGCAGGAAACGTTCAAGCGCAAATTACACCTTCACCGAGGTCTGATTTCAG
O. anatinus VIT1      CACAAACATGGTCACCAAAGCTCATGCTCAGATTTCACC--CACC----TCATCTTTTAG
                      ******  ***     ** * *** ** ** *** ****  ****    **   *** **

Gallus gallus VIT1    ATTGACTGAGTTAC--TAAATTCCAACGTTAGGCTGCGATCCAAAATGAGTCTAAGCATG
O. anatinus VIT1      ATGTGCTGACTTACATTTGATACCAGCAATAAATTGCATTTAAAAACAACTCTGAGCATG
                      **   **** ****  *  ** *** *  **   ***  *  ****  * *** ******

Gallus gallus VIT1    GCTAAACATATGACCTTTGTAATT---GGGATCAACACAAACATGATCCAGGCAGGGCTG
O. anatinus VIT1      GCTAAAGAGATGA---TTGCAATTTTGGGAATTAATGACCATCGCCTTTAGGCTGGACTG
                      ****** * ****   *** ****   ** ** **     *     *  **** ** ***

Gallus gallus VIT1    GAAGCACACACTAAAGTAAATGCTCATGTACCTGTGAATGTTGTTGCCACTATTCAAATG
O. anatinus VIT1      GAAGTCTATTCGGGAGTTACGATGGATGTCCCTGCGAATTTTACTTCTTC-ACTTGTTTT
                      ****   *  *   *** *      **** **** **** **  * *  * * *    *

Gallus gallus VIT1    AAGGAAAAAAGTATCAAAGCTGAAATTCCACCATGCAAAGAAGAGACTAACTTAATTATT
O. anatinus VIT1      AAGGAAAATAATTTCACCTTTGAATTTCCCCCAAGCAATCAGGAGACTGAGTTTGCTTCT
                      ******** * * ***    **** **** *** ****  * ****** * **   *  *

Gallus gallus VIT1    GTAAGCTCTAAGACATTTGCTGTTACACGAAATATTGAAGATTTGGCTGCTAGTAAGATG
O. anatinus VIT1      TTGAGGTCTAAAGCATATGCTGTTACTCAAAATATTGAAGGTGCATCTAAAACAAAAATG
                       * ** *****  *** ********* * *********** *    **   *  ** ***

Gallus gallus VIT1    ACTCCAGTTCTTCTACCTGAAGCAGTGCCTGACATAATGAAGATGTCCTTCGACTCAGAT
O. anatinus VIT1      ACTCTAATTATTGTACCAGAATTCAGACCTAGCATACTTAAAGGTCCCTTCAATTCAGGA
                      **** * ** ** **** ***      ***  **** * **     ***** * ****

Gallus gallus VIT1    TCTGCATCAGGCGAGACTGATAACGACAGACAGTCTGTAGAAGATGTTTCATCTGGAAAT
O. anatinus VIT1      TTAGAATCAGGAGAGAATACTCAAGACCGACTGTCTTCCAAAATCACATCAACAGGAATT
                      *  * ****** **** *  * * *** *** ****    **      *** * **** *

Gallus gallus VIT1    TCCTTCTCCTTTGGACATCCTTCTTCCGGGAAGGAGCCATTTATTCAGTCCATGTGCTCC
O. anatinus VIT1      ATGGATTCAGTTGGAATGCCGTATCACTGAACAGCTCCATTTTTTCATTCCTATTGTTAT
                            **  *****   ** * *  * * *  *  ****** **** ***   ** *

Gallus gallus VIT1    AACGCAAGTACATTTGGGGTTCAAGTGTGCATTGAGAAGAAAAGTGTACATGCAGCATTC
O. anatinus VIT1      AACACAAGTACCTTTGGATTTCAATTTTCTAT------GAAACCTA---------CATTT
                      *** ******* *****  ***** * *  **      ****  *          ****
```

```
Gallus gallus VIT1    ATCAGAAATGTGCCTCTTTATAACGCTATTGGAGAACATGCCCTTAGAATGAGCTTCAAG
O. anatinus VIT1      ATTAGAGACATTCTCTATTACTACCTAACTGGGAAACACATCATAACCGTGACTATCAAG
                      ** *** *  * *    ***  **   * ***  ****   * * *   ***   *****

Gallus gallus VIT1    CCAGTCTACTCAGATGTACCTATTGAAAAAATACAAGTCACAATTCAAGCAGGAGATCAA
O. anatinus VIT1      CCAGTTCAATCAGATATGCCAATTGAAAAAATGCATATAATAATTCAGACTGGTTCTCAG
                      *****  * ****** * ** *********** **    * ******  * **   ***

Gallus gallus VIT1    GCTCCTACAAAAATGGTACGTTTAGTAACATTTTCCAGAAAAGAAGTGATGAAAA-----
O. anatinus VIT1      GCACCTGGAGAGGAGACACGTTTAGTAAAGTTTTCCAGAAGAGCAGTACTGAAAACCACT
                      ** ***  * *   *  ***********  ********** ** ***  ******

Gallus gallus VIT1    ------GAGTGAAGAAAATCCTTGATGACACTGATAACCAGCATTCAGGACACAGGACAT
O. anatinus VIT1      ACTCTAGGGCAAGAACAACCTAAAATGTCATTAAAAAGAAGGAAATGGAGAATAAAATGT
                            * *  *  * ** *    *** ** * * **  ** *    *   * *  *  *

Gallus gallus VIT1    CAAGCAGCTCATCATCATCTTCCTCTGAGTCTGGAAGCAGCCACAGCAA---CAGCAGCA
O. anatinus VIT1      TAGGTGGCTCAT------CTTCCTCCAAGTT---AAGCAGTGACAGCCAACACAGCAGCA
                       * *  ******      *******  ***    ******  ***** *   ********

Gallus gallus VIT1    GCAGTGACAGCCGCAACTTCCTGGGAGACGTAATTCCACCTGGCATTACGATTGTGGCAC
O. anatinus VIT1      GTAGGGAGAGCTACAAATTCCTAGGAGAGATATTGCCTCCAAGCTTAACTGCTATCGTCC
                      * ** ** ***  *** ***** *****  ** * ** **  ** * **   * * *  *

Gallus gallus VIT1    AGGCAGTAAGAAGTGACAACAGAAATCAAGGTTATCAAGCTACAGCATATGTTCGTTCCG
O. anatinus VIT1      AGGCCATTAGGTTAGATAATAAAGATCAAAGTTATCAAGTCACAGCATATATCAGCTCTG
                      ****  * **    ** ** * * ***** *********  ********* *  * ** *

Gallus gallus VIT1    ATGCTGCCAAAGTTGATGTGCAACTAGTTGTGGTTCAGCTGGCTGAAACCAATTGGAAAG
O. anatinus VIT1      AGAGGGCTAAAGTTGACAGGCAGTTAGTGGTGGTTCAGCTGTCTAATGGAAGCTGGCAAG
                      *    ** ********   ***  **** ************ ** *    *  *** ***

Gallus gallus VIT1    CATGTGCTGATGCTGTAATACTGCCTCTGAAAGCACAGGCTAGAATGAGATGGGGCAAGG
O. anatinus VIT1      TATGTGGGGATGCCATAGTGGCATCTCTCAAGGCAAAGGCCAGGCTCAGAAGGGTCCCAG
                       *****  *****  ** *     **** ** *** **** **  * *** *** *   *

Gallus gallus VIT1    AGTGCCGAGACTACAGGATAGCAGCCTGGGCTACCACAGGCCAAATGGCAAGGAAGCTAG
O. anatinus VIT1      AATTCCTGGACTACCCAACTGAGGCAATGGGTTCCATGAGCCAGATTGCAGGAAATCCAG
                      * * **  ******   *  *  **   ** * ***   **** ** *** * ** * **

Gallus gallus VIT1    CCGTGCAGCTGAAGGTGCAATGGGGAATTATTCCTTCCTGGATACTCATGCGGTATGTTC
O. anatinus VIT1      CCATTCAGCTGAAGGTGGGCTGGTCAAAAGTTCCAACTCAGATCAAAATGAAATCTGCTC
                      ** * ************   ***  **   ****  *   ***    ***   * ** **

Gallus gallus VIT1    CTGGCGTGGCACTTGTGTTGGGCTTCTCAGAGGCGCATCAGAGAAACCCATCTCGTGAAC
O. anatinus VIT1      CTTGGATTGCCTTGTTTTCAGGCTTTTCTGAAGCACAAAAGTGAAAACCCTCCCACCGAA
                      ** *  * **  *  * *  ***** ** ** ** **  ** **** ** ** *    *

Gallus gallus VIT1    TTATTGTACGGGCAGTGGCAACATCTCCACGATCAATTGATACAGTAATTAAAGTTC--C
O. anatinus VIT1      AAGTTTTTAAGTCAGTGGTATCATCCTCATGCATAATTGATAAAGCTGTTACAACACTGC
                         ** *   * ****** * ****  ** *   ******** **   *** *   *  *
```

```
Gallus gallus VIT1     TGGGACATGTGAAGTCAGTAAGGGAGATTTCAAAACAT-TTGATCGCATGAGCTTTACCT
O. anatinus VIT1       AGGGGACTGTGAAGT-AGGAGAAAATATCTGGAAAAACATTGACAGGGTTGAATACAATA
                        ***   ******** ** *    * ** *  *** *  ****  *  *    *  *

Gallus gallus VIT1     GTTCTTTCAATAAAAGCTGCAATGTGGTAGTGGCCCAAGATTGTACCGAACATCCAAAAT
O. anatinus VIT1       ATTCATATCCCAAAGCCTGTGATCTTCTTGTAGCCTAAGTCTGCCAAGCTGCACCAGCAT
                        *** *     ***  ***  ** *  * ** *** ***  **    *     ***  **

Gallus gallus VIT1     TCATCATTACTACAAGAAAAGTTGATCACCAGTCTTTGT-CAAGAGAGGTTCACATAAAT
O. anatinus VIT1       TTGTTATTTTGACAAAAGATGT----CACAACTTCTACTGCAAGAGAAGTGTCTATCTTT
                       *  * ***   **** * * **    *** * *  *  * ******* **    **   *

Gallus gallus VIT1     ACAAGTTCAAATATTAAAATTTATAAAAATGGAAAAACAGTTATTGTGGAAGCTCCAATA
O. anatinus VIT1       CTGGGAAAACCCATTGAAATTTTCAAAAAGGAAGGAAATGTTACAGTGTTGGTACCTGAA
                           *   *   *** ******  ***** * *  **  ****  ***   *  **   *

Gallus gallus VIT1     CATGGACTGAAGAATGTGAATTTTGATGGTGAGATCCTTAAGGTTACTGTTGCTTCATGG
O. anatinus VIT1       TTTGGACTGGAAAAACTGAGTTAAGATGGAAAAAATATTAGAATCATTGTTACTTCATGG
                         ******* * **  *** **  *****  * *   ***   * * **** ********

Gallus gallus VIT1     ATGAGAGGAAAGACCTGTGGAGTCTGTGGAAATAATGACAGAGAAAAGCACAATGAACTC
O. anatinus VIT1       ATGAAAGGGAAGACCTGTGGAAC-----------ATGAATGGGGAGATTTAAGTGACCCG
                       **** *** ************             ****  * * * *    * *** *

Gallus gallus VIT1     CTAAT-GCCAAATCATAAGCTGTGCAAACTGCAGCGCAGTTATGTGAAGCTGAATCGAAA
O. anatinus VIT1       GAAATTGCCAAATCACAAGCAGTGCAAACTCCAAAGCAAGTATGTGG-------TCTCGG
                         *** ********* **** ********* **  ***  ******        **

Gallus gallus VIT1     TCCTACTATTGA-TGGAGAGGAATCTACATGCTACTCCGTTGACCCAGTACTGAAATGTA
O. anatinus VIT1       GCCTTGTAGTGAACGGAGGAGAATCCAAATGCTACTCCTCAGCACCAACATTGAAATGTA
                        ***  ** ***  ****  ***** * **********   *  ***  * *********

Gallus gallus VIT1     TGAAAGACTGTACTCCAACTGCTGTAAGCCTGTTGGAATGGCAGAGAAGCA-GCGATAAG
O. anatinus VIT1       CAAATGGCTGTACAGCATCATCAGTAAATATGACTGACTG-CAAGGATGCACATGACAAG
                         ** * ******  ** *  * ****   **   ** ** **  ** ***   ** ***

Gallus gallus VIT1     AAATCTGCATCTGAGGATGTTGTGGAGTCTGTGGATGCTGACATTGATTGTACCTGCACT
O. anatinus VIT1       CAATCTCTAGCAGACGGCAAAGTGAAGACAGTAGCTACTGATATTGATTGTACGTGTATG
                        *****  * * ** *     *** ** * ** * * **** *********** ** *

Gallus gallus VIT1     GGTGACTGTAGTTAA
O. anatinus VIT1       AGTGACTGTTCTAGA
                        ********  *  *
```

b. *VIT2*

```
Gallus gallus VIT2     ATGAGGGGGATCATACTGGCATTAGTGCTCACCCTTGTAGGCAGCCAGAAGTTTGACATT
O. anatinus VIT        ATGAGGGCATTCATCTTGGCCCTGCTAGTCTCCCTGGCGGNNNNNNNNNNNNNNNNNNNN
                       *******   ****  ****  *  *  ** **** *  *

Gallus gallus VIT2     GACCCAGGATTCAATAGCAGAAGGAGTTACCTGTACAACTATGAAGGTTCTATGTTGAAT
O. anatinus VIT        NAGCCAGAATTTAGCAGCAGCAAGACCTACGTGTACAGTTACGAAGGACAGCTCCTCAGT
                        * **** *** *  ***** * **  *** ******  ** *****     *  * * *

Gallus gallus VIT2     GGGCTTCAAGACAGAAGTTTGGGCAAAGCTGGTGTGCGCTTGAGCAGCAAGCTAGAGATC
O. anatinus VIT        GGGTTGCCAGACAGAGATCTGGCGAGAGCTGGGTTGAAAATCTCGTGCAAAGTGGAGATT
                       *** * * *******  * ***  * ******  **    *     ****  * *****

Gallus gallus VIT2     AGTGGGCTACCAGAGAATGCTTACCTCCTCAAGGTCCGCTCTCCACAAGTGGAGGAGTAC
O. anatinus VIT        AGCGGAGCATCAGAGAAGGCACACTACCTCAAGNNNNNNNNNCCACAAGTAGACGAATAC
                       ** **   * ******* **  **  *******         ******** ** ** ***

Gallus gallus VIT2     AATGGGGTCTGGCCCAGGGATCCCTTCACTCGATCTTCCAAAATCACCCAAGTGATCTCA
O. anatinus VIT        AACGGCTACTGGCCCNNNAATGCCTTCGTTCACTCTTCCAAGCTCACCCGTATCCTCGCT
                       ** **   *******    ** *****  **  ********  ******   *  ** *

Gallus gallus VIT2     TCGTGTTTCACCCGGCTCTTCAAATTTGAATACAGCAGTGGACGGATCGGAAACATTTAT
O. anatinus VIT        TCCCACCTGAACGCGCCCATCAAATTCGAATATGTCAAAGGGAGGGTCGGAGCCATTTTT
                       **     * * *  ** * ******* *****   **  **  ** *****  ***** *

Gallus gallus VIT2     GCCCCAGAAGACTGCCCAGATCTGTGTGTTAACATAGTGAGAGGAATATTGAACATGTTC
O. anatinus VIT        GCCCCGGAGGACATGTCCGACACCGTTGTGAACATACTGAGAGGAATCCTCCAAATGTTG
                       ***** ** ***    * **      *** ****** **********  *  * *****

Gallus gallus VIT2     CAGATGACCATTAAAAAATCACAGAACGTCTACGAATTACAAGAGGCTGGAATTGGAGGT
O. anatinus VIT        CACTTGACCATCAAGAAAACCCAGTCGGTGTATGACCTGCAGGAGATGGGGATCGGAGGA
                       **  ******* ** *** * ***   ** ** **  * ** ***   ** ** *****

Gallus gallus VIT2     ATTTGTCATGCAAGGTATGTCATTCAGGAAGACAGGAAGAATAGCCGAATCTATGTTACC
O. anatinus VIT        CTCTGTCACACCAGATACATCATCCAGGAAGACAAGAAGGAAGAGAGCATCACCATCAGC
                        * *****  * ** **  **** ********** **** *     * ***    * * *

Gallus gallus VIT2     AGAACTGTGGACTTGAATAATTGCCAGGAAAAGGTGCAAAAAAGCATTGGAATGGCTTAC
O. anatinus VIT        AAGACCAAGGACCTCAACAGTTGCCAAGACAAGGTGGTGAAGAGCAGCGGGATGGCTTAC
                       *  **   **** * ** * ****** ** ******   ** ****  ** *********

Gallus gallus VIT2     ATCTATCCCTGCCCTGTGGACGTGATGAAAGAAAGGCTCACCAAAGGGACCACCGCTTTC
O. anatinus VIT        AAGTTCACCTGCCCTTTCTGCGACAAGAGAGACAAGACCACGAAAGGGACGGCCTCGTAC
                       *  *   ******** *   **  * ** *** * *  *** ********  ** * * *

Gallus gallus VIT2     TCCTACAAGCTGAAGCAGTCAGACAGCGGCACGCTGATCACAGATGTCTCGTCGCGGCAG
O. anatinus VIT        ACGTACAAGATGAAATACGCGGAAGCGGGCGCCTTGATCACCCAAGTTAAGGCTCAGCAG
                        * ****** ****  *  * **    *** *  *******  * **   * * * ****
```

```
Gallus gallus VIT2    GTGTATCAGATCTCCCCATTCAATGAGCCCACTGGGGTGGCTGTCATGGAAGCAAGACAG
O. anatinus VIT       GTGTATCAGACGGCTCCCTTCAACGAACTCAACGGCGTTGCCGTCGCTGAATCCAAGCAA
                      **********   * ** ***** ** * **  ** ** ** ***   *** * *  **

Gallus gallus VIT2    CAGCTCACTTTGGTCGAGGTGAGAAGTGAGCGGGGCAGTGCCCCAGATGTCCCCATGCAG
O. anatinus VIT       GTATTTACTCTGCTTGAAATTAAAAGCAAA---CAGACCCCGCCGGAAATACACCTGCGG
                          * *** ** * **  * * ***  *       *   * ** **  * * * *** *

Gallus gallus VIT2    AACTATGGCAGCCTTCGCTACCGCTTCCCAGCCGTACTGCCACAGATGCCACTTCAGCTG
O. anatinus VIT       AATCACGGAAACATCCGGTACCATTTCCCTAAAGAGATGCAACAGATGCCGATGCAGATA
                      **  * ** * * * ** ****  *****      *   *** *********  * *** *

Gallus gallus VIT2    ATCAAGACAAAAAACCCTGAGCAACGGATAGTAGAAACGCTGCAGCACATAGTCCTGAAT
O. anatinus VIT       ATAAAGTTAAAAAATCCCGAAGACCGGATCTTGGAGACCCTGAAATATTTAGTACAGGCC
                      ** ***  ****** ** **  * *****  * ** ** *** *  *  **** * *

Gallus gallus VIT2    AACCAACAAGATTTCCATGACGATGTTTCATACAGATTCTTAGAGGTGGTCCAGCTTTGC
O. anatinus VIT       AATCAGCACGATATCAAGGAAGACGCCCCAACCAAATTCCTCGAGCTCACCCTGCTTTGC
                      ** ** ** *** ** * ** ** *   **  ** **** * *** *   ** *******

Gallus gallus VIT2    CGGATAGCAAATGCTGACAATCTTGAGTCTATCTGGAGACAAGTTTCAGATAAACCTCGT
O. anatinus VIT       CGGGCATCGACTTTCGAAGGTCTGGACGCTGTCTGGAAGCAGTACGAGGGGAAGAGCCAC
                      ***  * * * *   **   *** **  ** ******  **       *  **    *

Gallus gallus VIT2    TACAGGCGATGGCTCCTGAGCGCAGTTTCTGCGAGTGGCACCACAGAAACACTAAAATTC
O. anatinus VIT       TACAGGCGATGGCTGCTGAGTGCCATTTCCATGGCCGGCTCTTTTGACTCGTTCAAGTTC
                      ************** ***** **  ****   *   *** *    **  *  * ** ***

Gallus gallus VIT2    CTTAAGAACAGAATTCGCAATGATGACCTCAACTACATTCAGACCCTTCTAACTGTTTCT
O. anatinus VIT       CTGAAAAAAAAGATTCACGGGGAGGAGACCCACTTCACGGAAACAGCTGCCATCCTCTCG
                      ** ** ** *  **** *   ** **   * *** **   * **   *   *   * **

Gallus gallus VIT2    TTGACTCTTCATTTATTGCAAGCTGATGAACACACACTTCCAATAGCAGCAGATTTAATG
O. anatinus VIT       CTGGTCTTCCATTCTATCAGACTCGAAGAGAAACTGATACCTGAGGTCATCGATCTAGTG
                       **    * ****   *   *   ** **  *     * **    *     *** ** **

Gallus gallus VIT2    ACCAGCTCTCGAATTCAGAAAAATCCTGTGCTTCAGCAAGTGGCCTGCTTGGGATATAGC
O. anatinus VIT       ACCAGCTCGCGGATTAAGAAAGACGCCACATTCCTCCAAATTGCACTCCTGGGATACGGT
                      ******** ** *** ***** *  *     * *  *** * **   * *******  *

Gallus gallus VIT2    TCTGTAGTCAACAGATACTGCTCTCAGACCTCAGCATGTCCTAAGGAAGCTCTTCAGCCC
O. anatinus VIT       TCCTTCGTGTACAGATACTGCGCCGCCTCTTCCACCTGTCCGAATGCCGTTCTTCAGGTC
                      **  * **  *********** *     * **  * ***** ** *  * *******  *

Gallus gallus VIT2    ATCCATGACCTGGCAGATGAAGCAATCAGCAGGGGCCGTGAAGACAAAATGAAATTAGCT
O. anatinus VIT       CTCCACGACTTTGCGGCCGAGGCGCTTAGCAAAGGCCACGAGGAAGAGATGGCCATCGCC
                       **** *** * ** *  ** ** * ****  ****  ** **  * ***    * **

Gallus gallus VIT2    CTAAAGTGCATTGGTAACATGGGAGAACCAGCCAGCTTAAAGCGCATCCTGAAGTTCCTT
O. anatinus VIT       CTGAAGGCCCTGGGCAACGCCGGCCAGCCGGCCAGCATGAAGCGCATCCAGAAATTCCTG
                      ** ***  * * ** ***   **  * ** ****** * ********** *** *****
```

```
Gallus gallus VIT2    CCAATATCTTCATCCAGTGCTGCTGATATCCCAGTCCACATTCAGATAGATGCCATAACG
O. anatinus VIT       CCGGGATTTTCATCCACTGCTTCTGATCTCCCCGTCAGGATTCAGGCGGAAGCCGTGATG
                      **   ** ******** **** ***** **** ***   ******   ** *** * * *

Gallus gallus VIT2    GCCTTGAAAAAGATAGCTTGGAAGGACCCCAAAACAGTGCAGGGCTATCTCATCCAGATC
O. anatinus VIT       GCGTTCAGAAAAATGGCTTGGAAGGACCCCAAAAAAGTGCAGGATGTCCTTCTTCAGGTG
                      ** ** * *** ** ******************* ********     **  * *** *

Gallus gallus VIT2    CTTGCAGACCAATCACTTCCCCCTGAGGTGCGAATGATGGCTTGTGCTGTTATCTTTGAG
O. anatinus VIT       TACGTAGACCGCTCGATCCACCCCAACGTCCGGATGGCGGCCTGTGTGATCCTGTTCGAA
                         * *****  **  * * ***  * ** ** ***  *** ****   *  * ** **

Gallus gallus VIT2    ACAAGGCCTGCCCTTGCTTTGATAACGACTATAGCTAACGTGGCAATGAAGGAGAGCAAT
O. anatinus VIT       ACCAAGCCGGGCCTGACTCTGGTCATGATCATAGCCAGTTCGGTGCAAAAAGAGACCAGC
                      ** * *** * ***  ** ** * * **  ***** *    **     ** **** **

Gallus gallus VIT2    ATGCAAGTGGCCAGTTTTGTATATTCCCACATGAAGTCTTTGTCAAAGAGCAGATTGCCA
O. anatinus VIT       TTGCAAGTGGCCAGTTTCACTTATTCGCACATGAAGGTGCTGTCCAAGAGCACGATCCCG
                       ****************    ***** *********    **** *******   * **

Gallus gallus VIT2    TTTATGTACAACATATCTTCCGCTTGTAACATTGCCCTTAAGCTCCTGTCCCCCAAACTG
O. anatinus VIT       GAGTTTGCCAACGTCACCACTGCCTGTGGAATCGCCATCAAATTCCTCAACCCCAAGCTG
                          *   **** *  *  * ** ***   ** *** * **  ****   ****** ***

Gallus gallus VIT2    GACAGTATGAGCTATCGGTACAGCAAGGTCATTCGAGCAGACACTTACTTTGATAACTAT
O. anatinus VIT       GACTATCTGAGCTACCGCTATAGCAAGGCTTTGCGCTTTGAAAAATACGTAGATCCATAT
                      ***  * ******* ** ** *******   * **    ** *  *** * ***   ***

Gallus gallus VIT2    AGAGTTGGTGCTACTGGAGAAATCTTTGTTGTGAACAGCCCAAGAACTATGTTCCCATCA
O. anatinus VIT       CGAGCTGCTGCAATCGGAAGTGTCTACTTGATGAACAGTCCAAAGACTATGTTTCCAGAA
                       *** ** *** *  ***    ***   *  ******* ****  ******** ***  *

Gallus gallus VIT2    GCAATAATTTCCAAATTGATGGCAAATTCTGCAGGTTCAGTGGCTGATCTGGTAGAGGTT
O. anatinus VIT       ACTATAATTTCCAAATTCAGAACCCACTATGCATGTTCGATAACTGATTTTTTTCGAGTTT
                       * ************** *   *  * * **** ****  *  ***** *  * *** **

Gallus gallus VIT2    GGCATCCGAGTGGAAGGCCTCGCAGATGTCATAATGAAAAGAAACATCCCATTTGCTGAA
O. anatinus VIT       AGCATCCGGACTCAAGGCCTGACTGATGTTCTCTGGAAACAAAACACCCCATTTGCTGAC
                       *******     *******  * *****  *   ****  ***** ************

Gallus gallus VIT2    TATCCCACATACAAGCAGATAAAGGAGCTTGGAAAAGCTCTGCAGGGATGGAAAGAGCTG
O. anatinus VIT       TTTTCCATGACCAAGAAGATCAAAGAACTTGTGAAAACTTTATTTGGATGGAAGGCATTA
                      * * ***    **** **** ** ** ****  *** ** *    ******** *   *

Gallus gallus VIT2    CCGACAGAAACCCCTTTGGTATCAGCCTACTTGAAAATACTTGGCCAAGAAGTGGCCTTC
O. anatinus VIT       CCTTCTGAGAAACCCCTGATCTCAGCTGACATCAAAGTATTTGGTCAAGAGCGGTTCTTT
                      **  * ** *  **  ** * *****  ** * *** ** **** *****   *  ***

Gallus gallus VIT2    ATCAACATCAACAAGGAACTCCTGCAACAGGTCATGAAGACTGTAGTGGAACCTGCTGAT
O. anatinus VIT       CTCGATATCAACAAGGACCTTATTCAGAAGGCACTGAAGGTATTCTTCGAACCGGTAGAG
                       ** * *********** **  * **  ***   *****    *  * ***** *  **
```

```
Gallus gallus VIT2    CGAAACGCAGCAATAAAGAGAATCGCCAACCAGATCCGCAACAGCATTGCAGGGCAGTGG
O. anatinus VIT       AGGCACGGCGCGATGAAGAAAGTCGTCACCCAGCTCCAGAATGGCGTTTCCACACAGTGG
                       *  ***  ** ** **** * *** ** **** ***  **  ** ** *    ******

Gallus gallus VIT2    ACGCAGCCGGTGTGGATGGGAGAGCTGCGATACGTGGTTCCCAGCTGTCTCGGCCTGCCG
O. anatinus VIT       ACCCAACCGATGTTGATGGAGGAAGTCAACTACATTGTACCCAGCCTCCTGGGTCTCCCG
                      ** ** *** *** *****  **  *    *** * ** ******   ** ** ** ***

Gallus gallus VIT2    CTGGAGTACGGGTCCTACACCACCGCCCTGGCACGAGCTGCAGTCAGCGTTGAGGGAAAG
O. anatinus VIT       CTCGACCTTAGTTTTTACTCGACCGGCTTTGTTCACGCTTCGGCGTCGGTTGGCATGAAG
                      ** **     * *  *** * **** * * *  *  *** * *     ****     ***

Gallus gallus VIT2    ATGACGCCGCCTTTAACCGGAGATTTCAGACTTTCTCAGTTGCTTGAATCCACCATGCAG
O. anatinus VIT       ATGTCACCCCCTATGTCCAACAACTTCCGGGTTTCACAGCTGTTTGAGTCTGTTATTCAA
                      *** * ** *** *  **    * *** *  **** *** ** **** **    ** **

Gallus gallus VIT2    ATTCGGTCTGACTTAAAGCCCAGTTTATATGTGCATACAGTTGCAACGATGGGTGTCAAC
O. anatinus VIT       GCTCAAGCTGACATTGGTCCAAGCGTGTACTTGCACACGGTGGCGACCATGACGGTGAAC
                        **   ***** *    ** **  * **  **** ** ** ** ** ***   ** ***

Gallus gallus VIT2    ACAGAATACTTTCAACATGCTGTTGAAATTCAAGGCGAGGTCCAGACAAGAATGCCAATG
O. anatinus VIT       ACACCCCTCTTCCAGTCCGGCATCGAGTTCCACACCGAGTTCCAGGCGACTTCCCCGGTG
                      ***     *** **    *   * **  * **   **** ***** * *     **  **

Gallus gallus VIT2    AAGTTTGATGCCAAGATAGATGTGAAATTGAAAAACCTTAAGATTGAAACGAACCCATGC
O. anatinus VIT       AAATTTGACGCCAAGCTGCACATGAAATCCAAAAGTTTCAAGTTTGAAACGAGCCCGTCT
                      ** ***** ****** *  *  ******  ****   * *** ********* *** *

Gallus gallus VIT2    CGTGAGGAAACTGAGATAGTGGTTGGAAGACATAAGGCTTTTGCTGTATCAAGGAACATA
O. anatinus VIT       CATAAAGAAACCGAGCTGACAAAGTTTAGGTCTATAGCTTACGCCGTCTCCAGAAACGTC
                      * * * ***** *** *          **   **  ****  ** ** ** ** *** *

Gallus gallus VIT2    GGAGAACTAGGTGTTGAAAAGAGGACCTCAATTCTGCCGGAAGATGCTCCATTAGATGTT
O. anatinus VIT       GACGAGCCGGACGTGGAGAAGAAGACACCAGTCGTACCTCCGGATGTCCCGAACGACATG
                      *  ** *  *  ** ** **** ***  ** *  * **    ****  **    **  *

Gallus gallus VIT2    ACAGAAGAACCTTTCCAAACATCAGAGAGAGCTTCCAGGGAACACTTCGCAATGCAAGGG
O. anatinus VIT       GAAAAGGTCTATTTTAAATCAACTGAATTAACTTCAAGAGAAAACCCCATGATGCCGGCT
                        * * *    ***  ** ** * **   * **** ** *** **  *   ****  *

Gallus gallus VIT2    CCTGACAGCATGCCAAGGAAACAGTCCCATAGTTCTCGAGAAGATCTTCGCCGTAGCACA
O. anatinus VIT       TACGGAATGATGCCGAAGAAACATCCCCACATGCCTAAAAGACATCATCACACTTTATCT
                         *  *  ***** * ******  **** *   **  *  * *** ** *  *    *

Gallus gallus VIT2    GGAAAAAGAGCACATAAACGAGACATTTGCCTCAAAATGCATCATATTGGTTGCCAGCTT
O. anatinus VIT       GGAAAACAA---TCCACACAGACCTTTTGTGTCAAAGTATATCCTTTTGGATATCAGATC
                      ******  *      * **    * ****  ***** *  *** * **** *  *** *

Gallus gallus VIT2    TGCTTTTCCAGAAGGTCAAGAGATGCCAGTTTCATACAGAATACGTATTTGCACAAATTA
O. anatinus VIT       TGCTTTTCCATCAAATCCTCCAGTGCCTCATTCATCAGTCCTGCCCTTCTTTACAAAGTC
                      **********  *  **      ****   *****      * *   * *  ***** *
```

```
Gallus gallus VIT2     ATTGGAGAACATGAAGCTAAAATAGTTTTGATGCCAGTTCATACAGATGCTGATATTGAC
O. anatinus VIT        ATCGGAGAACATGCCTTTAAAGTGGTTGTGAAACCAGTTTCTGCAGCTAATACAATTGAC
                       ** **********    **** * *** ***  ******  * *** *  *   ******

Gallus gallus VIT2     AAAATTCAGCTGGAGATTCAGGCAGGATCTAGAGCAGCTGCCAGAATAATTACTGAGGTA
O. anatinus VIT        AAGATCCAGTTGGAGATTCAGACAGGGCCCAAAGCGGCTTCGAAAATTATCCGCACCGTA
                       ** ** *** *********** ****  * * *** *** * * *** **       ***

Gallus gallus VIT2     AACCCAGAGTCTGAGGAA------------------------------------GAGGAT
O. anatinus VIT        ACTGCTGGGGACGCGGATGTTGACGATCGCAAACACGAGGATGGTGACGGCAGTGATGAA
                       *   * * *   * ***                                     ** **

Gallus gallus VIT2     GAATCATCTCCATATGAGGACATTCAAGCTAAACTGAAGAGGATTCTAGGCATTGACAGT
O. anatinus VIT        GACATCTCGCACGATGAGGCCGTGCAACTGAAATTGAAAAAGATCCTAGGAATCGATAAA
                       **    ** *   ****** * * ***   *** **** * *** ***** ** ** *

Gallus gallus VIT2     ATGTTCAAGGTTGCAAACAAAACACGGCACCCGAAAAATCGACCATCTAAGAAAGGAAAC
O. anatinus VIT        GTTTTTCAGNNNNNNNNNNNNNNNNNNNNNNNNNNNNNNNNNNNNNNNNNNNNNNNNNNN
                        * **  **

Gallus gallus VIT2     ACTGTGCTAGCAGAGTTTGGGACAGAGCCTGATGCAAAAACTTCCTCCAGCTCATCTTCT
O. anatinus VIT        NNNNNNNNNNNNNNNNNNNNNNNNNNNNNNNNNNNNNNNNNNNNNNNNNNNNNNNNNNNN

Gallus gallus VIT2     GCCTCCTCAACTGCCACCTCTTCTTCCTCATCATCTGCCTCCTCTCCTAATCGTAAAAAG
O. anatinus VIT        NNNNNNNNNNNNNNNNNNNNNNNNNNNNNNNNNNNNNNNNNNNNNNNNNNNNNNNNNNNN

Gallus gallus VIT2     CCTATGGATGAAGAGGAGAATGATCAAGTAAAGCAAGCAAGAAACAAAGATGCAAGCAGC
O. anatinus VIT        NNNNNNNNNNNNNNNNNNNNNNNNNNNNNNNNNNNNNNNNNNNNNNNNNNNNNNNNNNNN

Gallus gallus VIT2     AGCAGCAGGAGCAGCAAGAGCAGTAACAGCAGCAAGAGAAGCAGCAGCAAGAGCAGTAAC
O. anatinus VIT        NNNNNNNNNNNNNNNNNNNNNNNNNNNNNNNNNNNNNNNNNNNNNNNNNNNNNNNNNNNN

Gallus gallus VIT2     AGCAGCAAGAGAAGCAGCAGCAGCAGTAGCAGTAGCAGTAGCAGCAGCAGGAGCAGCAGC
O. anatinus VIT        NNNNNNNNNNNNNNNNNNNNNNNNNNNNNNNNNNNNNNNNNNNNNNNNNNNNNNNNNNNN

Gallus gallus VIT2     AGTAGCAGCAGTAGTAGCAGTAACAGCAAGAGCAGCAGTAGCAGCAGCAAGAGCAGCAGT
O. anatinus VIT        NNNNNNNNNNNNNNNNNNNNNNNNNNNNNNNNNNNNNNNNNNNNNNNNNNNNNNNNNNNN

Gallus gallus VIT2     AGCAGCAGCAGGAGCAGAAGCAGCAGCAAGAGCAGCAGTAGCAGCAGCAGCAGTAGCAGC
O. anatinus VIT        NNNNNNNNNNNNNNNNNNNNNNNNNNNNNNNNNNNNNNNNNNNNNNNNNNNNNNNNNNNN

Gallus gallus VIT2     AGCAGCAGCAGCAAAAGTAGCAGTAGTAGGAGCAGTAGCAGCAGCAGCAAGTCAAGCAGT
O. anatinus VIT        NNNNNNNNNNNNNNNNNNNNNNNNNNNNNNNNNNNNNNNNNNNNNNNNNNNNNNNNNNNN
```

```
Gallus gallus VIT2      CACCATAGCCATAGCCATCATTCAGGGCATCTAAATGGCAGCAGCAGCAGCAGCAGCAGC
O. anatinus VIT         NNNNNNNNNNNNNNNNNNNNNNNNNNNNNNNNNNNNNNNNNNNNNNNNNNNNNNNNNNNN


Gallus gallus VIT2      AGCAGGTCAGTGAGTCACCACAGCCATGAGCATCACTCAGGACATCTGGAAGATGACAGC
O. anatinus VIT         NNNNNNNNNNNNNNNNNNNNNNNNNNNNNNNNNNNNNNNNNNNNNNNNNNNNNNNNNNNN


Gallus gallus VIT2      AGTAGCAGCAGCAGCAGCAGCGTGCTTTCCAAAATATGGGGGCGTCATGAGATTTATCAG
O. anatinus VIT         NNNNNNNNNNNNNNNNNNNNNNNNNNNNNNNNNNNNNNNGGACCCCTGGAGATTTACCAG
                                                               ** *  *  ******** ***

Gallus gallus VIT2      TATCGCTTTAGATCAGCACACAGACAAGAGTTCCCCAAAAGAAAACTCCCAGGTGACCGC
O. anatinus VIT         TACAAATTCAAATCTGCACACAAGCAAGAGNNNNNNNNNNNNNNNNNNNNNNNNNNNNNN
                        **    ** * *** *******  ******

Gallus gallus VIT2      GCTACCAGCAGATACTCCTCTACCAGATCCAGCCATGACACATCCCGAGCTGCTTCCTGG
O. anatinus VIT         NNNNNNNNNNNNNNNNNNNNNNNNNNNNNNNNNNNNNNNNNNNNNNNNNNNNNNNNNNNN


Gallus gallus VIT2      CCTAAGTTTCTGGGTGACATCAAAACCCCAGTGTTAGCTGCTTTTCTCCATGGCATTAGT
O. anatinus VIT         CCCAAATTCCTGGGAGACAGTCATCCCCCAGCCGTGGCCGCCGTCTTCCGCGCTTTCCGA
                        ** ** ** ***** ****   *  ******   * ** **  *  ***  *   *  *

Gallus gallus VIT2      AACAATAAGAAGACAGGAGGCCTCCAGCTTGTGGTATATGCTGATACCGACTCGGTCAGG
O. anatinus VIT         AGCGACAAGAAGCAGGCTGGTTACCAGTTTATGATGTACACAGACACACACTCCTCCAGA
                        * * * ******   *  **   **** ** ** * **  * ** **  ****   ***

Gallus gallus VIT2      CCGCGGGTGCAGGTATTTGTGACAAACCTCACAGATTCGAGCAAGTGGAAGCTCTGCGCA
O. anatinus VIT         CCCAGGATGCAGTTATTTTTCTCCAGCATCTCCGCCACCAGCAAGTGGAAGTTCTGTGCC
                        **  ** ***** ***** *  * * * ** * *   * ************ **** **

Gallus gallus VIT2      GATGCTTCGGTCCGCAATGCTCACAAGGCAGTGGCCTACGTGAAATGGGGCTGGGACTGC
O. anatinus VIT         GATGCTTTAGTTGTTAACACCCACAAGGCAGCGGCGTCTCTGAAGTGGGGTCAGAACTGC
                        *******  **    **  * ********** *** *   **** *****   * *****

Gallus gallus VIT2      CGGGACTACAAGGTTTCTACTGAGCTGGTAACTGGGCGGTTTGCTGGGCACCCTGCTGCA
O. anatinus VIT         CGGCAGTATAAAGTGGCTACCAAGGTTGTGACCGGCCGGTTTGCTGACCACCCCGCTCTG
                        *** * ** ** **  ****  ** * ** ** ** **********  ***** ***

Gallus gallus VIT2      CAAGTGAAGCTGGAGTGGCCCAAGGTTCCTTCGAATGTCAGATCAGTGGTTGAATGGTTT
O. anatinus VIT         CAAGTCAAAGCAGAGTGGCCCAAAGTCTCCTCACAGATCCGAAAGGCTGCAAACTGGTTT
                        ***** **    *********** **  * **  *  ** **   *  *   * ******

Gallus gallus VIT2      TACGAGTTTGTCCCTGGGGCTGCATTTATGCTGGGTTTCTCTGAGAGAATGGACAAGAAT
O. anatinus VIT         TACCCATTCTTTCCAGGATTAGCGTACATGTTTGGATTCTACGGGAAAGAATGGAAAAAC
                        ***   **  * ** **    ** *  *** * ** ****  * ** *      ** **

Gallus gallus VIT2      CCTTCTCGACAAGCCAGGATGGTTGTGGCTCTAACTTCTCCGAGGACATGTGATGTTGTT
O. anatinus VIT         CCGTCTCGACAGGCCACTTTTGTGGTGTCTGCAACTTCCGCGAGAACGTCCGCTGTTGTG
                        ** ******** ****   * ** *** **  ******  **** ** *  * ******
```

```
Gallus gallus VIT2    GTCAAGCTGCCTGATATAATCCTCTATCAAAAAGCCGTGAGGCTTCCTCTATCACTCCCT
O. anatinus VIT       ATCAAACTTCCTGACTTCACTATTTATGACAGAGCCATTTGGCTCCCATTCTCAGTCCCT
                       **** ** *****  * *   * *** * * **** *  **** **  * *** *****

Gallus gallus VIT2    GTGGGTCCAAGGATCCCAGCTTCAGAGCTGCAGCCTCCAATC---TGGAACGTCTTTGCT
O. anatinus VIT       GTAGGACCGCACGTTTCACCT---TCCACTCAGCCACCAGCTCCCTGGAACTTCTTCTCT
                      ** ** **     *  ** **         ***** ***      ****** ****  **

Gallus gallus VIT2    GAAGCCCCCTCTGCCGTGCTCGAGAATTTGAAAGCTCGCTGCTCAGTTTCGTACAACAAG
O. anatinus VIT       GAAGCCTCGTCGTTATTCGTAGAGAACCTTAAAGCTCGCTGTTATGCCGAAGGAAATAAT
                      ****** * **     *  * *****  * *********** *  *        ** **

Gallus gallus VIT2    ATCAAAACCTTTAATGAAGTCAAGTTCAACTACTCGATGCCCGCAAACTGCTATCACATC
O. anatinus VIT       ATCACCACCTTCAATGGAATTAACTACAACTACGAGATGCTTGCAAAGTGCTACCATGTC
                      ****  ***** **** * * ** * *******  *****  ***** ***** **  **

Gallus gallus VIT2    TTGGTTCAGGATTGCAGCTCTGAACTTAAGTTCCTGGTGATGATGAAAAGTGCTGGAGAA
O. anatinus VIT       TTAGCCCAGGACGGCAGCCAGGATCCAAAATTCATGGTGATGATAAAGAAAGTGGAAGAA
                      ** *  *****  *****   ** *  ** *** ********** ** *  *  * ****

Gallus gallus VIT2    GCTACAAACCTTAAAGCCATCAACATCAAGATTGGCAGTCATGAAATTGATATGCATCCT
O. anatinus VIT       TCGACCGACGGCAAGGCCATCAGCGTCAAACTGGGCAATCANNNNNNNNNNNNNNNNNNN
                       * **  **   ** ******* * ****  * **** ***

Gallus gallus VIT2    GTGAATGGACAGGTGAAACTGCTGGTAGATGGGGCTGAGAGCCCCACAGCCAACATTTCC
O. anatinus VIT       NNNNNNNNNNNNNNNNNNNNNNNNNNNNNNNNNNNNNNNNNNNNNNNNNNNNNNNNNNNN

Gallus gallus VIT2    CTCATATCTGCTGGTGCTTCTCTGTGGATTCACAATGAAAACCAAGGGTTTGCACTTGCT
O. anatinus VIT       NNNNNNNNNNNNNAACCAACCGTACTCATTGAGAAGGAGAAAAATGGCGTAACCCTCATT
                                      *  *  *    *** * ** ** **  * **  *  * **   *

Gallus gallus VIT2    GCCCCAGGCCATGGTATCGATAAATTGTACTTCGATGGAAAAACAATCACGATTCAAGTT
O. anatinus VIT       GCTCCAGCATATGGCATCGACAGACTTTTCTACGACGGAAAGGCCTGCAAGGTTCAAGTG
                      ** ****   **** ***** * * * * ** *** *****  *   ** * *******

Gallus gallus VIT2    CCTTTATGGATGGCAGGGAAAACATGTGGAATCTGTGGAAAATATGATGCAGAATGCGAA
O. anatinus VIT       TCACTGTGGATGATTGGAAAGACCCAAGGCATGTGTGGCAGCTGCGACGCTGAACATAAC
                       *  * ******   ** ** **    ** ** ***** *  *  ** ** ***    *

Gallus gallus VIT2    CAGGAGTATCGGATGCCCAATGGATATCTAGCTAAAAATGCCGTGAGCTTTGGTCATTCT
O. anatinus VIT       CTGGAATTCCAAATGCCCAATGGCCAGCTAGCTAAAAATGCAGTGAGTTTTGCCCAGTCT
                      * *** *  *  ***********  * ************** ***** ****  ** ***

Gallus gallus VIT2    TGGATCTTGGAAGAAGCGCCCTGTAGAGGAGCTTGTAAACTGCATCGTTCATTCGTGAAG
O. anatinus VIT       TGGATTTTAGCAGAAGATTCCTGTGATTCGGCTTGTGCCCTGCAGCGCAAAGTCATGAAA
                      ***** ** * *****   *****      ******   ***** **   * ** ****

Gallus gallus VIT2    CTTGAGAAGACGGTTCAGCTGGCGGGTGTTGATTCCAAGTGCTACTCTACAGAGCCTGTG
O. anatinus VIT       ATTGAGAAACGTGTTGGATATGAAAGACGGTCCTCCACCTGCCTGTCCATCCACCCCGTC
                       *******    ***      *   *       ****  ***   ** *   * ** **
```

```
Gallus gallus VIT2     CTGCGCTGTGCAAAGGGATGCTCTGCTACCAAGACAACTCCAGTAACTGTTGGCTTCCAC
O. anatinus VIT        TTGACGTGTCGAGAGGGATGCTCACCCACCAGAACTGCCCCCTTCGCCGTCGGTTTCCAC
                        **   *** * **********  * ****  **  * **  *  * ** ** ******

Gallus gallus VIT2     TGCCTCCCAGCTGATTCAGCTAACAGCCTAACTGACAAACAGATGAAGTACGACCAGAAG
O. anatinus VIT        TGTCTGACAGCTGGATCCGCTTCTGCTCTCCCTGACGAGCACAGTAACTTGGACCAGAAA
                       ** **  ******  ** ***      **  ***** * ** *  ** *  ******** 

Gallus gallus VIT2     TCAGAAGACATGCAGGATACTGTTGATGCACACACAACGTGTTCATGTGAGAATGAGGAA
O. anatinus VIT        TCCGAAGATTTGGTAGAAACCGTCGATGCCCACGCGACGTGCGCCTGTAGTAAACTACAG
                       ** *****  **   ** ** ** ***** *** * *****  * ***   **     * 

Gallus gallus VIT2     TGCAGTACATGA
O. anatinus VIT        TGTGCCGCGTGA
                       **     * ***
```
