# Supplementary material for: Loss of Egg Yolk Genes in Mammals and the Origin of Lactation and Placentation
Source: PLoS Biol. 2008 Mar 18;6(3):e63. doi: 10.1371/journal.pbio.0060063 (PMC2267819; doi:10.1371/journal.pbio.0060063)
Supplement: Supplementary file 6 [file pbio.0060063.sg006.pdf]

Figure S6

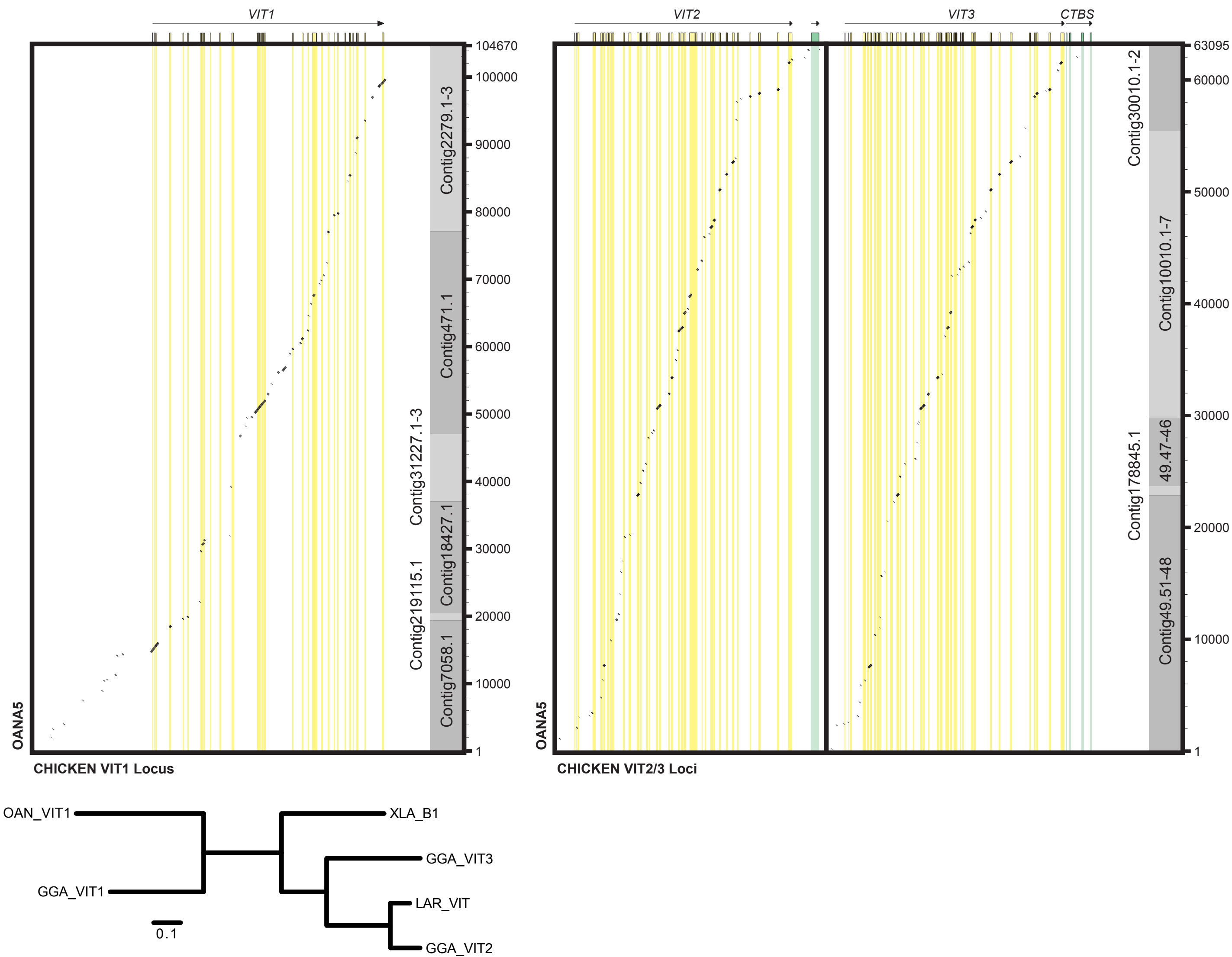

VIT1
104670
100000
90000
80000
70000
60000
50000
40000
30000
20000
10000
1
Contig2279.1-3
Contig471.1
Contig31227.1-3
Contig18427.1
Contig219115.1
Contig7058.1
OANA5
CHICKEN VIT1 Locus
VIT2
VIT3
CTBS
63095
60000
50000
40000
30000
20000
10000
1
Contig30010.1-2
Contig10010.1-7
49.47-46
Contig178845.1
Contig49.51-48
OANA5
CHICKEN VIT2/3 Loci
OAN_VIT1
GGA_VIT1
XLA_B1
GGA_VIT3
LAR_VIT
GGA_VIT2
0.1
