# Supplementary material for: Loss of Egg Yolk Genes in Mammals and the Origin of Lactation and Placentation
Source: PLoS Biol. 2008 Mar 18;6(3):e63. doi: 10.1371/journal.pbio.0060063 (PMC2267819; doi:10.1371/journal.pbio.0060063)
Supplement: Supplementary file 7 [file pbio.0060063.sg007.pdf]

Figure S7

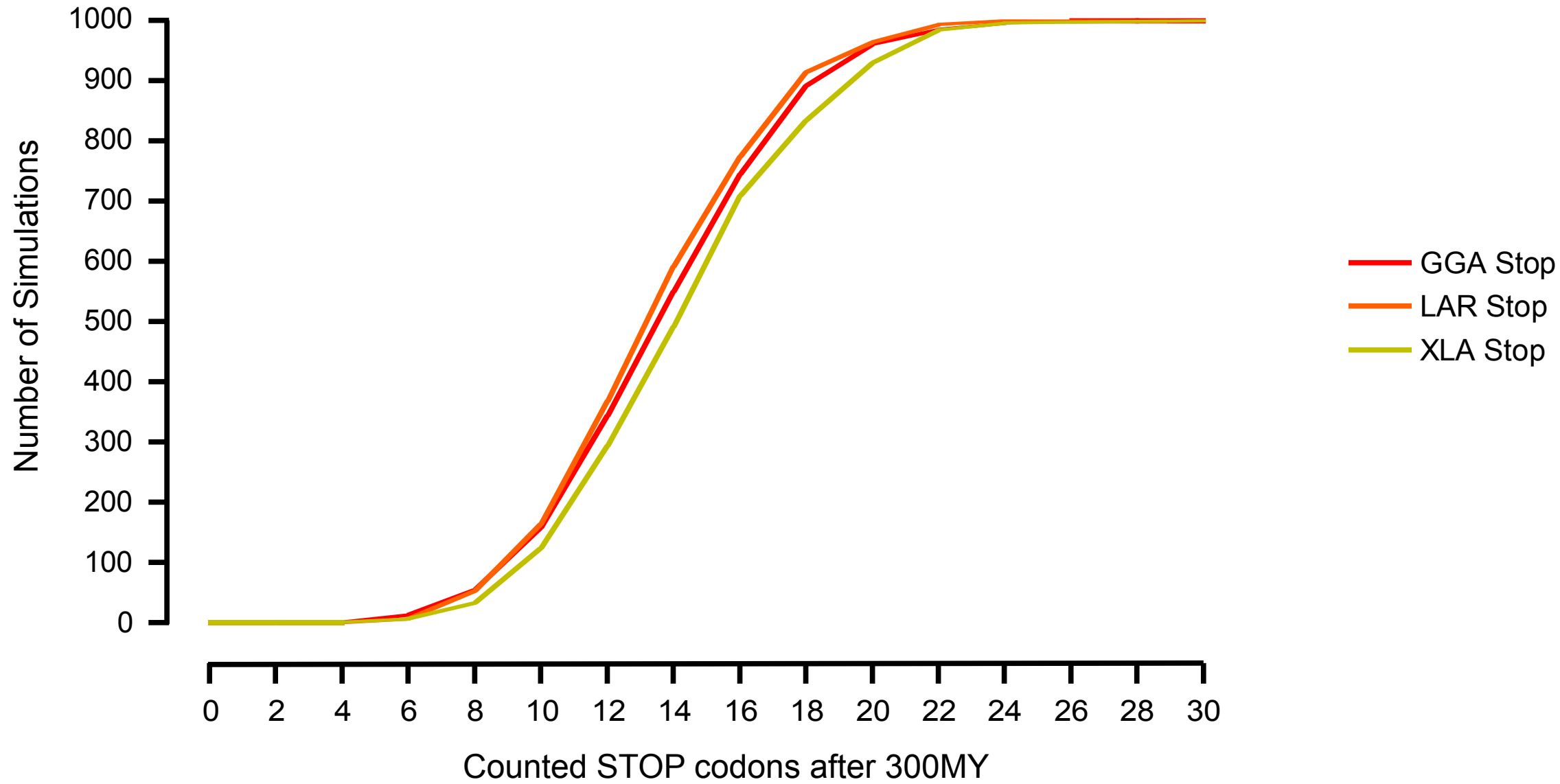

1000
900
800
700
600
500
400
300
200
100
0
Number of Simulations
0
2
4
6
8
10
12
14
16
18
20
22
24
26
28
30
Counted STOP codons after 300MY
GGA Stop
LAR Stop
XLA Stop
