# Supplementary material for: Loss of Egg Yolk Genes in Mammals and the Origin of Lactation and Placentation
Source: PLoS Biol. 2008 Mar 18;6(3):e63. doi: 10.1371/journal.pbio.0060063 (PMC2267819; doi:10.1371/journal.pbio.0060063)
Supplement: Supplementary file 8 [file pbio.0060063.sg008.pdf]

Figure S8

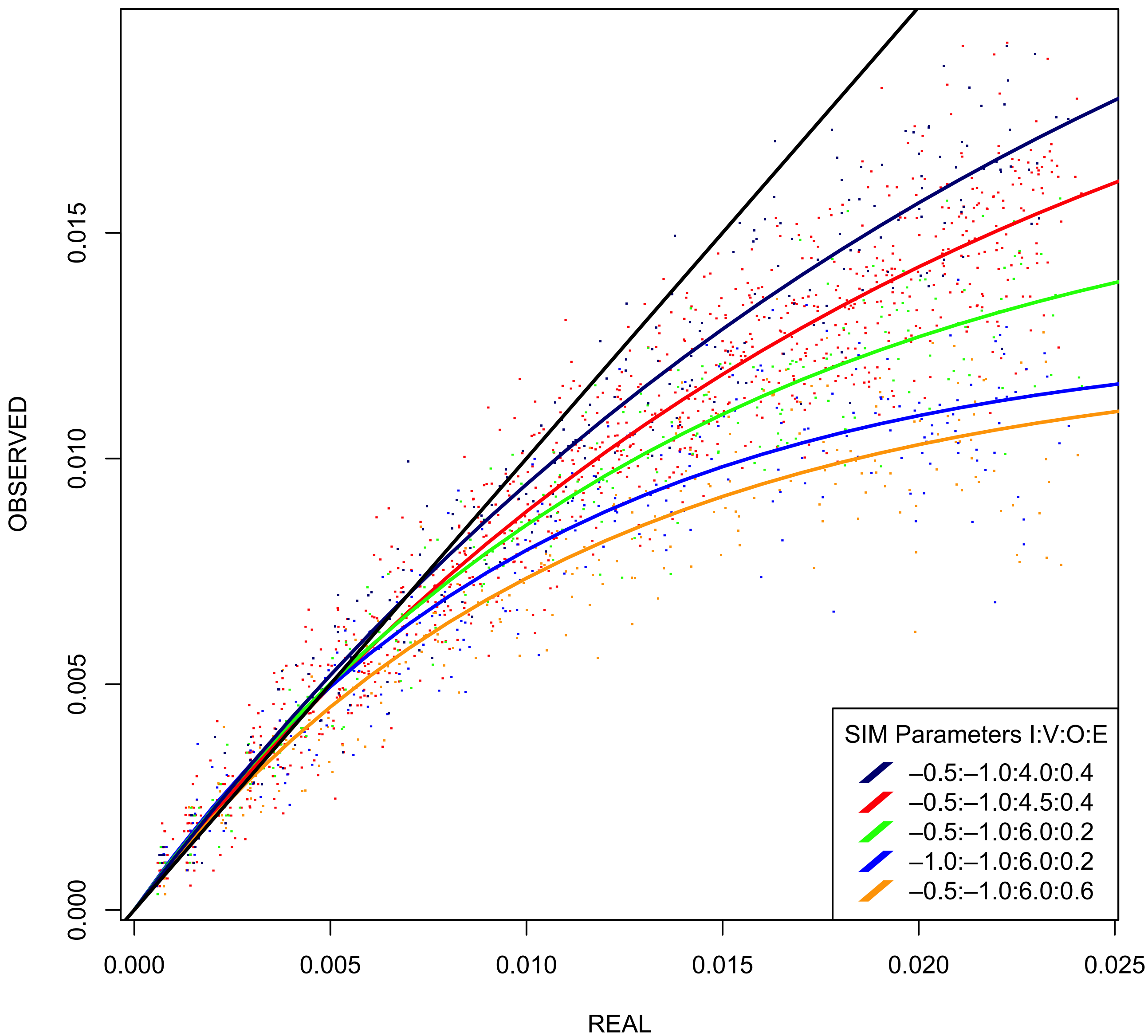

OBSERVED
0.000
0.005
0.010
0.015
REAL
0.000
0.005
0.010
0.015
0.020
0.025
SIM Parameters I:V:O:E
−0.5:−1.0:4.0:0.4
−0.5:−1.0:4.5:0.4
−0.5:−1.0:6.0:0.2
−1.0:−1.0:6.0:0.2
−0.5:−1.0:6.0:0.6
